# 100 ancient genomes show repeated population turnovers in Neolithic Denmark

**DOI:** 10.1038/s41586-023-06862-3

**Published:** 2024-01-10

**Authors:** Morten E. Allentoft, Martin Sikora, Anders Fischer, Karl-Göran Sjögren, Andrés Ingason, Ruairidh Macleod, Anders Rosengren, Bettina Schulz Paulsson, Marie Louise Schjellerup Jørkov, Maria Novosolov, Jesper Stenderup, T. Douglas Price, Morten Fischer Mortensen, Anne Birgitte Nielsen, Mikkel Ulfeldt Hede, Lasse Sørensen, Poul Otto Nielsen, Peter Rasmussen, Theis Zetner Trolle Jensen, Alba Refoyo-Martínez, Evan K. Irving-Pease, William Barrie, Alice Pearson, Bárbara Sousa da Mota, Fabrice Demeter, Rasmus A. Henriksen, Tharsika Vimala, Hugh McColl, Andrew Vaughn, Lasse Vinner, Gabriel Renaud, Aaron Stern, Niels Nørkjær Johannsen, Abigail Daisy Ramsøe, Andrew Joseph Schork, Anthony Ruter, Anne Birgitte Gotfredsen, Bjarne Henning Nielsen, Erik Brinch Petersen, Esben Kannegaard, Jesper Hansen, Kristoffer Buck Pedersen, Lisbeth Pedersen, Lutz Klassen, Morten Meldgaard, Morten Johansen, Otto Christian Uldum, Per Lotz, Per Lysdahl, Pernille Bangsgaard, Peter Vang Petersen, Rikke Maring, Rune Iversen, Sidsel Wåhlin, Søren Anker Sørensen, Søren H. Andersen, Thomas Jørgensen, Niels Lynnerup, Daniel J. Lawson, Simon Rasmussen, Thorfinn Sand Korneliussen, Kurt H. Kjær, Richard Durbin, Rasmus Nielsen, Olivier Delaneau, Thomas Werge, Kristian Kristiansen, Eske Willerslev

**Affiliations:** 1 Lundbeck Foundation GeoGenetics Centre, Globe Institute, University of Copenhagen, Copenhagen, Denmark; 2 Trace and Environmental DNA (TrEnD) Laboratory, School of Molecular and Life Sciences, Curtin University, Perth, Western Australia Australia; 3 Cluster of Excellence ROOTS, Kiel University, Kiel, Germany; 4 Sealand Archaeology, Kalundborg, Denmark; 5 Department of Historical Studies, Gothenburg University, Göteborg, Sweden; 6 Institute of Biological Psychiatry, Mental Health Center Sct. Hans, Copenhagen University Hospital, Copenhagen, Denmark; 7 GeoGenetics Group, Department of Zoology, University of Cambridge, Cambridge, UK; 8 Research Department of Genetics, Evolution and Environment, University College London, London, UK; 9 Laboratory of Biological Anthropology, University of Copenhagen, Copenhagen, Denmark; 10 Laboratory for Archaeological Chemistry, Department of Anthropology, University of Wisconsin–Madison, Madison, WI USA; 11 The National Museum of Denmark, Copenhagen, Denmark; 12 Department of Geology, Lund University, Lund, Sweden; 13 Tårnby Gymnasium og HF, Kastrup, Denmark; 14 Globe Institute, Faculty of Health and Medical Sciences, University of Copenhagen, Copenhagen, Denmark; 15 Department of Genetics, University of Cambridge, Cambridge, UK; 16 Department of Computational Biology, University of Lausanne, Lausanne, Switzerland; 17 Swiss Institute of Bioinformatics, University of Lausanne, Lausanne, Switzerland; 18 Eco-anthropologie (EA), Dpt ABBA, Muséum National d’Histoire Naturelle, CNRS, Université Paris Cité, Musée de l’Homme, Paris, France; 19 Center for Computational Biology, University of California, Berkeley, USA; 20 Department of Health Technology, Section of Bioinformatics, Technical University of Denmark, Kongens Lyngby, Denmark; 21 Department of Archaeology and Heritage Studies, Aarhus University, Aarhus, Denmark; 22 Neurogenomics Division, The Translational Genomics Research Institute (TGEN), Phoenix, AZ USA; 23 Vesthimmerlands Museum, Aars, Denmark; 24 The Saxo Institute, University of Copenhagen, Copenhagen, Denmark; 25 Museum Østjylland, Randers, Denmark; 26 Svendborg Museum, Svendborg, Denmark; 27 Museum Sydøstdanmark, Vordingborg, Denmark; 28 HistorieUdvikler, Kalundborg, Denmark; 29 Department of Health and Nature, University of Greenland, Nuuk, Greenland; 30 The Viking Ship Museum, Roskilde, Denmark; 31 Museum Nordsjælland, Hillerød, Denmark; 32 Museum Vestsjælland, Holbæk, Denmark; 33 Vendsyssel Historiske Museum, Hjørring, Denmark; 34 Moesgaard Museum, Højbjerg, Denmark; 35 Laboratory of Biological Anthropology, Department of Forensic Medicine, University of Copenhagen, Copenhagen, Denmark; 36 Institute of Statistical Sciences, School of Mathematics, University of Bristol, Bristol, UK; 37 Novo Nordisk Foundation Centre for Protein Research, Faculty of Health and Medical Sciences, University of Copenhagen, Copenhagen N, Denmark; 38 Wellcome Sanger Institute, Wellcome Trust Genome Campus, Cambridge, UK; 39 Department of Integrative Biology and Statistics, UC Berkeley, Berkeley, CA USA; 40 Department of Clinical Medicine, University of Copenhagen, Copenhagen, Denmark; 41 MARUM Center for Marine Environmental Sciences and Faculty of Geosciences, University of Bremen, Bremen, Germany

**Keywords:** Population genetics, Archaeology, Genomics, History

## Abstract

Major migration events in Holocene Eurasia have been characterized genetically at broad regional scales^1–4^. However, insights into the population dynamics in the contact zones are hampered by a lack of ancient genomic data sampled at high spatiotemporal resolution^5–7^. Here, to address this, we analysed shotgun-sequenced genomes from 100 skeletons spanning 7,300 years of the Mesolithic period, Neolithic period and Early Bronze Age in Denmark and integrated these with proxies for diet (^13^C and ^15^N content), mobility (^87^Sr/^86^Sr ratio) and vegetation cover (pollen). We observe that Danish Mesolithic individuals of the Maglemose, Kongemose and Ertebølle cultures form a distinct genetic cluster related to other Western European hunter-gatherers. Despite shifts in material culture they displayed genetic homogeneity from around 10,500 to 5,900 calibrated years before present, when Neolithic farmers with Anatolian-derived ancestry arrived. Although the Neolithic transition was delayed by more than a millennium relative to Central Europe, it was very abrupt and resulted in a population turnover with limited genetic contribution from local hunter-gatherers. The succeeding Neolithic population, associated with the Funnel Beaker culture, persisted for only about 1,000 years before immigrants with eastern Steppe-derived ancestry arrived. This second and equally rapid population replacement gave rise to the Single Grave culture with an ancestry profile more similar to present-day Danes. In our multiproxy dataset, these major demographic events are manifested as parallel shifts in genotype, phenotype, diet and land use.

## Abstract (web summary)

Integrated data, including 100 human genomes from the Mesolithic, Neolithic and Early Bronze Age periods show that two major population turnovers occurred over just 1,000 years in Neolithic Denmark, resulting in dramatic changes in the genes, diet and physical appearance of the local people, as well as the landscape in which they lived.

## Main

The Mesolithic and Neolithic periods in southern Scandinavia are marked by a number of pivotal and well-described cultural transitions^8^. However, the genetic and demographic impacts of these events remain largely uncharacterized. The early postglacial human colonization of the Scandinavian peninsula (Sweden and Norway) is believed to comprise at least two distinct migration waves: a source related to western European hunter-gatherers (WHG) from the south, and an eastern European hunter-gatherer (EHG) source into the far north, before venturing south along the Atlantic coast of Norway^9,10^. However, insight into the fine-scale structure and mobility of Scandinavian Mesolithic populations is limited, including an almost complete absence of genetic data from southern Scandinavian populations associated with the consecutive Maglemose, Kongemose and Ertebølle cultures in Denmark.

The Neolithic transition represents a watershed event in European prehistory, marked by the spread of domesticated crops and livestock from Southwest Asia, starting around 11,000 bp. Although migrations and population turnovers associated with this transition have been demonstrated at broad geographical and chronological scales^1–4^, coarse sampling and a one-sided focus on genetics have hindered insights on social interaction and detailed demographic processes in the contact zones between locals and newcomers^5–7^. Southern Scandinavia occupies an enigmatic position in this discussion. The Neolithic transition was delayed here by a millennium compared to Central Europe, during which hunter-gatherer societies continued to flourish until around 5,900 calibrated years bp (cal. bp), only marginally affected by farmer populations to the south^11^. The substantial delay could suggest that the transition to farming in Denmark occurred by a different mechanism involving a stronger element of cultural diffusion^12^ than the migration of people (demic diffusion) observed in the rest of Europe^13–15^.

An extensive archaeological record has indicated that the Funnel Beaker culture (FBC) thrived for the first millennium of the Neolithic in Denmark, before an apparent decline^16^ was followed by the appearance of the Single Grave culture (SGC). Owing to a lack of genetic data and a robust absolute chronology, the relation between the FBC and the SGC has been extensively debated^17–19^. Population dynamics associated with this second cultural transition in Neolithic Denmark are similarly unresolved, including its possible link to the ‘steppe migrations’ that transformed the gene pools elsewhere in Europe around the same time^1,2^.

**Fig. 1:**
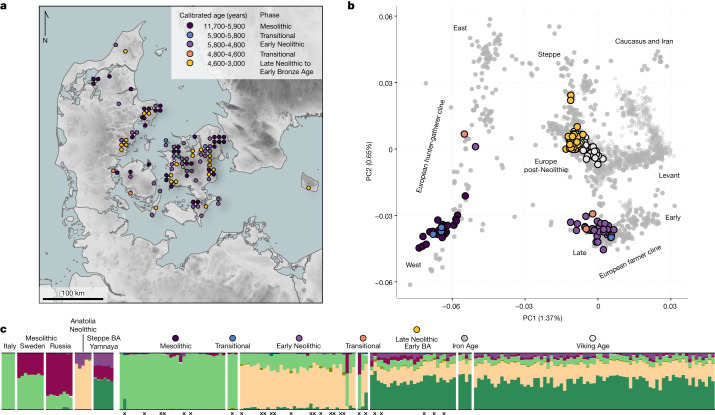
Overview of dataset. **a**, Geographic locations and age ranges relating to the 100 sequenced genomes from Denmark. Groupings are designated through a combination of chronology, culture, and ancestry (see Supplementary Notes 1 and 3). **b**, PCA for 179 ancient Danish individuals (Supplementary Data 3) ranging from the Mesolithic to the Viking Age, including previously published ones^1,47,57,76^, in the context of broader West Eurasian genetic diversity (*n* = 983 modern individuals, open grey circles; *n* = 1,105 ancient individuals, filled grey circles). Ancient individuals from Denmark are coloured according to the period as defined in **a** and **c**. **c**, Unsupervised model-based clustering (ADMIXTURE) for *K* = 8 ancestry components in Danish individuals, as well as contextual data from selected groups (left) that represent relevant ancestry components. See Extended Data Fig. 1 for individual labels. Black crosses indicate low-coverage genomes represented by pseudo-haploid genotypes. BA, Bronze Age.

To investigate these defining events at high temporal and spatial resolution, we analyse a detailed and continuous dataset of 100 ancient Danish shotgun-sequenced genomes (0.01× to 7.1× autosomal coverage^3^), spanning about 7,300 years from the Early Mesolithic Maglemose, the Kongemose and Late Mesolithic Ertebølle epochs, the Early and Middle Neolithic FBC and the SGC, up until the Bronze Age (Fig. 1 and Supplementary Data 1). The archaeological record in Denmark represents a very large assemblage of well-documented Mesolithic and Neolithic human skeletal remains, from a wide range of chronological, topographical and socio-cultural contexts. This is a result of an environment and climate that was amenable to both Mesolithic fisher-hunter-gatherer lifeways^20^ and the later Neolithic farming practices, combined with taphonomically favourable preservation conditions for skeletal remains, and a long, prolific history of archaeological research. We used a multiproxy approach, combining autosomal imputed genomes^3,21^ with Y chromosomal and mitochondrial haplogroups, ^14^C-dating, genetic phenotype predictions, as well as ^87^Sr/^86^Sr, δ^13^C and δ^15^N isotope data as proxies for mobility and diet. Moreover, to investigate a direct link between demographic and environmental processes, we align the genetic changes observed in the Danish population over time with changes in local vegetation, based on pollen analyses and quantitative vegetation cover reconstruction.

## The Mesolithic period

It is not known whether shifts in southern Scandinavian Mesolithic material culture occurred in a population continuum or were facilitated by incoming migrants. The Early Mesolithic settlement in Denmark is associated with the Maglemose culture (around 11,000–8,400 cal. bp), characterized archaeologically by small flint projectiles in geometric shapes. Until the recent development of underwater archaeology, this culture was known mainly from inland locations along lakes and rivers^22^. During the succeeding Kongemose culture (around 8,400–7,400 cal. bp), trapeze-shaped flint points dominate the assemblages of arrowheads^23^ along with high quality long blades. Most of the larger settlements cluster at good fishing locations along the coasts^24^, but there are also specialized hunting camps in the interior^25^. The Late Mesolithic Ertebølle culture (about 7,400–5,900 cal. bp), is characterized by flint points with transverse edges. Pottery was introduced from other hunter-gatherer groups to the east and perhaps the southwest^26^ and ‘exotic’ shaft-hole axes suggest exchange with farming societies south of the Baltic Sea^27^. The larger habitation sites, densely scattered along the coasts, probably represent multi-family, year-round occupation^24,28^ and they have provided important insights into the physical anthropology and spiritual culture of the period.

**Fig. 2:**
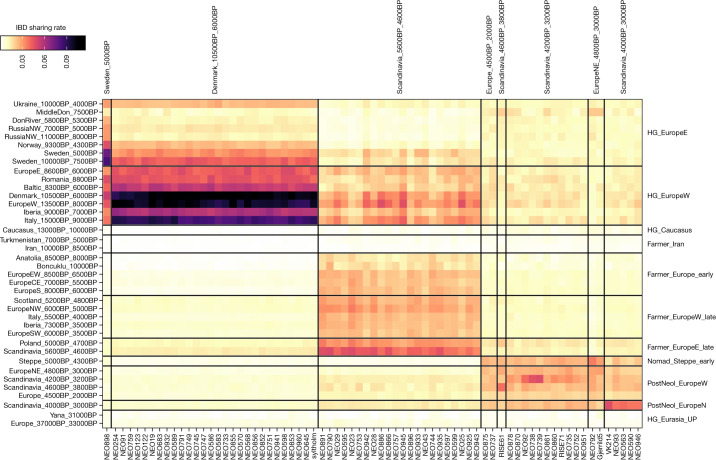
Identity-by-descent sharing patterns in ancient Danish individuals from circa 10,500–3000 cal. BP. Heat map showing relative IBD-sharing rate of 72 imputed ancient individuals from Denmark (*n* = 67 individuals reported in this Article, *n* = 5 previously published individuals^1,47,57,76^) from the Mesolithic to the Bronze Age with selected genetic clusters. Individuals are grouped by their genetic cluster membership. See Supplementary Data 3 for dataset and ancestry category definition.

**Fig. 3:**
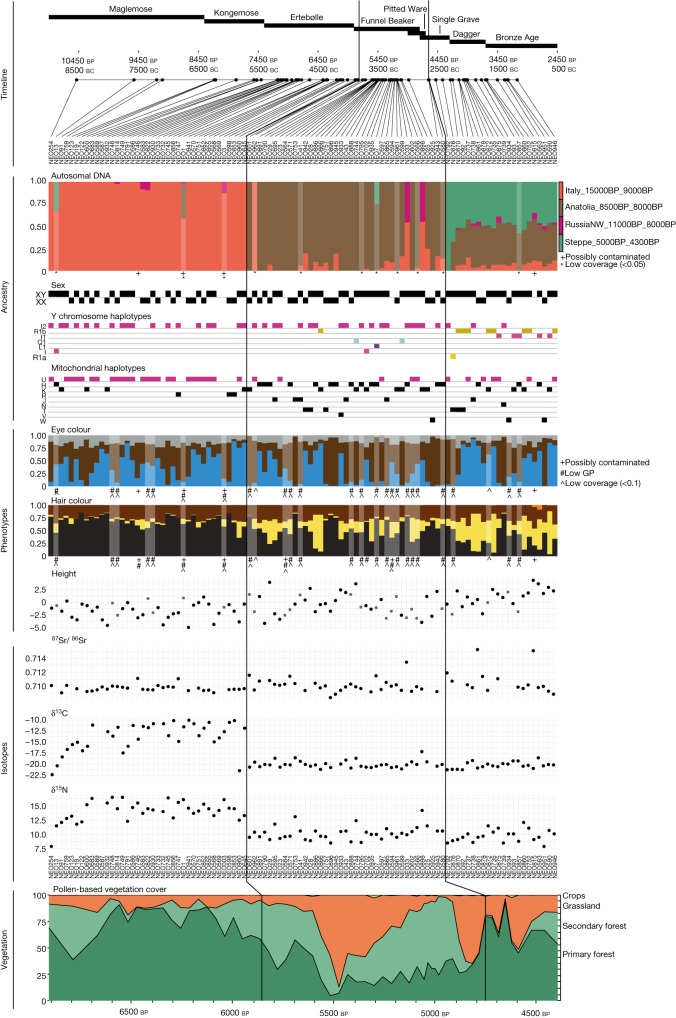
Genetic, phenotypic, dietary and environmental shifts in Denmark through time. Evidence of two population turnovers in chronologically sorted multiproxy data from 100 Danish Mesolithic, Neolithic and Early Bronze Age skeletons (Supplement Data 1). The figure shows concomitant changes in (from the top) admixture proportions in non-imputed genome-wide data, Y chromosomal and mitochondrial haplogroups, genetic phenotype predictions (based on imputed data) and ^87^Sr/^86^Sr and δ^13^C and δ^15^N isotope data as proxies for mobility and diet, respectively. Predicted height values represent differences (in cm) from the average height of the present-day Danish population; probabilities for the hair colours (blond, brown, black and red) and eye colours (blue and brown) are shown, with grey denoting probability of intermediate eye colour (including grey, green and hazel). Lower panel shows the quantitative changes in vegetation cover, based on pollen analyses at Lake Højby in Zealand. Note that the vegetation panel covers a shorter time interval than the other panels. Black vertical lines mark the first presence of Anatolian Neolithic farmer ancestry and Steppe-related ancestry, respectively. Individuals with low genomic coverage, signs of possible contamination and/or low genotype prediction score (GP) are indicated (Methods).

**Extended Data Fig. 1:**
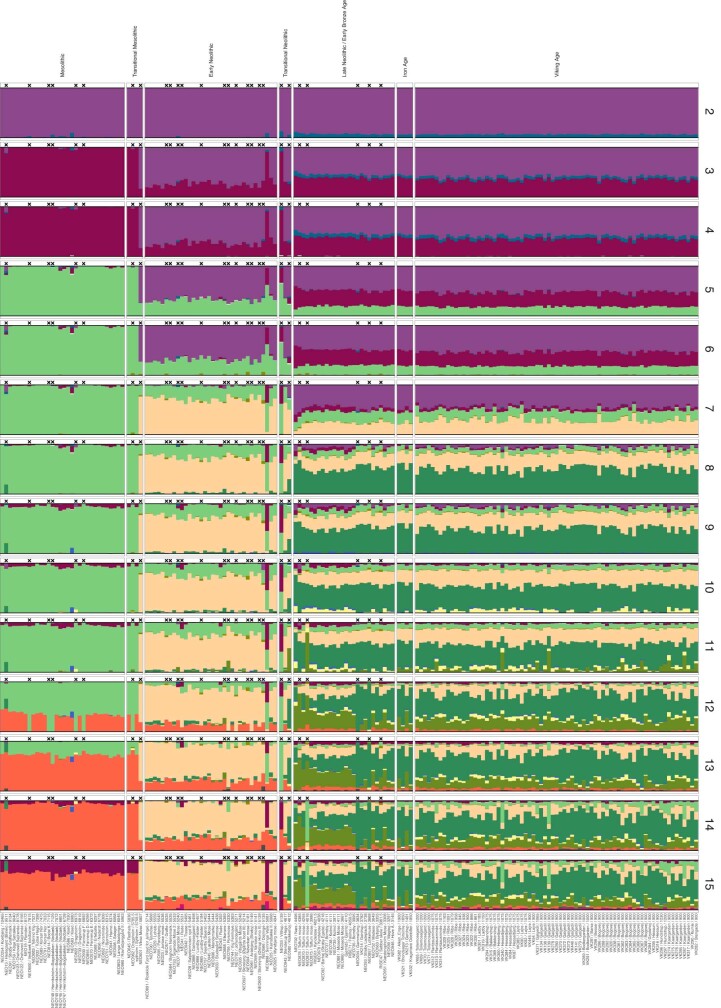
Model-based clustering. Unsupervised model-based clustering results (ADMIXTURE) for K = 2 to K = 15 assumed components for all published shotgun-sequenced ancient individuals from Denmark - including the herein presented 93 genomes (contamination <5% and close relative pairs excluded). Imputed genomes were used where available^3^. For low-coverage individuals (indicated with black cross) pseudo-haploid genotypes were used.

**Extended Data Fig. 2:**
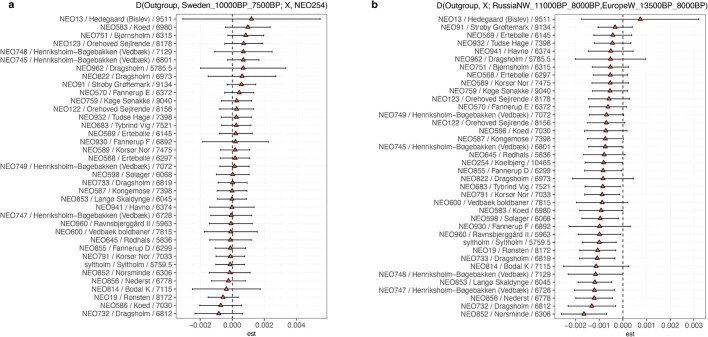
Allele sharing of Danish Mesolithic individuals. **a**, D-statistic testing whether Danish Mesolithic individuals form a clade with the earliest Danish Mesolithic individual in the dataset (NEO254, Koelbjerg Man) to the exclusion of a genetic cluster of Mesolithic hunter-gatherer individuals from Sweden (Sweden_10000BP_7500BP). **b**, D-statistic testing whether Danish Mesolithic individuals form a clade with a genetic cluster of Western European HG individuals (EuropeW_13500BP_8000BP) to the exclusion of a genetic cluster of Eastern European HG individuals (RussiaNW_11000BP_8000BP). Error bars indicate three standard errors.

**Extended Data Fig. 3:**
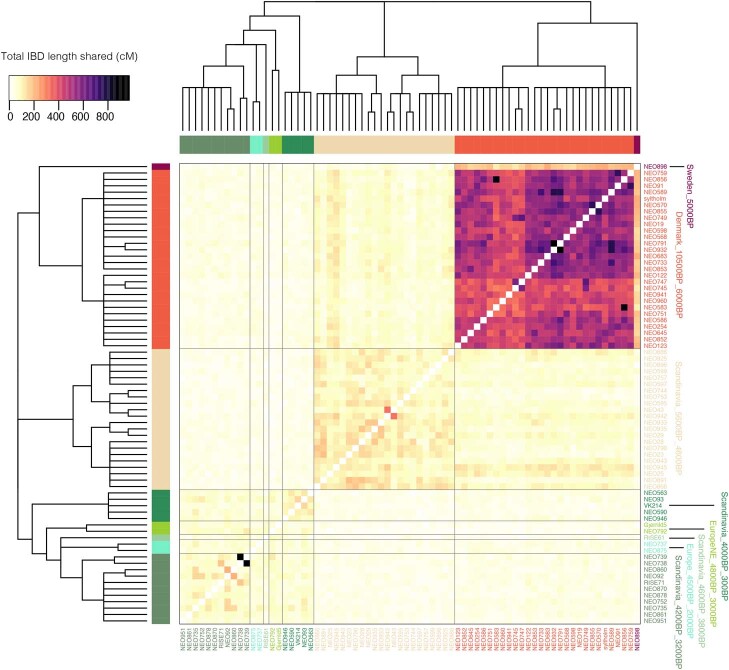
IBD sharing among ancient individuals from Denmark. Heatmap showing pairwise amount of total length of IBD shared between 72 ancient Danish individuals dated to older than 3,000 cal. BP. Colours in border and text indicate genetic cluster membership, and dendrograms show clustering hierarchy.

By analysing genomes from 38 Danish hunter-gatherers and inferring their ancestry, we examine whether cultural transitions observed in the Danish archaeological record are associated with any genetic changes in the population. Model-based clustering (ADMIXTURE), PCA and IBD-sharing analyses show that throughout the Maglemose (*n* = 4), Kongemose (*n* = 8) and Ertebølle (*n* = 27) epochs the region displayed a remarkable genetic homogeneity across a 4,500-year transect (Figs. 1–3 and Extended Data Figs. 1–3), supporting interpretations of demographic continuity favoured by some archaeologists^23–25^. From the earliest known skeleton in Denmark, ‘Koelbjerg Man’ (NEO254, 10,648–10,282 cal. bp^29^), to the most recent Mesolithic skeleton included here, ‘Rødhals Man’ (NEO645, 5,916–5,795 cal. bp), the individuals derive their ancestry almost exclusively from the same southern European source (Italy_15000BP_9000BP) that predominated in WHG ancestry in Mesolithic Western Europe^3^.

**Extended Data Fig. 4:**
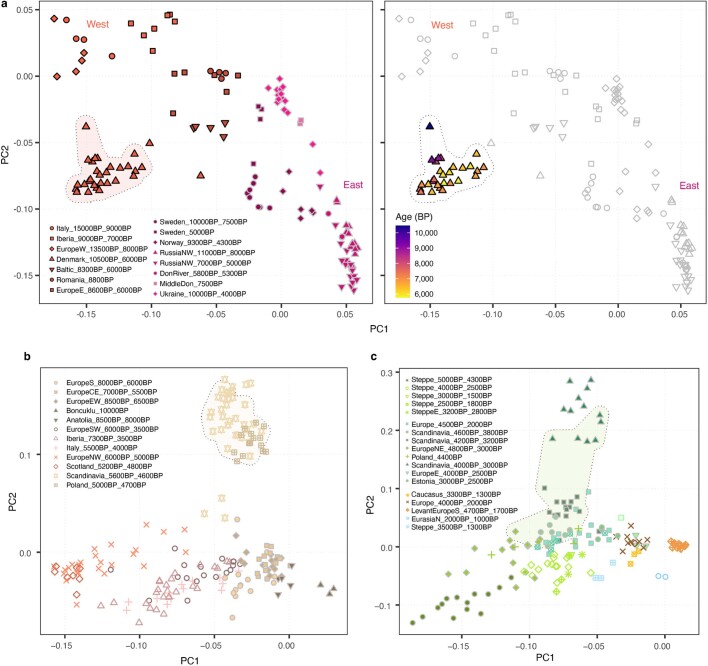
Genetic affinities of ancient individuals from Denmark. Panels show principal component analyses based on pairwise IBD-sharing of **a**, 30 imputed Danish Mesolithic individuals in context of 105 European HGs (right panel shows Danish individuals coloured by age); **b**, 22 imputed Danish early Neolithic individuals within the context of 170 Anatolian and European Neolithic farmers **c**, 21 imputed Danish LNBA individuals within the context of 127 European LNBA individuals. Symbol colour and shape indicate the genetic cluster of an individual (Supplementary Data III). The extent of PCA positions of individuals from Denmark are indicated with a dotted line hull. Ancestry cluster categories defined in^3^.

In the IBD-based principal components analysis (PCA), the Danish Mesolithic individuals cluster closely together (Extended Data Fig. 4a), but beyond this tight local genetic connection they share most recent ancestry with the geographically and temporally proximate hunter-gatherer individuals from Western Europe (such as Cheddar Man, Loschbour and Bichon, commonly referred to as WHG; genetic cluster EuropeW_13500BP_8000BP; Fig. 2). A subtle shift of the earliest Danish individuals towards these western individuals probably reflects their closer temporal proximity captured through IBD sharing (Extended Data Fig. 4a). Although pressure-debitage of blades in the Maglemosian culture and pottery in the Ertebølle culture are both argued to have an eastern origin^9,10,30,31^, our data show no evidence for admixture with more eastern hunter-gatherers during those times. This points to cultural diffusion as the source of these introductions in Denmark. When tested with D-statistics, all Danish Mesolithic individuals form a clade with the earliest individual (NEO254), to the exclusion of Swedish Mesolithic hunter-gatherers (Sweden_10000BP_7500BP; Extended Data Fig. 2a) despite the close proximity to Sweden. However, a weak signal of gene flow with EHGs was shared across the whole Danish Mesolithic transect (Extended Data Fig. 2b), suggesting contact with communities further to the east prior to their expansion into Denmark before or during the earliest Mesolithic.

Genetic phenotype predictions (Supplementary Note 2) indicate a high probability of blue eye pigmentation throughout the Mesolithic, consistent with previous findings^1,15,32^, showing that this feature was present already in the early Mesolithic but was not fixed in the population. The Mesolithic hunter-gatherers from Denmark all display high probability of brown or black hair and height predictions generally suggest slightly lower and/or less variable stature than in the succeeding Neolithic period. We caution, however, that the relatively large genetic distance to modern individuals included in the genome-wide association studies (GWAS) panel produces scores that are less applicable to Mesolithic individuals than to more recent groups^33^.

**Extended Data Fig. 5:**
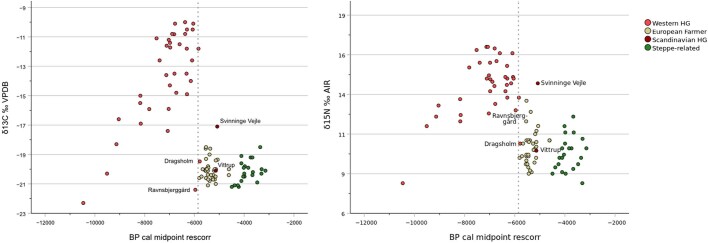
Dietary isotopic signatures. δ^13^C and δ^15^N values in bone/dentine samples from 100 ancient Danish individuals, coloured according to their main genetic ancestry group. A fundamental dietary and genetic shift is observed at the transition from the Mesolithic to the Neolithic c. 5,900 cal. BP (dashed line). Four anomalous individuals are highlighted. Data from^3^ and Supplementary Data II.

Stable isotope δ^13^C values in collagen can inform on the proportion of marine versus terrestrially-derived protein, whereas δ^15^N values reflect the trophic level of the protein sources^34^. The earliest skeleton (NEO254) shows depleted dietary isotopic values (Fig. 3) representing a lifestyle of inland hunter-gatherers of the Early Mesolithic. This result is mirrored in the second earliest known skeleton from Denmark (Tømmerupgårds Mose^34^). From later Maglemose (around 9,500 cal. BP) and throughout the Kongemose and Ertebølle epochs, we observe gradually increased δ^13^C and δ^15^N values (Extended Data Fig. 5 and Supplementary Figs. 4.1 and 4.2). This implies that marine foods progressed to constitute the major supply of proteins, as suggested previously based on data from more than 30 Mesolithic humans and dogs, from both coastal and inland sites in Denmark^34,35^. During this period global sea-level rise gradually transformed present-day Denmark into an archipelago, where all human groups had ample access to coastal resources within their annual territories^24^. The local Mesolithic population adapted their diet and culture over time to the changing landscape and our data show that this occurred in a continuous population, without any detectable influx of migrants over a 4,500-year period. Low variability in ^87^Sr/^86^Sr isotope ratios throughout the Mesolithic (Fig. 3 and Supplementary Note 5) could indicate limited long-range mobility and/or deriving dietary sources from more homogeneous environments (for example, marine) than in the succeeding Neolithic periods.

Notably, some of the Danish Mesolithic individuals proved to be closely related^3^. Close kinship is demonstrated in the case of two individuals (NEO568/NEO569), father and son, interred next to each other in the *locus classicus* shell midden site of Ertebølle, and in the case of two individuals (NEO732/NEO733), mother and daughter, that were buried together at Dragsholm. The Ertebølle grave was the first discovered human skeleton in Denmark (excavated in the 1890s) that indisputably represented hunter-gatherers. After the excavation of this site, academic reasoning rooted in Biblical narration about early prehistory in Scandinavia lost momentum. The excavation data cannot reveal whether they were buried simultaneously; it can be ascertained only that the boy (infant, less than two years of age) was positioned less than one metre from his father (the ‘Ertebølle Man’). Excavations at Dragsholm in 1973 uncovered a well-preserved double burial containing a grave with two Mesolithic women as well as a male grave with grave goods suggesting an Early Neolithic date for the latter^36^. A close kin relationship was suggested for the two Dragsholm women on the basis of physical anthropological observations^37^. It was suggested that they were sisters, but this can now be corrected to a co-burial of a mother and daughter. Our data also show that the male in the adjacent burial (‘Dragsholm Man’, NEO962) was not related to the two women. These cases show that close biological kinship was socially relevant to Late Mesolithic groups in Northern Europe and affected the mortuary treatment of dead members of their society.

## Early Neolithic transition

The emergence of the Neolithic FBC in Denmark has occupied a central position in archaeological research and debate throughout the past 175 years^8,38,39^. The defining element of the Neolithic, a food-producing economy based on domesticates of southwest Asian origin, was indisputably present in Denmark from around 5,900 cal. bp^11,38^. The neolithization saw a boom of new shapes and types introduced in Danish material culture, including funnel-shaped beakers and polished flint axes. From about 5,800 cal. bp, monumental long barrows of wood and earth were added to the repertoire, and about 200 years later, burials built of soil, surrounded by raised stones and including stone-built chambers, were erected as dominant landmarks in the farmland^40^. After 5,300 cal. bp, larger and more complex stone-constructed passage graves in large earthen tumuli emerged^41^. Meanwhile, simple, non-monumental burials continued along with the megalithic tombs all through the FBC epoch^42^. Habitation deposits, dating to the earliest centuries of the Neolithic, on top of many Mesolithic Ertebølle coastal shell middens may be interpreted as a local continuation of marine gathering and fishing. By contrast, other settlements with regular long houses on easily farmed soils further inland are associated with remains of domestic plants and animals suggesting a very clear distinction from the previous Mesolithic Ertebølle period^39,43^.

**Extended Data Fig. 6:**
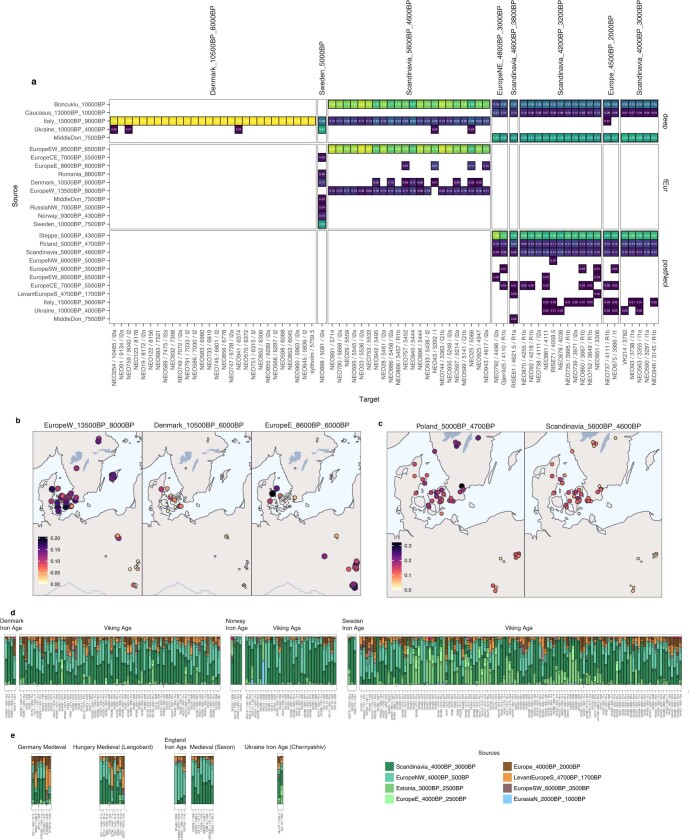
Ancestry modelling of ancient individuals. **a**, Heatmap of ancestry proportions for 72 ancient individuals from Denmark dated to older than 3,000 cal. BP estimated from supervised mixture models. Results for three different sets of ancestry source groups (deep, fEur, postNeol, Supplementary Data IV) are distinguished in facet rows. Genetic cluster membership for Danish target individuals is indicated by column facets. **b**, Spatial distribution of estimated ancestry proportions of three different HG sources for Neolithic farmer individuals from Scandinavia and Poland. **c**, Spatial distribution of estimated ancestry proportions of two different farmer sources for LNBA individuals from Scandinavia and Poland. **d**, Ancestry proportions for Scandinavian Iron Age and Viking Age individuals (postBA reference set). **e**, Ancestry proportions for selected ancient European individuals with ancestry related to Scandinavian LNBA individuals (source Scandinavia_4000BP_3000BP, postBA reference set, Supplementary Data IV).

**Extended Data Fig. 7:**
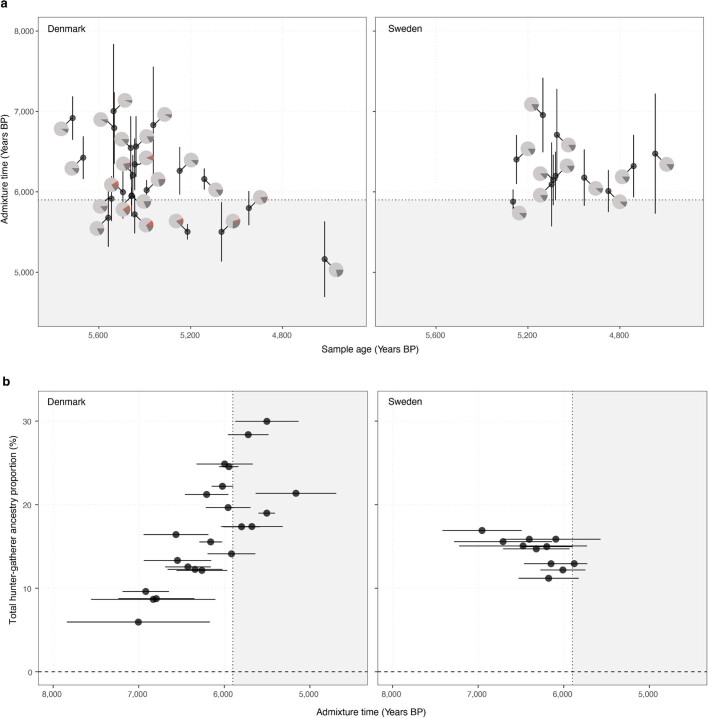
Contrasting hunter-gatherer admixture dynamics in Neolithic farmer individuals from Denmark and Sweden. **a**, Admixture time estimated using DATES^44^ as a function of age for Neolithic farmer individuals from Denmark (left) and Sweden (right). Pie charts indicate ancestry composition (light grey - farmer ancestry; dark grey - non-local hunter-gatherer ancestry; colour - local hunter-gatherer ancestry). **b**, Total amount of hunter-gatherer ancestry proportion as a function of admixture time for Neolithic farmer individuals from Denmark (left) and Sweden (right). Error bars indicate ± 1 standard error of admixture time estimate.

Regardless of these nuances, at around 5,900 cal. bp, our multiproxy dataset documents a marked and abrupt concomitant shift in genetic, phenotypic, dietary and vegetation parameters (Fig. 3). This is robust evidence for demic diffusion, settling a long-standing debate^8,38^. As observed elsewhere in Europe^13–15^, the introduction of farming in Denmark was unequivocally associated with the arrival of people with Anatolian farmer-related ancestry. This resulted in a population replacement with limited genetic contribution from the local hunter-gatherers. The earliest example of this typical Neolithic ancestry in our Danish dataset is observed in a bog skeleton of a female from Viksø Mose (NEO601) dated to 5,896–5,718 cal. bp (95%). In the PCA, all Danish Early Neolithic individuals cluster at the ‘late’ end of the European Neolithic farmer cline and consistently show some of the largest amounts of hunter-gatherer ancestry (10–35%) among all European Neolithic farmer genomes included (Figs. 1 and 3 and Extended Data Figs. 1 and 5a and Supplementary Data 4). In IBD clustering analyses, the Danish individuals form part of a genetic cluster (Scandinavia_5600BP_4600BP) together with FBC-associated individuals from Sweden and Poland, and also show close affinity with Polish individuals from the Globular Amphora culture (GAC) (Extended Data Fig. 4b). This could suggest an eastern European proximate origin of the Early Neolithic farmers in Denmark. Using more proximate ancestry modelling, we find that Neolithic FBC-associated individuals across Denmark, Sweden and Poland derived their hunter-gatherer ancestry component predominantly from a source related to WHG (EuropeW_13500BP_8000BP). Ancestry related to Danish Mesolithic hunter-gatherers (Denmark_10500BP_6000BP) is found in smaller proportions (less than around 10%) and only in a subset of the FBC individuals from Denmark (Extended Data Fig. 6). Moreover, this tends to occur in more recent individuals (dated to around 5,400 cal. BP onwards) who are also showing the overall largest amount of total hunter-gatherer ancestry (for example, NEO945 and NEO886; Fig. 3 and Extended Data Figs. 3 and 6a,b). Using DATES^44^, we found that admixture times for a large proportion of Danish Neolithic individuals predates 5,900 cal. bp when FBC emerged in Denmark, particularly for the earliest individuals (Extended Data Fig. 7). More recent admixture times (post dating the arrival of FBC in Denmark) were mainly observed in individuals dated to after about 5,400 cal. bp, and were associated with overall higher hunter-gatherer proportions. These observations were in marked contrast to FBC-associated individuals from Sweden, where admixture times and hunter-gatherer ancestry did not change over time, and no admixture with local Swedish hunter-gatherers was detected.

Our results demonstrate a population turnover in Denmark at the onset of the neolithisation by incomers who displayed a mix of Anatolian Neolithic farmer ancestry and non-local hunter-gatherer ancestry. Ancestry related to the local Danish hunter-gatherers could be detected only late in the Danish Neolithic gene pool, suggesting gene flow with groups of late surviving hunter-gatherers, as also documented in other European regions (Iron Gates^45^, Central Europe^13^ and Spain^46^). We do not know how the Mesolithic Ertebølle population disappeared. Some may have been isolated in small ‘pockets’ of brief existence and/or adapted to a Neolithic lifestyle. The most recent individual in our Danish dataset with hunter-gatherer ancestry is the aforementioned Dragsholm Man (NEO962), dated to 5,947–5,664 cal. bp (95% confidence interval) and archaeologically assigned to the FBC based on his grave goods^37^. Our data confirm a typical Neolithic diet matching the cultural affinity but contrasting his hunter-gatherer ancestry. He clearly represents a local person of Mesolithic ancestry who lived in the short Mesolithic-Neolithic transition and adopted the culture and diet of the immigrant farmers. A similar case of late hunter-gatherer ancestry in Denmark was observed when analysing human DNA obtained from a piece of chewed birch pitch from the site of Syltholm on Lolland^47^, dated to 5,858–5,661 cal. bp (95%). Thus, individuals with hunter-gatherer ancestry persisted for decades and perhaps centuries after the arrival of farming groups in Denmark, although they have left only a minor genomic imprint on the population of the subsequent centuries. Similar ‘relic’ hunter-gatherer ancestry is also found in the Evensås individual (NEO260) from west-coast Sweden, dated to 5913–5731 cal. bp^3^.

From the onset of the Neolithic in Denmark, diet shifted abruptly to a dominance of terrestrial sources as evidenced by δ^13^C values around −20‰ and δ^15^N values around 10‰ (Fig. 3 and Extended Data Fig. 5). In line with archaeological evidence, these isotopic data show that domesticated crops and animals provided the main supply of proteins from this point onwards. Isotope values remained stable at these levels throughout the following periods, although with somewhat greater variation after about 4,500 cal. bp (Fig. 3). Five Neolithic and Early Bronze Age individuals have δ^13^C and δ^15^N values that indicate a substantial intake of high trophic marine food. This is especially pronounced for the individual NEO898 (Svinninge Vejle), one of two Danish Neolithic individuals displaying ancestry related to Swedish late hunter-gatherers (see below). A considerably higher variability in individual ^87^Sr/^86^Sr values can be seen with the start of the Neolithic. This continues in the later periods (Supplementary Note 5) and is not easily explained by biases in sampling as most of our samples, regardless of ancestry and time period, are concentrated in the more easterly parts of Denmark where bone preservation conditions are generally good (Fig. 1 and Supplementary Fig. 5.3). This pattern could suggest that the Neolithic farmers in Denmark occupied and/or consumed food from more diverse landscapes, or were more mobile than the preceding hunter-gatherers. The Neolithic transition also marks a considerable rise in frequency of major effect alleles associated with light hair pigmentation^48^, whereas predictions throughout the first millennium of the Neolithic (FBC epoch) mostly indicate a lower stature than present day, echoing previous findings^32,49^.

Pitted Ware culture (PWC) originated on the Scandinavian peninsula and the Baltic islands east of the Swedish mainland but emerged around 5,100–4,700 cal. bp in the northern and eastern part of Denmark, where it coexisted with the FBC^50,51^. It is characterized by coarse pottery that is often decorated with pits and subsistence based on a combination of marine species and agricultural products. No burials associated with the PWC have been discovered in Denmark. Of note, however, the genomes of two approximately 5,200-year-old male individuals (NEO33, NEO898) found in Danish wetland deposits proved to be of hunter-gatherer ancestry related to that of PWC individuals from Ajvide on the Baltic island of Gotland (Sweden)^52^ (Figs. 2, 3 and Extended Data Fig. 4a). Of the two individuals, NEO033 (Vittrup, Northern Jutland) also displays an outlier Sr signature (Fig. 3), perhaps suggesting a non-local origin that matches his unusual ancestry. Overall, our results demonstrate direct contact across the sea between Denmark and the Scandinavian peninsula during this period, which is in line with archaeological findings^50,51^.

**Fig. 4:**
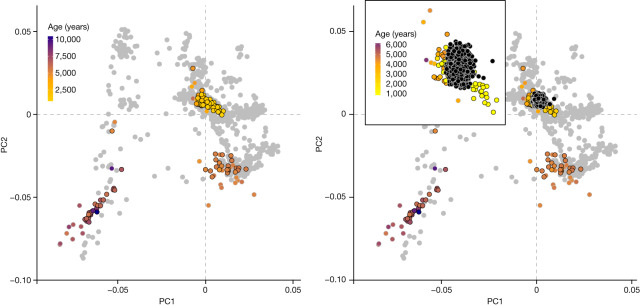
Genetic legacy of ancient Danish individuals. PCA of 2,000 modern Danish genomes from the iPSYCH study^62^ in the context of ancient western Eurasian individuals. Coloured symbols indicate sample age for ancient Danish individuals, whereas grey symbols indicate 1,145 ancient imputed individuals from across Western Eurasia^3^. Modern Danish individuals are indicated by black filled circles and are shown on the right. Inset, a magnified view of the cluster with modern Danes. The colour scale in the inset represents the age range of the ancient samples within the magnified region only.

## Later Neolithic and Bronze Age

Europe was transformed by large-scale migrations from the Pontic–Caspian Steppe around 5,000–4,800 cal. bp. This introduced steppe-related ancestry to most parts of the continent within a 1,000-year span and gave rise to the Corded Ware culture (CWC) complex^1,2^. In Denmark, this coincided with the transition from the FBC to the SGC, the regional manifestation of the CWC complex. The transition to single graves in round tumuli has been characterized archaeologically by two expansion phases: a primary and rapid occupation of central, western and northern Jutland (west Denmark) starting around 4,800 cal. bp and a later and slower expansion across the Eastern Danish Islands starting around 4,600 cal. bp^53,54^. In the eastern parts of the country, SGC traits are less visible, whereas FBC traditions such as burial in megalithic grave chambers persisted^55^. This cultural shift represents another classical archaeological enigma, with explanations favouring immigration versus cultural acculturation competing for generations^19,56^.

Insights from a few low-coverage genomes^1,57^ have indeed shown a link to the Steppe expansions, but by mapping out ancestry components in the 100 ancient genomes we now uncover the full impact of this event and demonstrate a second near-complete population turnover in Denmark within just 1,000 years. This genetic shift was evident from PCA and ADMIXTURE analyses, in which Danish individuals dating to the SGC and Late Neolithic and Bronze Age (LNBA) cluster with other European LNBA individuals and show large proportions of ancestry components associated with Yamnaya groups from the Steppe (Figs. 1 and 3 and Extended Data Fig. 1). We estimate around 60–85% of ancestry related to Steppe groups (Steppe_5000BP_4300BP), with the remainder contributed from individuals with farmer-related ancestry associated with Eastern European GAC (Poland_5000BP_4700BP; 10–23%) and to a lesser extent from local Neolithic Scandinavian farmers (Scandinavia_5600BP_4600BP; 3–18%) (Extended Data Fig. 6a,b). Although the emergence of SGC introduced a major new ancestry component in the Danish gene pool, it was not accompanied by apparent shifts in dietary isotopic ratios, or Sr isotope ratios (Fig. 3). Our complex trait predictions, however, indicate an increase in height (Fig. 3 and Supplementary Note 2), which is consistent with ancient Steppe individuals being predicted taller than average European Neolithic individuals before the steppe migrations^32,49,58^.

Because of poor preservation conditions in most of western Denmark, we do not have skeletons from the earliest phase of the SGC (around 4,800 cal. bp) so we cannot unequivocally demonstrate that these people carried steppe-related ancestry. SGC burial customs were implemented in different ways in the southern and the GAC-related northern parts of the peninsula, respectively^18^ and considering recent genetic results in other regions^59^, it is plausible that differing demographic processes unfolded within Denmark. However, we know that steppe ancestry was present 200 years later in SGC-associated skeletons from the Gjerrild grave^57^. The age of the Gjerrild skeletons (from around 4,600 cal. bp) matches the earliest example of steppe-related ancestry in our current study, identified in a skeleton from a megalithic tomb at Næs (NEO792). We estimated around 85% of Steppe-related ancestry in this individual, the highest amount among all Danish LNBA individuals (Extended Data Fig. 6a). Notably, NEO792 is also contemporaneous with the two most recent individuals in our dataset showing Anatolian farmer-related ancestry without any steppe-related ancestry (NEO580, Klokkehøj and NEO943, Stenderup Hage) testifying to a short period of ancestry co-existence before the FBC disappeared—similar to the disappearance of the Mesolithic Ertebølle people of hunter-gatherer ancestry a thousand years earlier. Using Bayesian modelling we estimate the duration between the first appearance of Anatolian farmer-related ancestry to the first appearance of Steppe-related ancestry in Denmark to be between 876 and 1,100 years (95% prob. interval, Supplementary Note 3) implying that the former type of ancestry was dominant for less than 50 generations.

**Extended Data Fig. 8:**
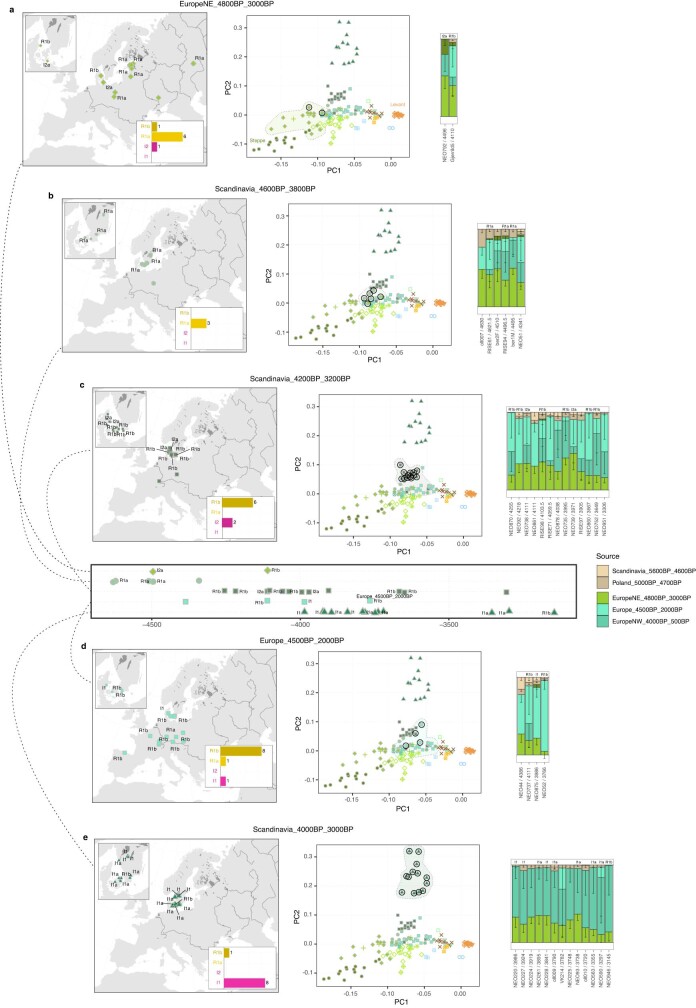
Fine-scale structure in late Neolithic and Early Bronze Age (LNBA) Scandinavians c. 4,500-3,000 cal. BP. **a**–**e**, Geographic locations and PCA based on pairwise IBD sharing (middle) of 148 European LNBA individuals predating 3,000 cal. BP (Supplementary Data IV). Geographic locations are shown for 65 individuals belonging to the five genetic clusters observed in 38 ancient Scandinavians (**a**,**b**, LNBA phase I; **c**,**d**, LNBA phase II; **e**, LNBA phase III; temporal sequence shown in timeline in centre of plot). Individual assignments and frequency distribution of major Y chromosome haplogroups are indicated in maps and timeline. Plot symbols with black circles indicate the 38 Scandinavian individuals in the PCA panels. Ancestry proportions for the 38 Scandinavian individuals estimated using proximal source groups from outside Scandinavia (postNeolScand source set) are shown on the right of the respective cluster results.

The following Late Neolithic ‘Dagger’ epoch (around 4,300–3,700 cal. bp) in Denmark has been described as a time of integration of culturally and genetically distinct groups^54^. Bronze became dominant in the local production of weapons while elegantly surface-flaked daggers in flint were still the dominant male burial gift. Unlike the SGC epoch, this period is richly represented by human skeletal material. Although broad population genomic signatures suggest genetic stability in the LNBA (Figs. 1 and 3), patterns of pairwise IBD-sharing and Y chromosome haplogroup distributions in a temporal transect of 38 LNBA Danish and southern Swedish individuals indicate at least three distinct ancestry phases during this approximately 1,000-year time span (Extended Data Figs. 4c and 8).

LNBA phase I: an early stage between around 4,600 and 4,300 cal. bp, in which Scandinavians cluster with early CWC individuals from Eastern Europe, rich in Steppe-related ancestry and males with an R1a Y chromosomal haplotype (Extended Data Fig. 8a,b). Archaeologically, these individuals are associated with the later stages of the Danish SGC and the Swedish Battle Axe Culture.

LNBA phase II: an intermediate stage largely coinciding with the Dagger epoch (around 4,300–3,700 cal. bp), in which Danish individuals cluster with central and western European LNBA individuals dominated by males with distinct sub-lineages of R1b-L51^3^ (Extended Data Fig. 8c,d). Among them are individuals from Borreby (NEO735, 737) and Madesø (NEO752).

LNBA phase III: a final stage from around 4,000 cal. bp onwards, in which a distinct cluster of Scandinavian individuals dominated by males with I1 Y-haplogroups appears (Extended Data Fig. 8e). Y chromosome haplogroup I1 is one of the dominant haplogroups in present-day Scandinavians, and we here document its earliest occurrence in an approximately 4,000-year-old individual from Falköping in southern Sweden (NEO220). The rapid increase in frequency of this haplogroup and associated genome-wide ancestry coincides with increase in human mobility seen in Swedish Sr isotope data, suggesting an influx of people from eastern or northeastern regions of Scandinavia, and the emergence of stone cist burials in Southern Sweden^60^, which were also introduced in eastern Denmark during that period^54,61^.

Using genomes from LNBA phase III (Scandinavia_4000BP_3000BP) in supervised ancestry modelling, we find that they form the predominant ancestry source for later Iron and Viking Age Scandinavians (Extended Data Fig. 6d) and other ancient European groups with a documented Scandinavian or Germanic association (for example, Anglo-Saxons and Goths; Extended Data Fig. 6e). When projecting 2,000 modern Danish genomes^62^ on a PCA of ancient Eurasians, the modern individuals occupy an intermediate space on a cline between the LNBA and Viking Age individuals (Fig. 4). This result shows that the foundation for the present-day gene pool was already in place in LNBA groups 3,000 years ago, but the genetic structure of the Danish population was continually reshaped during succeeding millenia.

## Environmental impact

The two documented major population turnovers were accompanied by substantial changes in land use, as apparent from the high-resolution pollen diagram from Lake Højby in Northwest Zealand (Fig. 3) reconstructed using the landscape-reconstruction algorithm (LRA; Supplementary Note 6). We uncovered a direct synchronic link between shifts in a populations’ ancestry profile and land use. During the Mesolithic, the landscape was dominated by primary forest trees (*Tilia*, *Ulmus*, *Quercus*, *Fraxinus*, *Alnus* and so on). At the onset of the Neolithic, the primary forest diminished, cleared by FBC farmers. A new type of forest with more secondary and early successional trees (*Betula* and then *Corylus*) appeared, whereas the proportion between forest and open land remained almost unaltered. From about 5,650 cal. bp deforestation intensified, resulting in an open grassland-dominated landscape. This open phase was short-lived, and the secondary forest expanded again from around 5,500 to 5,000 cal. bp, until another episode of forest clearance occurred during the last part of the FBC epoch. We conclude that the agricultural practice during the FBC was characterized by repeated clearing of the forest followed by regrowth. After about 4,600 cal bp, this strategy changed with the emergence of the SGC and the arrival of Steppe-related ancestry in Denmark. In Western Denmark (Jutland), the arrival of the SGC was characterized by permanent large-scale opening of the landscape to create pastureland^63,64^ and we observe here a similar increase in grassland and cropland at Højby Sø in Eastern Denmark around 4,600 cal. bp (Fig. 3). Notably, this was accompanied by an increase in primary forest cover, especially *Tilia* and *Ulmus*, probably reflecting a development of a more permanent division of the landscape into open grazing areas and primary forests.

## Drivers of change

We have demonstrated examples of both cultural and demic diffusion during the Mesolithic and Neolithic periods in Denmark. Shifts in the Mesolithic material culture appeared without any detectable levels of changes in ancestry, whereas the two cultural shifts in the Neolithic period were clearly driven by new people coming in. Accordingly, groupings of artefacts and monuments into archaeological cultures do not always represent genetically distinct populations and the underlying mechanisms responsible for prehistoric cultural shifts must be examined on a case-by-case basis.

It remains a mystery why the Neolithic farming expansion came to a 1,000-year standstill before entering Southern Scandinavia. It may be that it was complicated by a high Mesolithic hunter-gatherer population density owing to a very productive marine and coastal environment^20,65^. Further, the Danish Ertebølle population may have been acquainted with armed conflict^11,66^ enabling territorial defence against intruders. Alternatively, it has been argued that changing climatic conditions around 6,000 cal. bp became a driver since it enhanced the potential for farming further north^67^, but other studies have not confirmed this^68^. The second population turnover in the late Neolithic resulted in a short period of three competing cultural complexes in Denmark, namely the FBC, the PWC and the SGC. The latter introduced the steppe-related ancestry which has prevailed to this day. There is archaeological evidence that this was a violent time, both in Denmark^69^ and elsewhere^70,71^. Additionally, ancient DNA evidence has demonstrated that plague was widespread during this period^72,73^. In tandem with other indicators of population declines^74^, and widespread reforestation after 5,000 cal. bp^75^, it suggests that the local populations of Central and Northern Europe may have been severely impacted prior to the arrival of newcomers with Steppe-related ancestry. This could explain the rapid population turnover and limited admixture with locals we observe.

While the two major shifts in Danish Mesolithic and Neolithic material culture may have had different drivers and causes, the outcomes were ultimately the same: new people arrived and rapidly took over the territory. With this arrival, the local landscape was modified to fit the lifestyle and culture of the immigrants. This is the hallmark of the Anthropocene, observed here in high resolution in prehistoric Denmark.

## Methods

### Ancient genomic analyses

The 100 ancient Danish genomes analysed here contribute to the 317 shotgun-sequenced genomes in Allentoft et al.^3^. All details concerning sampling, DNA extraction, library preparation, sequencing, basic bioinformatics, authentication and dataset construction are found in ref. ^3^ together with all site descriptions and sample metadata. A condensed list of metainformation on the 100 Danish individuals is released here (Supplementary Data 1) together with a text summarizing the study sites and skeletons (Supplementary Note 1). In brief, laboratory work was carried out in dedicated ancient DNA cleanlab facilities (University of Copenhagen) using optimized ancient DNA methods^1,77^. Double-stranded blunt-end libraries were sequenced (80 bp and 100 bp single-end reads) on Illumina HiSeq 2500 and 4000 platforms. Initial shallow shotgun screening was used to identify samples with sufficient DNA preservation for deeper genomic sequencing. Of the 100 Danish samples that qualified for this, 65 were from tooth cementum, 29 were petrous bones, and 6 were obtained from other bones (Supplementary Data 1). Sequence reads were bioinformatically mapped to the human reference genome (build 37), filtered and merged to sample level followed by estimates of genomic overage, post-mortem DNA damage, contamination, and genetic sex ID (see^3^). For these 100 samples we observed C-to-T deamination fractions ranging from 12.2% to 66.7%, with an average of 34.9% across all samples (Supplementary Data 1), consistent with highly degraded ancient DNA. We genetically identified 67 males, 32 females and one undetermined in our dataset (Supplementary Data 1).

We utilized a new computational method optimized for low-coverage data^21^, to impute genotypes based on genotype likelihoods of ancient individuals with the samtools/bcftools pipeline, and using the 1000 Genomes phased data^78^ as a reference panel. To generate the main dataset in^3^ this was jointly applied to 1,664 shotgun-sequenced ancient genomes, including our 100 ancient Danish genomes, and resulted in a dataset of 8.5 million common SNPs (>1% minor allele frequency and imputation info score > 0.5) for imputed diploid ancient genomes. After removing genomes with low coverage (<0.1X), low imputation quality (average genotype probability <0.98), contamination estimates >5%, or close relatives (first or second degree, lowest coverage relative removed), 67 of the 100 Danish genomes were retained as imputed in downstream analyses. The remaining 33 genomes were analysed as pseudo-haploid genotypes.

For population genetic analyses, we combined ancient samples with two different modern reference panels:

- ‘1000 G’ dataset: whole-genome sequencing data of 2,504 individuals from 26 world-wide populations from the 1000 Genomes project, with genotypes at 7,321,965 autosomal SNPs.
- ‘HO’ dataset: SNP array data of 2,180 modern individuals from 213 world-wide populations, with genotypes at 535,880 autosomal SNPs.

Analyses were based on the 1000 G dataset unless otherwise noted. Individuals not passing imputation quality control cutoffs mentioned above were included in PCA and ADMIXTURE analyses as pseudo-haploid genotypes. Four Danish individuals showed possible signs of DNA contamination (Fig. 3 and Supplementary Data 1) and were excluded from most analyses. To take full advantage of the extensive multiproxy data they were, however, included in Fig. 3. Individual metadata for all genetic analyses related to the ancient Danish individuals as well as selected subset of relevant West Eurasian individuals, are reported in Supplementary Data 3.

For PCA combining ancient and modern Western Eurasians (Fig. 1b), we used the data and framework from^3^ to capture West Eurasian genetic diversity based on n = 983 modern genomes and *n* = 1,105 ancient genomes (HO dataset). Data from a total of 179 ancient Danish genomes are shown in Fig. 1b of which 83 are previously published^1,47,57,76^ (Supplementary Data 3)—the latter being primarily from the Bronze Age and Viking periods. To perform PCA projection for low-coverage individuals, we used smartpca with options ‘lsqproject: YES’ and ‘autoshrink: YES’.

The ADMIXTURE results presented in this study represent subsets of individuals from the full ADMIXTURE runs in^3^ where 1,593 ancient individuals were analysed (*n* = 1,492 imputed, *n* = 101 pseudo-haploid, *n* = 71 excluded as close relatives or with a contamination estimate >5%; HO dataset). Figure 1c represents 176 ancient Danish genomes after excluding three close relatives (Supplementary Data 1 and 4).

D-statistics were obtained using pseudo-haploid genotypes at transversion SNPs in the 1000 G dataset, grouping the non-Danish individuals into populations using their membership in the genetic clusters inferred from IBD sharing (Supplementary Data IV). We computed D-statistics from genotypes in PLINK format using the qpdstat function implemented in the ADMIXTOOLS 2 R package^79^.

Analysis of IBD sharing and mixture models were carried out as described^3^, using the same set of inferred genetic clusters (see Supplementary Data 4). In brief, we used IBDseq^80^ to detect IBD segments, a carried out genetic clustering of the individuals using hierarchical community detection on a network of pairwise IBD-sharing similarities. IBD-based PCA was carried out in R using the eigen function on a covariance matrix of pairwise IBD sharing between the respective ancient individuals. We estimated ancestry proportion in supervised modelling of target individuals as mixtures of different sets of putative source groups via non-negative least squares on relative IBD-sharing rate vectors.

Admixture time inference for FBC-associated individuals was carried out using the linkage-disequilibrium-based method DATES^44^ (HO dataset). We estimated admixture time separately for each target individual from Denmark and Sweden, using hunter-gatherer individuals (*n* = 58) and early farmer individuals (*n* = 49) as the two source groups.

For the PCAs presented in Fig. 4 including modern Danish samples we projected 2,000 imputed samples^81^ of individuals born in 1981–2005 from the iPSYCH2012 case-cohort study^62^ onto the PCA space spanned by the 1,145 non-low coverage or related european and western Asian ancient imputed samples^3^. Otherwise, the analysis is identical to the one described above. The modern individuals were selected from a subset of the random population subcohort component of iPSYCH2012 having all four grandparents born in Denmark, and being of Danish or European ancestry as determined in a separate already existing PCA of main modern-day ancestry groups^81^. This was done using Eigensoft 7.2.1 on the intersect of imputed SNPs from the ancient and modern samples, filtered by minor allele frequency 0.05, pruned using PLINK v1.90b6.21^82^ based on source samples (parameters: –indep-pairwise 1000 50 0.25) leaving 146,895 variants.

The genetic predictions of eye and hair colour were done based on the HIrisPlex system^83^. We used imputed effect allele dosages of 18 out of 24 main effect HIrisPlex variants, available for the ancient samples, to derive probabilities for brown, blue and grey/intermediate eye colour and blond, brown, black and red hair colour, following HIrisPlex formulas (see further details in Supplementary Note 2). We predicted relative ‘genetic height’ using allelic effect estimates from 310 common autosomal SNPs with robustly genome-wide significant allelic effects (*P* < 10^−15^) in a recent GWAS of height in the UK Biobank^84^. Per-sample height polygenic score (PGS) was calculated for ancient individuals as well as 3,467 Danish ancestry male conscripts from the random population subcohort of the iPSYCH2012 case-cohort study^62^ by summing allelic effect multiplied with the effect allele imputed dosage^81^ across the 310 loci. For further details see Supplementary Note 2. Only a fraction of the 100 Danish skeletons were suitable for stature estimation by actual measurement, which is why these values are not reported here.

### Radiocarbon dates and Bayesian modelling of ancestry chronology

For the 100 sample ages in this study we use midpoint estimates of the calibrated and reservoir corrected probability distribution of the radiocarbon age (Supplementary Data 1; further ^14^C dates, associated isotopic measurements, calibrations and reservoir corrections are accessible in ref. ^3^). Focusing on estimating the interval between the two major population turnovers, we established a precise chronology using 81 radiocarbon dates from 64 Danish sites relevant to this particular interval (Supplementary Note 3). A Bayesian approach applied to the radiocarbon dates unifies radiocarbon results, ancestry information, and the high precision curve into one calibration process, thereby gaining greater precision. All models and data calibrations were performed using OxCal v4.4^85–88^ and the calibration dataset from Reimer et al.^89^. We used a trapezoidal phase prior^90,91^ for the calculation of the transitional time interval to determine duration between the first appearance of Anatolian farmer-related ancestry to the first appearance of Steppe-related ancestry in Denmark. We corrected the reservoir effect on bones with significantly increased isotope values (δ^13^C, −18.00 and δ^15^N, +12.00) directly in the models using previously defined reservoir ages as input and calculated the diet reconstruction estimates for the individual in ^14^C years based on the collagen isotope values (Supplementary Note 3 and Supplementary Figs. 3.1 and 3.2); for a similar method see refs. ^92,93^. For combining radiocarbon dates related to the same individual we used the R_Combine() function.

### Stable isotope proxies for diet and mobility

Bulk collagen isotope values of carbon (δ^13^C) and nitrogen (δ^15^N) represent protein sources consumed over several years before death, depending on the skeletal part and the age at death of the individual^94,95^. Generally, δ^13^C values inform on the proportion of marine versus terrestrial protein, whereas δ^15^N values reflect the trophic level from which the proteins were acquired^96,97^. See Supplementary Note 4 for further discussion. Stable isotope values were measured in collagen from all 100 skeletons and the full assemblage of isotopic measurements is available in Supplementary Data 2, and further discussed in Supplementary Note 4. Most of the δ^13^C and δ^15^N measurements were conducted at the ^14^C Centre, University of Belfast according to standard protocols^98^, based on a modified Longin method including ultra-filtration^98,99^. Measured uncertainty was within the generally accepted range of ±0.2‰ (1 s.d.) and all samples were within the acceptable atomic C:N range of 2.9–3.6, showing low likelihood of diagenesis^100,101^.

Strontium isotope analyses can provide a proxy for individual mobility^102–104^. The ^87^Sr/^86^Sr ratio in specific skeletal elements may reflect the local geological signature obtained through diet by the individual during early childhood and it will usually remain unchanged during life and after death^105^. Ongoing controversies exist over the exact use of geographically-defined baseline values^106,107^, which is why we restrict our observations and interpretations of Sr variation to patterns that are only relative to our own data. Measurements of ^87^Sr/^86^Sr ratios in teeth and petrous bones were conducted at the Geochronology and Isotope Geochemistry Laboratory (Department of Geological Sciences, University of North Carolina- Chapel Hill) and data are found in Supplementary Data 2. For further details see Supplementary Note 5.

### Vegetation modelling

Using a high-resolution pollen diagram from Lake Højby, Northwest Zealand^108^, we reconstruct the changes in vegetation cover during the period 5,000–2,400 cal. bc using the landscape-reconstruction algorithm (LRA^109,110^). Although the LRA has previously been applied at low temporal resolution regional scale (fer example, in refs. ^111,112^.), and to Iron Age (and later) pollen diagrams^113,114^, to our knowledge, this is the first time that this quantitative method is applied at local scale to a pollen record spanning the Mesolithic and Neolithic periods in Denmark. In total 60 pollen samples between 6,900 and 4,400 cal. bp were included and the temporal resolution between samples is approximately 40 years. Regional vegetation was estimated with the model REVEALS^109^ based on pollen data from six other lakes on Zealand (see Supplementary Fig. 6.1). From this, regional pollen rain is calculated and local scale vegetation around Højby Sø calculated using the LOVE model^110^. Average pollen productivity estimates for Europe^115^ for 25 wind pollinated species were applied. The reconstructed cover for plant species were then combined into four land cover categories, crops (only cereals), grassland (all other herbs), secondary forest (*Betula* and *Corylus*) and primary forest (all other trees). The vegetation reconstruction from Højby Sø is used to illustrate the vegetation development at the Mesolithic/Neolithic transition in eastern Denmark. For more details see Supplementary Note 6.

### Reporting summary

Further information on research design is available in the Nature Portfolio Reporting Summary linked to this article.

## Online content

Any methods, additional references, Nature Portfolio reporting summaries, source data, extended data, supplementary information, acknowledgements, peer review information; details of author contributions and competing interests; and statements of data and code availability are available at 10.1038/s41586-023-06862-3.

### Supplementary information

Supplementary Information Supplementary Notes 1–6: **1**, Overview of Danish Samples (including Figs S1.1 to S1.3); **2**, Polygenic prediction of height, eye colour and hair colour (including Table S2.1); **3**, Bayesian Chronological models of the transition (including Figs S3.1 to S3.6); **4**, Dietary variation in Mesolithic, Neolithic and Bronze Age Denmark (including Figs S4.1 to S4.2); **5**, Strontium Analysis of Danish Samples (including Figs S5.1 to S5.3, and Table S5.1); and **6**, Vegetation and landscape in Post-Glacia Denmark – illustrated using a high-resolution land cover reconstruction (LOVE) from Lake Højby, Northwest Zealand (including Figs S6.1 to S6.2).

Reporting Summary

Supplementary Data 1 Basic overview of samples and genetic data.

Supplementary Data 2 Isotopic data from 100 Danish samples.

Supplementary Data 3 Isotopic data from 100 Danish samples: **a**, Metadata for ancient genomes from Denmark used in this study; **b**, Metadata for selected contextual ancient genomes from Western Eurasia.

Supplementary Data 4 Ancestry proportions for sample sets: **a**, Ancestry proportions for set “deep”; **b**, Ancestry proportions for set “fEur”; **c**, Ancestry proportions for set “postNeol”; **d**, Ancestry proportions for set “postBA”; **e**, Ancestry proportions for set “postNeolScand”.

## Data Availability

Sequencing data analysed in this study is released in the accompanying study ‘Population genomics of post-glacial western Eurasia’^3^. These are publicly available on the European Nucleotide Archive under accession PRJEB64656, together with sequence alignment map files, aligned using human build GRCh37. The full analysis dataset including both imputed and pseudo-haploid genotypes for all ancient individuals used in this study is available at 10.17894/ucph.d71a6a5a-8107-4fd9-9440-bdafdfe81455. Aggregated IBD-sharing data as well as hi-resolution versions of supplementary figures are available at Zenodo under accession 10.5281/zenodo.8196989. Maps were created in R using public domain Natural Earth map data.
