# Supplementary material for: 100 ancient genomes show repeated population turnovers in Neolithic Denmark
Source: Nature. 2024 Jan 10;625(7994):329–37. doi: 10.1038/s41586-023-06862-3 (PMC10781617; doi:10.1038/s41586-023-06862-3)
Supplement: Supplementary file 1 — Supplementary Notes 1–6: 1, Overview of Danish Samples (including Figs S1.1 to S1.3); 2, Polygenic prediction of height, eye colour and hair colour (including Table S2.1); 3, Bayesian Chronological models of the transition (including Figs S3.1 to S3.6); 4, Dietary variation in Mesolithic, Neolithic and Bronze Age Denmark (including Figs S4.1 to S4.2); 5, Strontium Analysis of Danish Samples (including Figs S5.1 to S5.3, and Table S5.1); and 6, Vegetation and landscape in Post-Glacia Denmark – illustrated using a high-resolution land cover reconstruction (LOVE) from Lake Højby, Northwest Zealand (including Figs S6.1 to S6.2). [file 41586_2023_6862_MOESM1_ESM.docx]

100 ancient genomes show two rapid population turnovers in Late Stone Age Denmark:

**Supplementary Notes 1 to 6**

**Contents:**

[**1) Overview of Danish samples 3**](#_o8m1oapfm4pe)

[Introduction 3](#_cq6c7hzgq9zn)

[Geographic distribution 4](#_s0yjmxi8f9rz)

[Sample environments 7](#_k3ymw5v8kauo)

[References 9](#_z0rg3t3o90bq)

[**2) Polygenic prediction for height, eye colour and hair colour 11**](#_l2mhvdgx5xhg)

[References 16](#_gugvg8h40m51)

[**3) Bayesian chronological models of the transition 18**](#_xmokfpx78ic0)

[Introduction 18](#_rgh7l9hg6y7e)

[Materials 19](#_8uc3mc1b0zg4)

[OxCal Model Details and Outputs 20](#_ti4nt826o8gg)

[Estimating the Transition Phase and span of Neolithic farmer ancestry 20](#_3iwku7nbmk0l)

[Results 21](#_xgxutjqki3vc)

[**4) Dietary variation in Mesolithic, Neolithic and Bronze Age Denmark 32**](#_h61hurtx5dki)

[Introduction 32](#_92nj6km600eq)

[Material and methods 32](#_c9s54rm8myhw)

[Dietary variation versus cultural and genetic history 34](#_68d93balli5)

[Mesolithic food composition 36](#_5u0j8v5pp1dh)

[Neolithic and Bronze Age food composition 37](#_680umi4dt4e0)

[References 38](#_44sz52saxunn)

[**5) Strontium analysis of Danish samples 46**](#_iuad13ltfqji)

[Introduction 46](#_pu0kf34rkd4h)

[Results 48](#_92gehbyxo706)

[References 52](#_vx3wl3782mva)

[**6) Vegetation and landscape in Post-Glacial Denmark – illustrated using a high-resolution land cover reconstruction (LOVE) from Lake Højby, Northwest Zealand 55**](#_a1097suf5xb9)

[The Mesolithic-Neolithic transition: palynological evidence 55](#_wl5ga2l51arz)

[Mesolithic 57](#_fpgv97xgs57g)

[Early Funnel Beaker Culture (FBC) 3900-3500 cal. BC 60](#_799zqmne4167)

[Middle FBC 3500-3200 cal. BC 61](#_hvt8odls6ht2)

[Late FBC 3200-2800 cal. BC 62](#_l9q1b6bbsfq)

[SGC 2800-2400 cal. BC 62](#_yfaw4xrdj0cq)

# 1) Overview of Danish samples

Anders Fischer^1,2,3,4^

^1^Department of historical studies, Gothenburg university, Sweden

^2^Cluster of Excellence ROOTS, Kiel University, Leibnizstr. 3, 24118 Kiel

^3^Sealand Archaeology, Gl. Røsnæsvej 27, 4400 Kalundborg, Denmark
^4^Lundbeck Foundation GeoGenetics Centre, GLOBE Institute, University of Copenhagen

### Introduction

For generations prehistorians have debated if the causes of major changes observed in the archaeological record were the result of cultural diffusion or immigration. One of the most prominent of these debates has been centred at the transition to a Neolithic economy and cultural complex in Denmark^1^. To shed light on this topic, we undertook an intensive palaeogenomic analysis of relevant Danish archaeological remains. Additionally, we studied concomitant changes in diet, lifeways and land use, through stable isotopes, palaeoenvironmental data, and other archaeological information. The present study centres on the analysis of 100 ancient Danish genomes, which are an integral part of the larger study of 317 western Eurasian shotgun-sequenced genomes presented in Allentoft et al.^2^. All details concerning sampling, DNA extraction, library preparation, sequencing, and authentication are found in the above study together with all site descriptions and sample metadata. A condensed list of meta information on the 100 Danish samples is, however, provided here as Supplementary Data I. A descriptive summary of the Danish material analysed is provided here.

Denmark has an unusually large and varied assemblage of Mesolithic and Neolithic human skeletal remains, resulting from a long history of archaeological research, in addition to environmental conditions amenable to the preservation of ancient bones. The samples in this study (Supplementary Data I) were collected with the primary focus of obtaining skeletal remains from the Mesolithic and the Early Neolithic, and reflecting a broad spectrum of chronological, geographic, and social diversity. Additionally, a minor proportion of skeletal remains belonging to the Middle and Late Neolithic and early Bronze Age (defined following well-established South Scandinavian archaeological terminology) were analysed for this project to provide additional contextualising data points.

### Geographic distribution

Our sampling involved skeletal remains of 197 ancient Danish humans (data not shown), and following an initial shallow shotgun sequencing screening phase, 100 of these displayed DNA preservation of acceptable quality. The find locations (and site names) of these 100 are shown in Figs. S1.1 and S1.2, and Supplementary Data 1. The uneven geographical distribution across Denmark reflects geology as well as museum and excavation activity. Early prehistoric bones exhibit very poor preservation (if preserved at all) in the lime deficient soils of Western Jutland (Figs. S1.1, S1.2, S1.3), resulting in a complete lack of samples from this region. The material represent excavations dating to 1838 onwards - nearly since the birth of archaeology as a scientific discipline^1^. During much of that time the National Museum in cooperation with the Zoological Museum, both in Copenhagen, were the major operators in collecting Danish prehistoric human bones. University of Copenhagen also received ancient physical anthropological finds and this collection developed into the Anthropological Laboratory, which nowadays curates the majority of prehistoric human skeletal materials from Denmark. Although these institutions maintained field activities and contacts all over the country ^(e.g.^[^3^](https://paperpile.com/c/JT7E3Y/Y8jbz)^)^, find sites close to Copenhagen are overrepresented in their collections – and consequently potentially also in the present study (Figs. S1.1, S1.2). This study has greatly benefitted from hospitable and long-lasting access to the collections of all three institutions as well as generous access to regional culture-historical museums (Fig. S1.3) with especially large collections of prehistoric human skeletal material, located geographically widespread in Hjørring, Rudkøbing and Kalundborg.


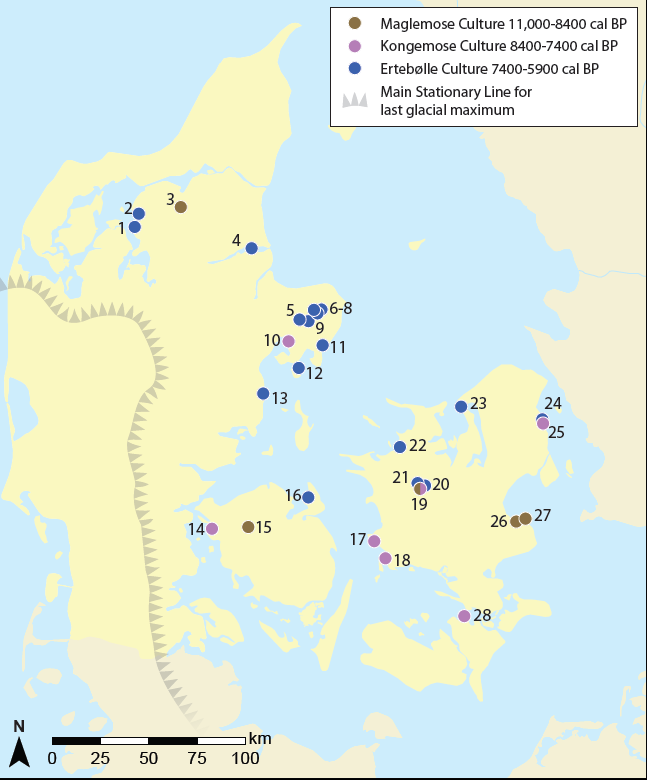


**Figure S1.1. Mesolithic samples.** Find locations of 38 Mesolithic individuals from Denmark. All three major chronological groups (Maglemose, Kongemose, Ertebølle) have been sampled intensely. The sites in question are: 1 Ertebølle (*locus classicus*), 2 Bjørnsholm, 3 Hedegaard, 4 Havnø, 5 Koed, 6 Nederst, 7-9 Fannerup D, E and F, 10 Rønsten, 11 Holmegård-Djursland, 12 Vængesø II, 13 Norsminde, 14 Tybrind Vig, 15 Koelbjerg, 16 Langø Skaldynge, 17 Korsør Nor, 18 Tudse Hage, 19 Bodal K, 20 Ravnsbjerggård II, 21 Kongemose (*locus classicus*), 22 Dragsholm, 23 Sølager, 24 Henriksholm-Bøgebakken, 25 Vedbæk Boldbaner, 26 Strøby Grøftemark, 27 Køge Sønakke, 28 Orehoved Sejlrende. Map produced by K-G. Sjögren, Rich Potter and Anders Fischer.

**
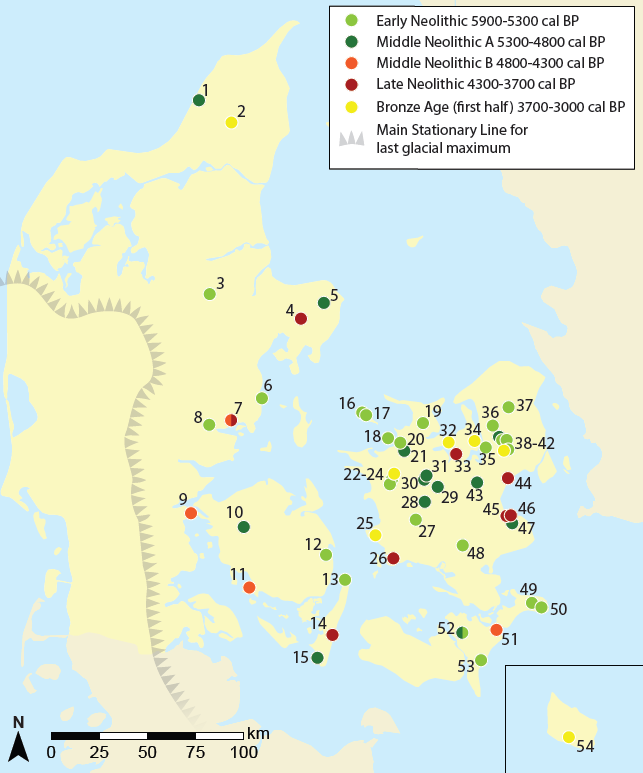
**

**Figure S1.2.** **Neolithic and Bronze Age samples.** Find locations of 55 Neolithic and 7 Bronze Age individuals from Denmark. The sites in question are: 1 Vittrup, 2 Klæstrupholm Mose, 3 Læsten Mose, 4 Kolind, 5 Kainsbakke, 6 Rude, 7 Toftum Mose, 8 Bygholm Nørremark, 9 Stenderup Hage, 10 Neverkær Mose, 11 Klokkehøj, 12 Sludegård Sømose, 13 Lohals, 14 Gammellung, 15 Myrebjerg Mose, 16 Rødhals, 17 Sejerby, 18 Pandebjerg, 19 Vig Femhøve, 20 Dragsholm, 21 Svinninge Vejle, 22 Madesø, 23 Jorløse Mose, 24 Tissøe, 25 Magleø, 26 Borreby, 27 Grøfte, 28 Døjringe, 29 Vanløse Mose, 30 Storelyng Eel Picker, 31 Storelyng Fire Lighter, 32 Bybjerg, 33 Kyndeløse, 34 Lollikhuse, 35 Roskilde Fjord, 36 Jørlundegård, 37 Salpetermosen, 38 Sigersdal Mose, 39 Viksø Mose, 40 Sigersdal, 41 Hove Å, 42 Tysmose, 43 Vibygårds Mose, 44 Mosede Mose, 45 Barhøj, 46 Strøby Ladeplads, 47 Avlebjerg, 48 Porsmose, 49 Dalmosegård, 50 Mandemarke, 51 Næs, 52 Lundby-Falster, 53 Elkenøre, 54 Vasagård. Map produced by K-G. Sjögren, Rich Potter and Anders Fischer.


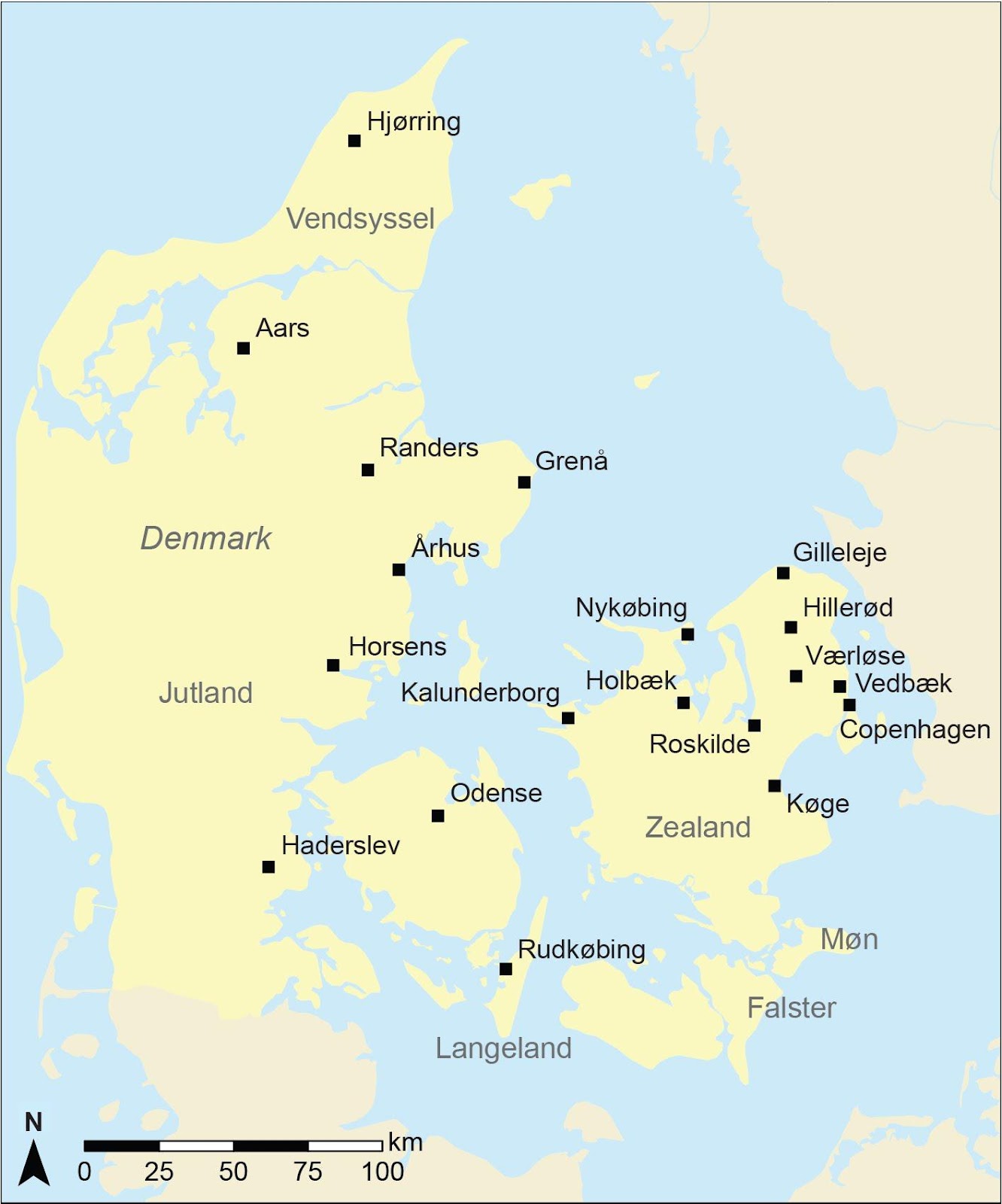


**Figure S1.3.** **Sampled museum collections.** Map produced by Rich Potter.

### Sample environments

Our Danish samples basically derive from three find categories: burials, bog depositions, and stray finds of single skeletal elements.

*Burials*

Nearly all burials known from the Danish Mesolithic are inhumation graves and this category is also richly represented throughout the subsequent Neolithic periods. Monumental architecture did not appear in Denmark (and adjacent regions) until the earthen long barrows of the Early Neolithic Funnel Beaker Culture (FBC)[^4^](https://paperpile.com/c/JT7E3Y/osMAI). These rectangular or trapezoid earth mounts cover one or more burials, built of planks and/or small stones. The nature of their construction is not ideal for bone preservation. Consequently, relatively few individuals in our study derive from such contexts. Somewhat better preservation conditions are found in the dolmens, which are monumental burial constructions of earth and large, raised stones (megaliths). Although their burial chambers are relatively small, they often include several skeletons interred consecutively, and with the most recently buried individual often dating to the Late Neolithic or Early Bronze Age. During the FBC epoch the construction of megalithic burial chambers developed into architecturally more complex and often significantly larger passage graves. Their chambers can be as long as 14 m, and there are examples of original roof-heights up to c. 2.5 m [^5^](https://paperpile.com/c/JT7E3Y/YsLFr). Skeletons from these originally water-proof constructions with a thick soil cover often show great preservation. Such preservation conditions have never been observed in the low tumuli, typically of sandy sediment, of the Single Grave Culture (SGC) in the western part of the country. At best a dark ‘shade’ of the skeleton is all there is left. During the Late Neolithic, the emergence of large collective burial cists (covered in lime-rich soil in eastern parts of the country)[^6^](https://paperpile.com/c/JT7E3Y/Gv6mz/?locator=27) represent conditions that are generally better for the preservation of human bones.

*Depositions in watery environments*

In this study we also included a number of skeletal remains without documented archaeological context, found during peat digging. These were AMS dated (Supplementary Datas I and II) and sampled for DNA. There are hundreds of such bog skeletons in Danish museums but we focused on sampling individuals from Jutland and the islands south of Zealand – areas which had a relative paucity of Neolithic material. These skeletons typically represent deliberate depositions of whole bodies in watery environments distant from coeval habitation[^7–9, cf. 10^](https://paperpile.com/c/JT7E3Y/8RQay+NFE8x+CmCd9+FtrxP/?prefix=,,,cf.). From the content and internal organisation of the best-examined bog skeleton sites it appears they are results of sacrificial deposition. A couple of these individuals were found with ropes around their necks[^7^](https://paperpile.com/c/JT7E3Y/8RQay), and several reveal traces of fatal physical violence associated with their deposition[^e.g. 11^](https://paperpile.com/c/JT7E3Y/yPiMd/?prefix=e.g.). The tradition of sacrificial deposition of human bodies - and items of symbolic importance - in the watery elements seems also to have taken place in fjords and open sea (NEO891 Roskilde Fjord; NEO943 Stenderup Hage; NEO898 Svinninge Vejle). The majority of the previously undated bog skeletons turned up to be of Early Neolithic date, suggesting a high frequency of human sacrifice in wetlands during this time. The remainder were spread throughout subsequent periods, some of which were irrelevant for the current study (i.e. Iron Age^2^).

*Isolated bones and teeth*

We have also sampled a number of isolated human bones. These are frequently found within layers of settlement debris at Mesolithic sites[^12^](https://paperpile.com/c/JT7E3Y/oOpaM). Likewise, disarticulated human bones are sometimes seen in Neolithic enclosure ditches of ceremonial nature[^13^](https://paperpile.com/c/JT7E3Y/2dRyh). A fragment of a human jaw (NEO19) from the submerged habitation site of Rønsten exemplifies this category of finds. It carries cut marks from a flint knife revealing a deliberate dismembering of the body – be it for venerational and/or cannibalistic reasons[^14^](https://paperpile.com/c/JT7E3Y/VQWlY).

### References

1. [Fischer, A. & Kristiansen, K. *The Neolithisation of Denmark. 150 years of debate*. (J.R. Collis Publications. Sheffield, 2002).](http://paperpile.com/b/JT7E3Y/rB2Qq)

2. [Allentoft, M. E. *et al.* Population Genomics of Stone Age Eurasia. *bioRxiv* 2022.05.04.490594 (2022) doi:](http://paperpile.com/b/JT7E3Y/hXZgD)[10.1101/2022.05.04.490594](http://dx.doi.org/10.1101/2022.05.04.490594)[.](http://paperpile.com/b/JT7E3Y/hXZgD)

3. [Madsen, A. P. *et al.* Affaldsdynger fra Stenalderen i Danmark undersøgte for Nationalmuseet (French summary). (Reitzel, Copenhagen, 1900)[Excerpts reprinted in^1^].](http://paperpile.com/b/JT7E3Y/Y8jbz)

4. [Sjögren, K. G. & Fischer, A. The chronology of Danish dolmens.](http://paperpile.com/b/JT7E3Y/osMAI) Results from 14C dates of human bones. Journal of Neolithic Archaeology, 14 March 2023 doi 10.12766/jna.2023.1 CC-BY 4.0.

5. [Hansen, S. I. *Jættestuebyggerne: Arkitektur i Danmarks stenalder*. (Kahrius, 2016).](http://paperpile.com/b/JT7E3Y/YsLFr)

6. [Bröste, K., Jørgensen, J. B., Becker, C. J. & Brøndsted, J. *Prehistoric man in Denmark: A study in physical anthropology. Stone and Bronze Ages, Vol I & II*. (Ejnar Munksgaard Publishers, 1956).](http://paperpile.com/b/JT7E3Y/Gv6mz)

7. [Bennike, P., Ebbesen, K.& Jørgensen, L. B. The bog find from Sigersdal. Human sacrifice in the Early Neolithic. *J. Dan. Archaeol.* **5**, 85–115 (1986).](http://paperpile.com/b/JT7E3Y/8RQay)

8. [Koch, E. *Neolithic Bog Pots: From Zealand, Møn, Lolland and Falster*. (Det Kongelige Nordiske Oldskriftselskab, 1998).](http://paperpile.com/b/JT7E3Y/NFE8x)

9. [Bennike, P. The Early Neolithic Danish bog finds: a strange group of people!. In Coles, B., Coles, J. & Jørgensen, M.S. (eds.) *Bog bodies, sacred sites and wetland archaeology*, 27–32 (WARP, 1999).](http://paperpile.com/b/JT7E3Y/CmCd9)

10. [Sjögren, K.-G. *et al.* Early Neolithic human bog finds from Falbygden, western Sweden: *Journal of Neolithic Archaeology* 97–126 (2017).](http://paperpile.com/b/JT7E3Y/FtrxP)

11. [Fischer, A. *et al.* Vittrup Man - A genetic foreigner in Neolithic Denmark.](http://paperpile.com/b/JT7E3Y/yPiMd) A combined biomolecular, archaeological and anthropological study (submitted to PlosOne).

12. [Petersen, E. B. Afterlife in the Danish Mesolithic: The Creation, Use and Discarding of ‘Loose Human Bones’. in *Mesolithic burials - rites, symbols and social organisation of early postglacial communities* (eds. Grünberg, J., Gramsch, B., Larsson, L., Orschiedt, J. & Meller, H.) 47–62 (Landesmuseum für Vorgeschichte, 2016).](http://paperpile.com/b/JT7E3Y/oOpaM)

13. [Andersen, N. H. *Sarup Vol. 1. The Sarup Enclosures. The Funnel Beaker Culture of the Sarup Site Including two Causewayed Camps Compared to the Contemporary Settlements in the Area and other European Enclosures*. (Aarhus University Press, 1997).](http://paperpile.com/b/JT7E3Y/2dRyh)

14. [Fischer, A. & Petersen, P. V. A sea of archaeological plenty. in Fischer, A. & Pedersen, L. (eds.) *Oceans of Archaeology* 68–83 (Højbjerg, 2018).](http://paperpile.com/b/JT7E3Y/VQWlY)

#

# 2) Polygenic prediction for height, eye colour and hair colour

Anders Rosengren^1,2^, Vivek Appadurai^1,2^, Andrew Schork^1-4^, Andrés Ingason^1-3^

^1^Institute of Biological Psychiatry, Mental Health Services, Copenhagen University Hospital

^2^iPSYCH, The Lundbeck Foundation Initiative for Integrative Psychiatric Research, Aarhus

^3^Lundbeck Foundation GeoGenetics Centre, GLOBE Institute, University of Copenhagen ^4^Neurogenomics Division, The Translational Genomics Research Institute (TGEN), Phoenix

We predicted relative “genetic” height (i.e. expected increase or decrease in height compared to the mean of the contemporary Danish population, based on common genetic variants) as well as eye and hair colour in the 100 ancient Danish individuals.

The relative genetic height was calculated from summary statistics of a recent GWAS on adult height in the UK Biobank[^1^](https://paperpile.com/c/UTfd9X/qdaOv), using only strand-insensitive autosomal SNPs with robustly genome-wide significant allelic effects (P<1e-15) with imputation info >0.8 and minor allele frequency >0.05 in our ancient imputation genotype dataset. We excluded long-range linkage disequilibrium (LD) regions[^2^](https://paperpile.com/c/UTfd9X/u4qmX) and used the “--clump” function in PLINK[^3^](https://paperpile.com/c/UTfd9X/K7D5q) to prune for LD (r2<0.1 within 10Mb window), rendering a total of 310 effect alleles. Per-sample genetic height score was then calculated for the 100 ancient individuals as well as a subset of 3,467 Danish ancestry male conscripts from a random population subset of the IPSYCH2012 case-cohort[^4^](https://paperpile.com/c/UTfd9X/uLk9O) by summing allelic effect multiplied with the effect allele imputed dosage[^5^](https://paperpile.com/c/UTfd9X/B6ris) across the 310 loci. The genetic height score was moderately correlated with height in the subset of 3,467 Danish ancestry conscripts (r^2^ = 0.095, P = 3.2e-77), and we rescaled the score to a unit corresponding to 1 cm change in predicted height with the median score in the conscript subset as zero. Thus, the PGS in each ancient individual corresponds to the predicted difference in cm from the average of the present-day Danish population, assuming that the scores are equally predictive in males and females. The genetic height score is however limited in two important ways; firstly, the predictive value of the genetic height score is modest and diminishes with general genetic distance to the population in which the allelic effects were determined (in this case European ancestry British); secondly, the score does not take account of important environmental factors such as health and access to nutrition in childhood.

The genetic predictions of eye and hair colour were done based on the HIrisPlex system[^6^](https://paperpile.com/c/UTfd9X/bd99O). Out of 24 main effect HIrisPlex variants, genotype likelihoods of 18 SNPs were available in the ancient sample 1000G imputation, and imputed effect allele dosages of these were used to derive probabilities for brown, blue and grey/intermediate eye colour and, following the HIrisPlex formulas[^6^](https://paperpile.com/c/UTfd9X/bd99O).

The results of genetic prediction of height, eye and hair colour are shown alongside results of other analyses in the composite Fig. 4. When excluding 27 samples with average genomic sequence coverage <0.1x (indicated in shaded colour in Figure 4 and with “0” in the Cov QC column of the Table S2.1 below, the predicted genetic height differed significantly across the three groups defined by the two major population turnover events from Mesolithic Hunter-Gatherer (ML-HG) to Neolithic Early Farmer (NL-EF), and later to people with Neolithic Steppe Pastoralist ancestry (NL-SP), and indicated with thick lines through the panels of Figure 4 (ANOVA chi-square test across all three groups, P=5.1x10^-8^). A follow-up pairwise testing found significant differences between all three group pairs (linear regression, P<0.05 in all instances), with the lowest mean (plus/minus standard error) predicted relative genetic height observed in ML-HG (-1.9 ± 0.3 cm), then NL-EF (-0.3 ± 0.5 cm), and highest in NL-SP (1.2 ± 0.4 cm). It should be noted that the population structure of the ancient Danish individuals, especially the ML-HG and NL-EF groups, is different from the current day European ancestry British population (in which allelic effects for genetic height were estimated), and although we have used only very robustly associated effect alleles to calculate the genetic height score, it is likely that it will not correlate as well with actual height as it does in the current-day European ancestry Danish population (in which the score was rescaled). Therefore, the conclusion that can be drawn from these results is that the common SNP alleles that contribute most strongly to increased height in current day European ancestry populations, were on average of slightly lower frequency in ML-HG, in similar frequency in NL-EF, and slightly higher frequency in NL-SP compared to present day.

Among the 18 HIrisPlex SNPs used to predict eye and hair colour, rs12913832 has the strongest overall dark/light pigmentation effect. At the same time rs12913832 has the lowest average maximum genotype probability (GPmax) of the HIrisPlex SNPs across the ancient Danish imputed genotype dataset. To account for this, we applied a second quality filter when comparing predicted eye and hair colour probabilities across groups, by requiring a GPmax>0.6 for rs12913832 and for at least 15 of the other 17 pigmentation SNPs, which removed a further 17 samples in the cross-group comparison (marked with “0” in the Pigm QC column in Table S2.1 below). In this comparison we did not find a significant difference in probability of brown eye colour (pEye Brown in Table S2.1 below) across the three groups (ANOVA, P=0.21). In contrast, the predicted probability of blond hair colour differed significantly across groups (ANOVA, P=1.1x10^-9^), with the mean likelihood (pHair Blond in Table S2f.1 below) increasing over time from ML-HG (0.05 ± 0.01) to NL-EF (0.25 ± 0.06) and NL-SP (0.43 ± 0.07), although the difference between NL-EF and NL-SP was not significant (P=0.07). Although pigmentation traits are polygenic, many of the HIrisPlex system alleles are so-called main effect alleles and therefore it is likely that the increased predicted probabilities for blond hair over time (and corresponding decrease in predicted probabilities for black hair) represent a true change in the prevalence of dark and light hair colour.

**Table S2.1.** Estimated age, genomic coverage, predicted genetic height difference from current Danish population average, and predicted likelihood of eye and hair colour of 100 ancient Danish individuals sequenced in this study

​​

| **Sample** | **Age (ybp)** | **Age group** | **Genomic coverage** | **Cov QC** | **Pigm QC** | **pHeight (cm)** | **pEye Blue** | **pEye Inter** | **pEye Brown** | **pHair Blond** | **pHair Red** | **pHair Brown** | **pHair Black** |
| --- | --- | --- | --- | --- | --- | --- | --- | --- | --- | --- | --- | --- | --- |
| NEO254 | 10463 | ML-HG | 0.42 | 1 | 0 | -1.2 | 0.09 | 0.13 | 0.78 | 0.04 | 0 | 0.19 | 0.77 |
| NEO13 | 9507 | ML-HG | 0.01 | 0 | 0 | -0.7 | 0.43 | 0.19 | 0.38 | 0.18 | 0 | 0.36 | 0.46 |
| NEO91 | 9122 | ML-HG | 1.18 | 1 | 1 | -1.8 | 0.44 | 0.22 | 0.34 | 0.03 | 0 | 0.26 | 0.71 |
| NEO759 | 9028 | ML-HG | 2.95 | 1 | 1 | 0.2 | 0.58 | 0.16 | 0.26 | 0.04 | 0 | 0.21 | 0.75 |
| NEO587 | 8798 | ML-HG | 1.14 | 1 | 1 | -3.5 | 0.73 | 0.13 | 0.14 | 0.01 | 0 | 0.18 | 0.81 |
| NEO123 | 8182 | ML-HG | 0.29 | 1 | 1 | -3 | 0.09 | 0.13 | 0.78 | 0.11 | 0 | 0.26 | 0.63 |
| NEO19 | 8163 | ML-HG | 3.26 | 1 | 1 | -3.5 | 0.53 | 0.27 | 0.2 | 0 | 0 | 0.28 | 0.72 |
| NEO122 | 8146 | ML-HG | 0.56 | 1 | 0 | -0.4 | 0.12 | 0.22 | 0.66 | 0 | 0 | 0.15 | 0.85 |
| NEO600 | 7817 | ML-HG | 0.10 | 0 | 1 | -0.7 | 0.01 | 0.09 | 0.9 | 0.02 | 0 | 0.28 | 0.7 |
| NEO683 | 7529 | ML-HG | 1.82 | 1 | 1 | -1.8 | 0.26 | 0.25 | 0.49 | 0.01 | 0 | 0.22 | 0.77 |
| NEO932 | 7499 | ML-HG | 2.76 | 1 | 1 | -4.6 | 0.64 | 0.19 | 0.17 | 0.01 | 0 | 0.15 | 0.84 |
| NEO589 | 7478 | ML-HG | 7.41 | 1 | 1 | -1 | 0.46 | 0.21 | 0.33 | 0.02 | 0 | 0.24 | 0.74 |
| NEO748 | 7129 | ML-HG | 0.08 | 0 | 0 | -0.2 | 0.32 | 0.17 | 0.51 | 0.05 | 0 | 0.27 | 0.68 |
| NEO814 | 7125 | ML-HG | 0.06 | 0 | 0 | -2.6 | 0.08 | 0.16 | 0.76 | 0.03 | 0 | 0.24 | 0.73 |
| NEO749 | 7070 | ML-HG | 1.91 | 1 | 1 | -1.3 | 0.57 | 0.19 | 0.24 | 0.03 | 0 | 0.26 | 0.71 |
| NEO791 | 7048 | ML-HG | 2.49 | 1 | 1 | -0.1 | 0.84 | 0.11 | 0.05 | 0.05 | 0 | 0.31 | 0.64 |
| NEO586 | 7031 | ML-HG | 0.20 | 1 | 1 | -1.4 | 0.51 | 0.21 | 0.28 | 0.02 | 0 | 0.28 | 0.7 |
| NEO746 | 6991 | ML-HG | 0.14 | 1 | 0 | -3.1 | 0.16 | 0.15 | 0.69 | 0.11 | 0 | 0.29 | 0.6 |
| NEO583 | 6981 | ML-HG | 0.18 | 1 | 1 | -2.5 | 0.44 | 0.15 | 0.41 | 0.2 | 0 | 0.28 | 0.52 |
| NEO822 | 6978 | ML-HG | 0.06 | 0 | 0 | 0.7 | 0.47 | 0.18 | 0.35 | 0.13 | 0 | 0.32 | 0.55 |
| NEO930 | 6888 | ML-HG | 0.05 | 0 | 0 | -2.1 | 0.4 | 0.18 | 0.42 | 0.07 | 0 | 0.26 | 0.67 |
| NEO733 | 6824 | ML-HG | 1.32 | 1 | 1 | -1.1 | 0.55 | 0.24 | 0.21 | 0.03 | 0 | 0.34 | 0.63 |
| NEO732 | 6815 | ML-HG | 0.13 | 1 | 1 | -4.5 | 0.34 | 0.24 | 0.42 | 0.03 | 0 | 0.36 | 0.61 |
| NEO745 | 6790 | ML-HG | 0.45 | 1 | 1 | -3 | 0.03 | 0.11 | 0.86 | 0.01 | 0 | 0.16 | 0.83 |
| NEO856 | 6777 | ML-HG | 0.56 | 1 | 1 | -2.3 | 0.39 | 0.22 | 0.39 | 0.02 | 0 | 0.19 | 0.79 |
| NEO747 | 6729 | ML-HG | 0.25 | 1 | 0 | -2.1 | 0.12 | 0.25 | 0.63 | 0 | 0 | 0.25 | 0.75 |
| NEO568 | 6586 | ML-HG | 1.98 | 1 | 1 | -4.4 | 0.57 | 0.19 | 0.24 | 0.01 | 0 | 0.26 | 0.73 |
| NEO1 | 6585 | ML-HG | 0.02 | 0 | 0 | 0.8 | 0.09 | 0.15 | 0.76 | 0.1 | 0 | 0.27 | 0.63 |
| NEO941 | 6372 | ML-HG | 0.14 | 1 | 1 | -5 | 0.19 | 0.21 | 0.6 | 0.03 | 0 | 0.29 | 0.68 |
| NEO570 | 6369 | ML-HG | 2.86 | 1 | 1 | -0.6 | 0.72 | 0.15 | 0.13 | 0.01 | 0 | 0.19 | 0.8 |
| NEO751 | 6343 | ML-HG | 0.30 | 1 | 1 | -0.1 | 0.62 | 0.17 | 0.21 | 0.11 | 0 | 0.33 | 0.56 |
| NEO852 | 6308 | ML-HG | 0.19 | 1 | 1 | 1 | 0.44 | 0.24 | 0.32 | 0.02 | 0 | 0.32 | 0.66 |
| NEO855 | 6302 | ML-HG | 1.38 | 1 | 1 | -0.4 | 0.76 | 0.13 | 0.11 | 0.02 | 0 | 0.27 | 0.71 |
| NEO569 | 6142 | ML-HG | 0.67 | 1 | 1 | -2.2 | 0.64 | 0.17 | 0.19 | 0.01 | 0 | 0.2 | 0.79 |
| NEO598 | 6075 | ML-HG | 0.73 | 1 | 1 | -0.5 | 0.09 | 0.15 | 0.76 | 0 | 0 | 0.17 | 0.83 |
| NEO853 | 6047 | ML-HG | 1.96 | 1 | 1 | -1.8 | 0.88 | 0.08 | 0.04 | 0.04 | 0 | 0.3 | 0.66 |
| NEO3 | 5965 | ML-HG | 0.03 | 0 | 0 | -1 | 0.15 | 0.16 | 0.69 | 0.14 | 0 | 0.28 | 0.58 |
| NEO960 | 5926 | ML-HG | 0.15 | 1 | 1 | -3.9 | 0.75 | 0.11 | 0.14 | 0.32 | 0 | 0.32 | 0.36 |
| NEO645 | 5870 | ML-HG | 0.21 | 1 | 1 | -0.4 | 0.85 | 0.09 | 0.06 | 0.09 | 0 | 0.33 | 0.58 |
| NEO962 | 5786 | ML-HG | 0.04 | 0 | 0 | -1.9 | 0.07 | 0.11 | 0.82 | 0.2 | 0 | 0.27 | 0.53 |
| NEO601 | 5753 | NL-EF | 0.08 | 0 | 0 | 1.5 | 0 | 0.02 | 0.98 | 0.01 | 0 | 0.21 | 0.78 |
| NEO790 | 5662 | NL-EF | 0.69 | 1 | 0 | -4.2 | 0.28 | 0.16 | 0.56 | 0.25 | 0 | 0.29 | 0.46 |
| NEO891 | 5661 | NL-EF | 0.60 | 1 | 1 | 1.1 | 0.69 | 0.14 | 0.17 | 0.11 | 0 | 0.35 | 0.54 |
| NEO571 | 5534 | NL-EF | 0.06 | 0 | 0 | -1.5 | 0.11 | 0.15 | 0.74 | 0.29 | 0 | 0.28 | 0.43 |
| NEO23 | 5533 | NL-EF | 3.34 | 1 | 1 | -3.4 | 0.19 | 0.27 | 0.54 | 0.17 | 0 | 0.35 | 0.48 |
| NEO753 | 5531 | NL-EF | 0.16 | 1 | 1 | -0.4 | 0 | 0.03 | 0.97 | 0.02 | 0 | 0.15 | 0.83 |
| NEO942 | 5491 | NL-EF | 0.89 | 1 | 1 | 0.4 | 0.7 | 0.12 | 0.18 | 0.08 | 0 | 0.33 | 0.59 |
| NEO29 | 5489 | NL-EF | 0.53 | 1 | 1 | 3.9 | 0.7 | 0.15 | 0.15 | 0.45 | 0 | 0.31 | 0.24 |
| NEO564 | 5468 | NL-EF | 0.08 | 0 | 0 | -2.6 | 0.35 | 0.18 | 0.47 | 0.13 | 0 | 0.32 | 0.55 |
| NEO41 | 5462 | NL-EF | 0.02 | 0 | 0 | 1.4 | 0.05 | 0.13 | 0.82 | 0.13 | 0 | 0.32 | 0.55 |
| NEO28 | 5459 | NL-EF | 0.92 | 1 | 0 | 2.7 | 0.43 | 0.2 | 0.37 | 0.22 | 0 | 0.3 | 0.48 |
| NEO886 | 5457 | NL-EF | 0.27 | 1 | 1 | -1.9 | 0.7 | 0.13 | 0.17 | 0.66 | 0 | 0.19 | 0.15 |
| NEO866 | 5456 | NL-EF | 1.52 | 1 | 1 | -0.5 | 0.84 | 0.09 | 0.07 | 0.72 | 0 | 0.19 | 0.09 |
| NEO595 | 5452 | NL-EF | 0.22 | 1 | 0 | -2.3 | 0.53 | 0.15 | 0.32 | 0.41 | 0 | 0.28 | 0.31 |
| NEO757 | 5452 | NL-EF | 0.13 | 1 | 0 | -0.3 | 0.05 | 0.11 | 0.84 | 0.03 | 0 | 0.27 | 0.7 |
| NEO896 | 5446 | NL-EF | 0.12 | 1 | 0 | -1.8 | 0.01 | 0.05 | 0.94 | 0.03 | 0 | 0.17 | 0.8 |
| NEO945 | 5445 | NL-EF | 1.38 | 1 | 1 | 0.3 | 0.01 | 0.05 | 0.94 | 0.03 | 0 | 0.22 | 0.75 |
| NEO888 | 5383 | NL-EF | 0.06 | 0 | 0 | 1.5 | 0.09 | 0.13 | 0.78 | 0.18 | 0 | 0.28 | 0.54 |
| NEO933 | 5337 | NL-EF | 0.52 | 1 | 1 | 2.4 | 0.65 | 0.15 | 0.2 | 0.36 | 0 | 0.31 | 0.33 |
| NEO744 | 5333 | NL-EF | 0.22 | 1 | 0 | 3.6 | 0.03 | 0.12 | 0.85 | 0.22 | 0 | 0.32 | 0.46 |
| NEO795 | 5333 | NL-EF | 0.03 | 0 | 0 | -1 | 0.13 | 0.16 | 0.71 | 0.2 | 0 | 0.33 | 0.47 |
| NEO702 | 5263 | NL-EF | 0.15 | 1 | 1 | -0.7 | 0.52 | 0.24 | 0.24 | 0.02 | 0 | 0.31 | 0.67 |
| NEO7 | 5242 | NL-EF | 0.01 | 0 | 0 | -1.1 | 0 | 0.04 | 0.96 | 0.03 | 0 | 0.23 | 0.74 |
| NEO597 | 5210 | NL-EF | 0.18 | 1 | 0 | 0.5 | 0.1 | 0.14 | 0.76 | 0.31 | 0 | 0.29 | 0.4 |
| NEO935 | 5187 | NL-EF | 5.03 | 1 | 1 | -1.4 | 0.09 | 0.15 | 0.76 | 0.44 | 0 | 0.27 | 0.29 |
| NEO865 | 5179 | NL-EF | 0.09 | 0 | 0 | -3.2 | 0.01 | 0.06 | 0.93 | 0.06 | 0 | 0.23 | 0.71 |
| NEO594 | 5174 | NL-EF | 0.05 | 0 | 0 | -1.8 | 0.47 | 0.19 | 0.34 | 0.31 | 0 | 0.33 | 0.36 |
| NEO961 | 5137 | NL-EF | 0.02 | 0 | 0 | 1.1 | 0.24 | 0.19 | 0.57 | 0.22 | 0 | 0.33 | 0.45 |
| NEO599 | 5134 | NL-EF | 0.19 | 1 | 0 | -2.6 | 0.02 | 0.11 | 0.86 | 0.08 | 0 | 0.24 | 0.68 |
| NEO602 | 5134 | NL-EF | 0.09 | 0 | 0 | -1.4 | 0.25 | 0.17 | 0.58 | 0.47 | 0 | 0.25 | 0.28 |
| NEO566 | 5130 | NL-EF | 0.02 | 0 | 0 | -3.2 | 0.44 | 0.17 | 0.39 | 0.38 | 0 | 0.28 | 0.34 |
| NEO33 | 5128 | NL-EF | 0.05 | 0 | 0 | -3.1 | 0.07 | 0.15 | 0.78 | 0.02 | 0 | 0.2 | 0.78 |
| NEO898 | 5080 | NL-EF | 3.8 | 1 | 1 | -4 | 0.51 | 0.13 | 0.36 | 0.21 | 0 | 0.3 | 0.49 |
| NEO43 | 5067 | NL-EF | 0.11 | 1 | 0 | 1.9 | 0.05 | 0.14 | 0.81 | 0.17 | 0 | 0.36 | 0.47 |
| NEO25 | 4956 | NL-EF | 0.36 | 1 | 1 | 1.1 | 0.09 | 0.16 | 0.75 | 0.08 | 0 | 0.33 | 0.59 |
| NEO925 | 4947 | NL-EF | 0.29 | 1 | 0 | -2.8 | 0 | 0.03 | 0.97 | 0.05 | 0 | 0.25 | 0.7 |
| NEO943 | 4614 | NL-EF | 1.75 | 1 | 1 | 2.1 | 0.03 | 0.07 | 0.9 | 0.2 | 0 | 0.26 | 0.54 |
| NEO580 | 4611 | NL-EF | 0.01 | 0 | 0 | -1.3 | 0.03 | 0.09 | 0.88 | 0.09 | 0 | 0.28 | 0.63 |
| NEO792 | 4493 | NL-SP | 0.25 | 1 | 1 | -0.4 | 0.04 | 0.09 | 0.87 | 0.14 | 0 | 0.27 | 0.59 |
| NEO876 | 4338 | NL-SP | 0.05 | 0 | 0 | -2.4 | 0.01 | 0.08 | 0.91 | 0.16 | 0 | 0.31 | 0.53 |
| NEO870 | 4240 | NL-SP | 0.58 | 1 | 1 | 2.2 | 0.23 | 0.26 | 0.51 | 0.37 | 0 | 0.33 | 0.3 |
| NEO92 | 4188 | NL-SP | 0.61 | 1 | 1 | -0.9 | 0.82 | 0.11 | 0.07 | 0.48 | 0 | 0.25 | 0.27 |
| NEO737 | 4106 | NL-SP | 0.24 | 1 | 1 | 1 | 0.77 | 0.15 | 0.08 | 0.27 | 0 | 0.41 | 0.32 |
| NEO738 | 4103 | NL-SP | 1.21 | 1 | 1 | -0.3 | 0.92 | 0.05 | 0.03 | 0.73 | 0 | 0.17 | 0.1 |
| NEO861 | 4102 | NL-SP | 0.38 | 1 | 1 | 0.5 | 0.79 | 0.11 | 0.1 | 0.74 | 0 | 0.17 | 0.09 |
| NEO878 | 4026 | NL-SP | 0.28 | 1 | 1 | -2.3 | 0.45 | 0.15 | 0.4 | 0.37 | 0 | 0.33 | 0.3 |
| NEO872 | 3979 | NL-SP | 0.08 | 0 | 0 | -0.1 | 0.62 | 0.15 | 0.23 | 0.44 | 0 | 0.3 | 0.26 |
| NEO735 | 3972 | NL-SP | 0.67 | 1 | 1 | 2.5 | 0.78 | 0.11 | 0.11 | 0.76 | 0.01 | 0.16 | 0.07 |
| NEO875 | 3970 | NL-SP | 0.18 | 1 | 0 | 1.6 | 0.07 | 0.11 | 0.82 | 0.18 | 0 | 0.27 | 0.55 |
| NEO739 | 3965 | NL-SP | 1.88 | 1 | 1 | 0.9 | 0.01 | 0.04 | 0.95 | 0.02 | 0 | 0.15 | 0.83 |
| NEO934 | 3809 | NL-SP | 0.08 | 0 | 0 | 2 | 0.19 | 0.15 | 0.66 | 0.29 | 0 | 0.31 | 0.4 |
| NEO93 | 3735 | NL-SP | 1.98 | 1 | 1 | -0.3 | 0.26 | 0.25 | 0.49 | 0.1 | 0 | 0.39 | 0.51 |
| NEO860 | 3697 | NL-SP | 0.20 | 1 | 0 | 1.2 | 0.55 | 0.2 | 0.25 | 0.66 | 0 | 0.21 | 0.13 |
| NEO857 | 3637 | NL-SP | 0.04 | 0 | 0 | -1.9 | 0.09 | 0.17 | 0.74 | 0.26 | 0 | 0.31 | 0.43 |
| NEO752 | 3589 | NL-SP | 2.09 | 1 | 1 | 1.8 | 0.64 | 0.12 | 0.24 | 0.31 | 0 | 0.31 | 0.38 |
| NEO815 | 3471 | NL-SP | 0.11 | 1 | 0 | 4.3 | 0.48 | 0.24 | 0.28 | 0.33 | 0.04 | 0.42 | 0.21 |
| NEO563 | 3350 | NL-SP | 0.81 | 1 | 1 | 3.6 | 0.69 | 0.14 | 0.17 | 0.59 | 0.09 | 0.19 | 0.13 |
| NEO590 | 3290 | NL-SP | 1.01 | 1 | 1 | 2.9 | 0.85 | 0.07 | 0.08 | 0.84 | 0.01 | 0.11 | 0.04 |
| NEO951 | 3242 | NL-SP | 0.45 | 1 | 0 | 1.7 | 0.01 | 0.06 | 0.93 | 0.2 | 0 | 0.34 | 0.46 |
| NEO946 | 3094 | NL-SP | 1.24 | 1 | 1 | 2.2 | 0.8 | 0.1 | 0.1 | 0.28 | 0.01 | 0.37 | 0.34 |

### References

1. [Bycroft, C. *et al.* The UK Biobank resource with deep phenotyping and genomic data. *Nature* **562**, 203–209 (2018).](http://paperpile.com/b/UTfd9X/qdaOv)

2. [Price, A. L. *et al.* Long-range LD can confound genome scans in admixed populations. *American journal of human genetics* vol. 83 132–5; author reply 135–9 (2008).](http://paperpile.com/b/UTfd9X/u4qmX)

3. [Purcell, S. *et al.* PLINK: a tool set for whole-genome association and population-based linkage analyses. *Am. J. Hum. Genet.* **81**, 559–575 (2007).](http://paperpile.com/b/UTfd9X/K7D5q)

4. [Pedersen, C. B. *et al.* The iPSYCH2012 case-cohort sample: new directions for unravelling genetic and environmental architectures of severe mental disorders. *Mol. Psychiatry* **23**, 6–14 (2018).](http://paperpile.com/b/UTfd9X/uLk9O)

5. [Appadurai, V. *et al.* Accuracy of haplotype estimation and whole genome imputation affects complex trait analyses in complex biobanks. *Commun Biol* **6**, 101 (2023).](http://paperpile.com/b/UTfd9X/B6ris)

6. [Walsh, S. *et al.* The HIrisPlex system for simultaneous prediction of hair and eye colour from DNA. *Forensic Sci. Int. Genet.* **7**, 98–115 (2013).](http://paperpile.com/b/UTfd9X/bd99O)

# 3) Bayesian chronological models of the transition

Bettina Schulz Paulsson^1^

^1^Department of Historical Studies, University of Gothenburg, 405 30 Gothenburg, Sweden

*Note: Dates in this supplementary note are consistently expressed as cal. BC, whilst in the rest of the supplements and manuscript, these are cal. BP.*

### Introduction

Previous analyses of the temporality of ancient genomes have produced coarse chronologies, which tend to mask possible short-term events such as those related to rapid population movement, pandemics or socio-cultural changes. Here, we adopted a Bayesian approach to investigate a high resolution chronology of the major demographic events we describe in this study. With this approach it is possible to unify radiocarbon results, detailed archaeogenetic information, and the high precision curve into one calibration process [^e.g. 1–13^](https://paperpile.com/c/jM78vL/zGyDr+Un1TM+uvanT+cQvCu+wnTRW+cTuvh+EfZgy+JPLgQ+YMkJu+SYZmQ+XCFYH+7NG5I+zMU8w/?prefix=e.g.,,,,,,,,,,,,), thereby gaining much greater precision on the timing

We established a highly precise chronology for 81 radiocarbon dates from 64 Danish sites focusing on estimating the overlap and transition times between the different ancestries. The primary aims were to estimate; i) the timing of the arrival of Neolithic (Anatolian-derived) ancestry in Denmark; ii) the period of overlap with Mesolithic hunter-gatherer ancestry, and iii) the total time span of Neolithic (Anatolian-derived) ancestry in Denmark. There were not sufficient Late Neolithic and Bronze Age samples to estimate the period of overlap for this second transition in ancestry.

All the models and calibrated data presented in this analysis have been performed utilising OxCal v4.4[^4,7,9,10^](https://paperpile.com/c/jM78vL/cQvCu+EfZgy+SYZmQ+YMkJu/?noauthor=0,1,1,1) and forms of outlier analysis and the calibration dataset from Reimer et al.[^14^](https://paperpile.com/c/jM78vL/vmPj6/?noauthor=1), Intcal20. OxCal can accomplish automated ‘wiggle matching’ and calculate probability distributions for samples in sequences and phases.

For the Bayesian approach, the models had to be defined first in OxCal in sequences and phases making use of all available information generated from the human remains (such as the genetic ancestries, or vertical and horizontal stratigraphy). Depending on the problems or questions that arose, boundaries were determined and incorporated into the model structure. The program first calculates the probability distribution of each radiocarbon result and then attempts to reconcile this information by a repeated sampling of the distribution of these dates with the information previously determined. Thus, it builds up a set of solutions consistent with the structure of the model[^6,10,14^](https://paperpile.com/c/jM78vL/SYZmQ+cTuvh+vmPj6).

A trapezoidal phase prior[^15,16^](https://paperpile.com/c/jM78vL/FuREb+abevx) is used in a contiguous framework to model the transitional phases of the different ancestry groups. For the reconstruction of the movement of the Neolithic farmers we tested different models defining the phases by taking possible horizontal stratigraphies of the sampled material into consideration. Posterior beliefs are expressed as probability distributions known as ‘posterior density estimates’ and they are always given in *italics.*

### Materials

Of the 100 radiocarbon-dated individuals studied here, 81 were relevant for the questions outlined above, representing individuals with hunter-gatherer and Neolithic (Anatolian-derived) ancestries for use in the Bayesian models. The remaining data stem from Late Neolithic and Bronze Age individuals with Steppe-derived ancestry.

We corrected the reservoir effect on bones with significantly elevated isotope values (δ^13^C -18.00 and δ^15^N +12.00) directly in the models using previously defined reservoir ages and calculated the DRE (diet reconstruction estimates) for the individual in ^14^C years based on the collagen isotope values (Allentoft et al. 2023, SI Note 8, SI Table III-IV); for a similar method see [^17,18^](https://paperpile.com/c/jM78vL/YeoGT+tsQp3).

Reservoir ages affected predominantly samples with hunter-gatherer ancestry. The values for the Neolithic populations indicate a mainly terrestrial diet and in most cases there is no clear evidence for reservoir effects on human bones. For combining radiocarbon dates related to the same individual we used the R_Combine() function. For four of the radiocarbon dates the X-Test failed (Viksø Mose /X-Test failed at 5% df=1 T=3.849(5% 3.8, Tudse Hage, X2-Test: df=1 T=7.510(5% 3.8), Dragsholm B X2-Test: df=3 T=24.652(5% 7.8). In those situations, the earlier dates were excluded from the models, as they likely exhibit contamination.

Furthermore we excluded the earliest time points in our dataset (n=5), all from the Mesolithic Maglemose phase (7-8th millennium cal. BC, 9-19th millennium cal. BC) to calculate the time interval for the transition to Neolithic (Anatolian-derived) farmer ancestry more accurately.

#### OxCal Model Details and Outputs

The datasets in Figs. S3.1 and S3.2 provide the OxCal model details and outputs for the Bayesian statistical framework of the analysis. OxCal´s chronological query language (CQL) was defined in Bronk Ramsey [^9,10^](https://paperpile.com/c/jM78vL/SYZmQ+YMkJu/?noauthor=0,1) and the model specifications are presented in this form. Figures S3.2 illustrates model outcomes, including the un-modelled and modelled calibrated ranges in their 68.2% and 95.4% probability. In order to test for unreliable chronological models or intrusive material, the agreement index A is calculated to exclude inconsistencies from a model with a threshold value of ~100%. This threshold value can be higher or it can decrease down to 60%. The agreement index A overall is calculated as a whole for the model^5^, which is likewise ~100%, it should likewise not fall below 60%. These two indices represent a threshold value analogue to the 0.05 significance level in a χ²-test. Furthermore, a posterior outlier probability is calculated for each of the radiocarbon dates in the models and represented with the model outcomes in the datasets (Fig. S3.2).

#### Estimating the Transition Phase and span of Neolithic farmer ancestry

In this study, we used a trapezoidal phase prior[^15,16^](https://paperpile.com/c/jM78vL/FuREb+abevx) instead of a uniform prior (for abrupt transition) for calculation the transitional time intervals because:

• The different phases can be overlapped, indicating transitional periods.

• Changes in the genetic ancestries across whole regions can be characterised as slow, non-instantaneous processes such as e.g. migrations. These processes probably lasted for a longer period of time

This framework assumes that the different phases and genetic ancestries (hunter-gatherer, European farmers) are contiguous to each other and thus also allows time periods representing phases of transition. The trapezoidal models are used to calculate the highest probability for the co-existence of the groups, e.g. the time interval from the earliest possible appearance of the first farmers with Anatolian-derived ancestry to the last appearance of Mesolithic hunter-gatherer DNA. Such a trapezoid model produces information across three components: first, a gradual increase (introductory period); then, a period of constant rate of activity (blooming period); and finally, a gradual decrease (period of decline)[^16^](https://paperpile.com/c/jM78vL/abevx/?locator=107).

### Results

A Bayesian trapezoidal model was applied on 81 radiocarbon dates with genomic data from altogether 64 individuals. The presented end model shows with A_model_ 104.4 and A_overall_ 90.2 at very high agreement. The resultant model for the end date of this transition shows very high agreement between A_model_=104.4 and A_overall_=90.2 (Fig. S3.2).

The ages of individuals with hunter-gatherer ancestry were corrected with regional reservoir ages of between 350y and 250y (for the reservoir ages see Table S1 and Allentoft et al. 2022). After excluding the earliest Mesolithic individuals in our sequence (see above) the sequence starts with an individual from Røntest, which is calculated to *6217-5920 cal. BC (95.4%; 6079-5990 cal. BC, 68.2%).*

The hunter-gatherer genetic ancestry component disappears from Denmark at the transition from the 5th to the 4th millennium cal. BC. The last known hunter-gatherers in this phase are represented by the individuals from Rødhals and Ravnsbjerggård (Rødhals, *3961-3811 cal. BC, 68.2%; 4035-3769 cal. BC, 95.4*% and Ravnsbjerggård *3965-3809 cal. BC, 68.2%; 4040-3716 cal. BC, 95.4%*).

The earliest known individual with Neolithic (Anatolian-derived) farmer genetic ancestry is from Viksø Mose (5050 +/- 15 BP, UCIA-232706). The time of death of this individual is thus calculated to *3934-3774 cal. BC (95.4%; 3880-3782 cal. BC, 68.2%),* followed by the second oldest individual with this ancestry in our data, namely Tysmose II *3791-3645 cal. BC* *(95.4%; 3752-3653 cal. BC, 68.2 %*).

The gradual increase or the introductory period of the Early European Farmers ancestry in Denmark proceeds within *4085-3796 cal. BC (95.4%; 3965-3824 cal. BC, 68.2%).* The time intervals for the 95.4% probability of the latest Hunter-Gatherer ancestry and the early European farmer ancestry are overlapping, and the transition from the earliest possible European Farmer ancestry to the last possible Hunter-Gatherer ancestry spans *186-416 years (68.2%)* and *93-579 years (95.4%)* (Figs. S3.3-5). These values represent the maximum possible overlaps or co-existence of the two ancestry groups. The final transition from hunter-gatherer ancestry to Neolithic (Anatolian-derived) ancestry occurs between *3735-3458 cal. BC (95.4%; 3668-3538, 68.2%)*. Within this time interval the last Hunter gatherer ancestry in Denmark disappears. Radiocarbon measurements for 38 individuals with Neolithic (Anatolian-derived) ancestry were utilised in these calculations. This ancestry component is present in Denmark until *2886-2619 cal. BC (95.4%; 2876-2737 cal. BC, 68.2%,* Stenderup Hage, UBA-39152). The duration of the presence of Anatolian-derived ancestry is thus calculated to between *936-1010 years, 68.2%* and to between *876-1100 years 95.4%* (figure S8.6). In conclusion Neolithic (Anatolian-derived) ancestry was present for around 1000 years in Denmark before being replaced by the second population turnover introducing Steppe-derived DNA in the Late Neolithic.

We note that in the Middle Neolithic, ~200-300 years after the disappearance of the last Mesolithic people of typical western hunter gatherer ancestry in Denmark a few individuals with Scandinavian peninsula hunter-gatherer ancestry reached the Danish coasts, as evident in our data. Vittrup Man belongs to this ancestry group. He died between *3368 and 3104 cal. BC* (95.4%; *3363-3125 cal. BC,* 68.2%).

Plot()

{

Sequence(Neolithisation Denmark)

{

Boundary("Start 1")

{

Start("Start of Start 1");

Transition("Period of Start 1");

End("End of Start 1");

};

Phase("Ancestry Group Hunter-Gatherer West")

{

Delta_R("DRE Røntestenen,AAR-11355", 350,40);

R_Date("Røntesten,AAR- 11355",7542,42);

Phase(Orehoved Sejlrende)

{

Delta_R("DRE Orehoved Sejlrende,UBA-37880", 273,18);

R_Date("Orehoved Sejlrende,UBA- 37880",7505,36);

Delta_R("DRE Orehoved Sejlrende, UBA-38222 ", 273,18);

R_Date("Orehoved Sejlrende, UBA-38222",7415,53);

};

Delta_R("DRE Vedbæk Boldbaner,Ua-23792", 273,18);

R_Date("Vedbæk Boldbaner,Ua-23792",7115,55);

Delta_R("DRE Tybrind Vig,AAR-9342 ", 350,40);

R_Date("Tybrind Vig,AAR-9342",6905,55);

Delta R("DRE Tudse Hage,UBA-38242,273,18")

R_Date("Tudse Hage,UBA-38242",6608,47);

Delta_R("DRE Korsør Nor,K-4263", 273,18);

R_Date("Korsør Nor,K-4263",6760,75);

Delta_R("DRE Koed IV,UBA-35710", 350,40);

R_Date("Koed IV,UBA-35710",6443,44);

Delta_R("DRE Bodal K,UBA-38238", 273,18);

R_Date("Bodal K,UBA-38238",6435,44);

Phase(Bøgebakken, Vedbæk)

{

Delta_R("DRE Ua-23787", 273,18);

R_Date("Ua-23787",6420,70);

Delta_R("DRE UBA-35717", 273,18);

R_Date("UBA-35717",6298,58);

Delta_R("DRE Ua-23794", 273,18);

R_Date("Ua-23794",6260,75)

Delta_R("DRE Ua-23784", 273,18);

R_Date("Ua-23784",6140,50);

Delta_R("DRE Ua-23786", 273,18);

R_Date("Ua-23786",6060,60);

};

Delta_R("DRE Koed I,UBA-39552",350,40);

R_Date("Koed I,UBA-39552",6400,37);

Delta_R("DRE Fannerup F, AAR-19687", 350,40);

R_Date("Fannerup F, AAR-19687",6377,30);

Phase(Dagsholm)

{

Delta_R("DRE AAR-8725", 273,18);

R_Date("AAR-8725",6310,60);

R_Combine Delta_R ("DRE A Dragsholm",273,18)

{

R_Date("AAR-7414-2",6209,40);

R_Date ("NZA-15676",6208,60);

R_Date("AAR-7414",6187,43);

R_Date("NZA-15953",6115,60);

};

R_Combine Delta_R("DRE B Dragsholm", 273,18)

{

R_Date("NZA-15954",6030,55);

R_Date("NZA-15678",6002,60);

};

};

Delta_R("DRE Nederst,AAR-28401", 350,40);

R_Date("Nederst,AAR-28401",6289,45);

Delta_R("DRE Korsør Nor,AAR-10246", 273,18);

R_Date("Korsør Nor,AAR-10246",6263,36);

R_Combine Delta_R("DRE Holmegard-Djursland", 350,40)

{

R_Date("OxA-533",6080,80);

R_Date("K-3559",6030,80);

};

Delta_R("DRE Havnø,UBA-39153", 350,40);

R_Date("Havnø,UBA-39153",5947,33);

Delta_R("DRE Fannerup E, UBA-35705", 350,40);

R_Date("Fannerup E,UBA-35705",5911,43);

Delta_R("DRE Fannerup F, AAR-28400", 350,40);

R_Date("Fannerup F, AAR-28400",5821,37);

Delta_R("DRE Bjørnsholm,UBA-35718", 375,40);

R_Date("Bjørnsholm,UBA-35718",5792,41);

Delta_R("DRE Norsminde,AAR-8556", 350,40);

R_Date("Norsminde, AAR-8556",5800,35);

Phase(Ertebølle)

{

Delta_R("DRE UBA-35704", 375,40);

R_Date("UBA-35704",5611,53);

Delta_R("DRE UBA-31308", 375,40);

R_Date("UBA-31308",5690,34);

};

Delta_R("DRE Vængesø, K-3921", 350,40);

R_Date("K-3921",5540,40);

Delta_R("DRE Sølager, K-3921", 273,18);

R_Date("Sølager, K-3921",5540,40);

Delta_R("DRE Langø Skaldynge, UBA-37896", 273,18);

R_Date("Langø Skaldynge UBA-37896",5496,57);

Delta_R("DRE Rødhals,AAR-8552", 273,18);

R_Date("Rødhals,AAR-8552",5360,50);

Delta_R("DRE Ravnsbjerggård,AAR-10993", 260,40);

R_Date("Ravnsbjerggård, AAR-10993",5308,43);

R_Date("Dragsholm II/D, AAR-7416-2",5102,37);

};

Boundary("Transition 1/2")

{

Start("Start of Transition 1/2");

Transition("Period of Transition 1/2");

End("End of Transition 1/2");

};

Phase("Ancestry Group Farmer Central Europe")

{

R_Date("Viksø Mose, UCIA-232706",5050,15);

R_Date("Tysmose II,UBA-35722",4959,43);

R_Date("Roskilde Fjord,UBA-37910",4939,45);

R_Date("Pandebjerg, UCIAMS-232705",4910,15);

Phase(Rude)

{

R_Date("UBA-37877",4901,37);

R_Date("UBA-37876",4838,29);

R_Date("UCIAMS",4725,15);

};

R_Date("Sigersdal Mose,UBA-39125",4853,29);

R_Date("Tissøe,UBA-39159",4846,53);

R_Date("Lohals Nord,UBA-35699",4843,40);

R_Date("Bygholm, grav I,UBA-38227",4836,35);

R_Date("Grøfte A,UBA-38228",4828,35);

R_Combine("Salpetermosen")

{

R_Date("AAR-21343",4789,25);

R_Date("AAR-21344",4752,29);

};

R_Date("Dalmosegaard (Borre),UBA-39141",4774,52);

R_Date("Sejerby (Sejerø,UBA-35721",4746,45);

R_Date("Lundby-Falster,UBA-40439",4743,31);

R_Date("Jorløse Mose,AAR-11122",4720,40);

R_Date("Porsmose ,K-3748",4710,90);

R_Date("Vig Femhøve, UBA-37893",4709,52);

R_Date("Mandemarke, UBA-39554",4698,32);

R_Date("Femhøve,UBA-37893",4709,52);

R_Date("Lendemark,UBA-39554",4698,32);

R_Date("Lundby-Falster,UBA-39128",4688,31);

R_Date("Sludegård Sømose,UBA-39145",4688,56);

R_Date("Læsten Mose,UBA-39151",4674,51);

R_Date("Elkenøre,UBA-40440",4647,31);

R_Date("Døjringe I,UBA-40108",4629,31);

R_Date("Jørlundegaard,UBA-35714",4619,41);

R_Date("Vibygårds Mose,UBA-39147",4573,33);

R_Date("Storelyng (Øgårde boat III), K-3746",4570,60);

Phase(Hunter-Gatherer Baltic)

{

R_Combine (Vittrup)

{

R_Date("UBA-39121",4565,29);

R_Date("UBA-29904",4464,52);

};

Delta_R ("DRE Svinninge Vejle, UBA-37912",273,18);

R_Date("Svinninge Vejle, UBA-37912", 4539,72);

};

R_Date("Storelyng Østrup,AAR-10248",4523,37);

R_Date("Neverkær Mose I,UBA-38232",4518,33);

R_Date("Avlebjerg,UBA-40443",4510,32);

R_Date("Vanløse Mose II,AAR-10994",4485,41);

R_Date("Kainsbakke,AAR-21424",4464,29);

R_Date("Klokkehøj,UBA-35708",4086,42);

R_Date("Stenderup Hage,UBA-39152",4072,61);

};

Span("Neolithic ancestry");

Interval("Neolithic");

Boundary("End 2")

{

Start("Start of End 2");

Transition("Period of End 2");

End("End of End 2");

};

};

};

**Figure S3.1. CQL code for the trapezoidal model of the Hunter-Gatherer West/European Farmer transition.**

***
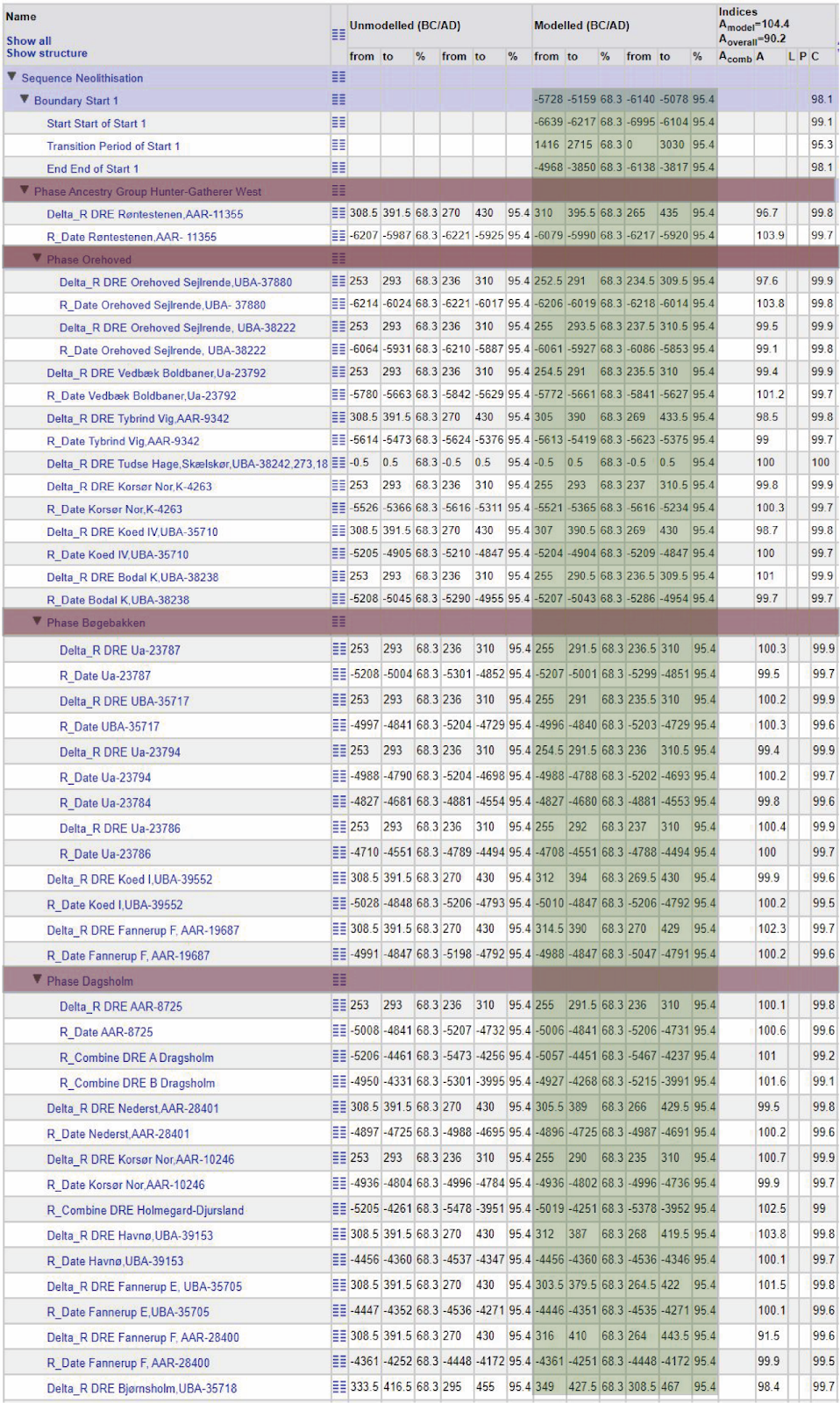
***

***
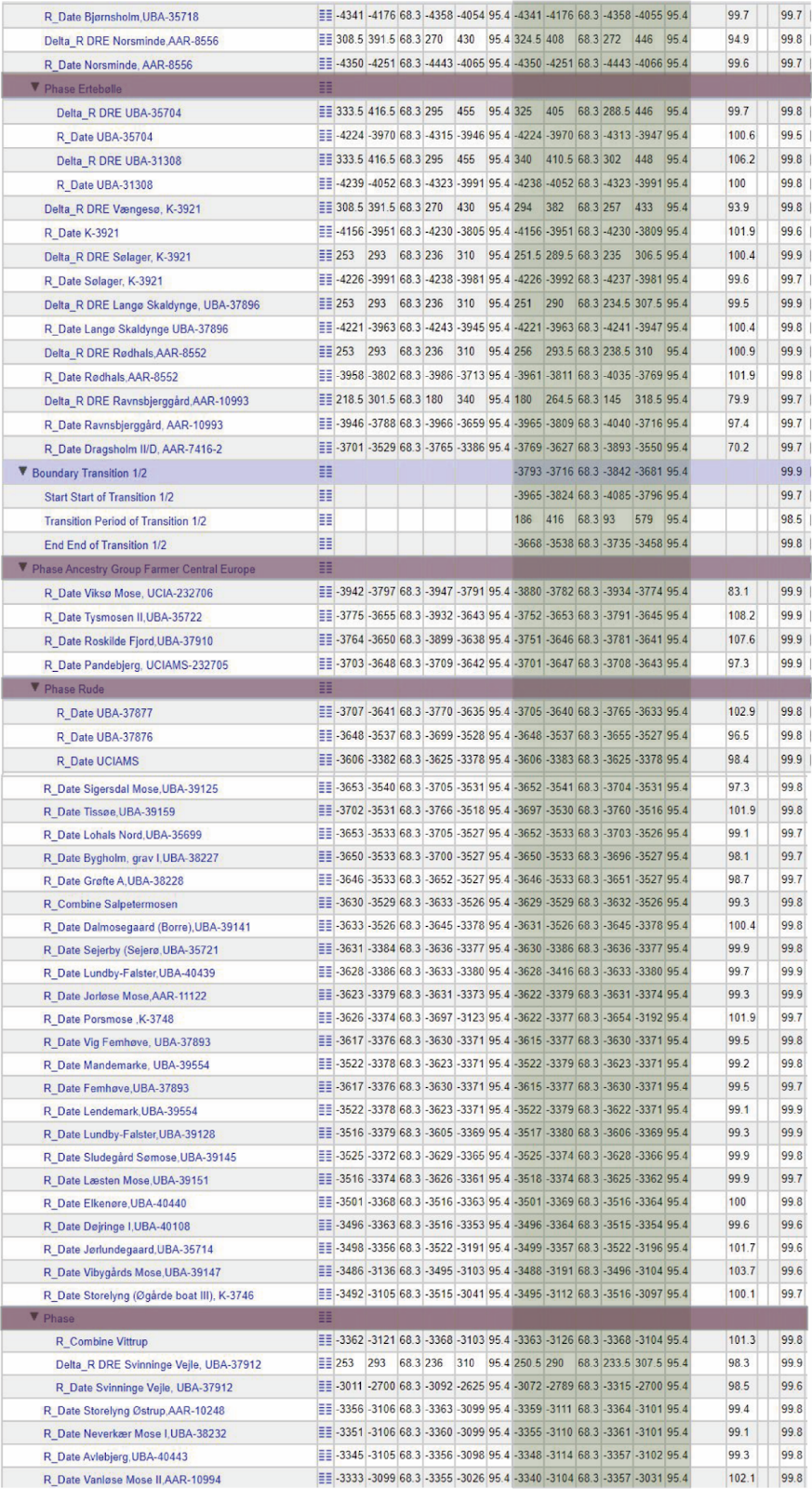
***

***
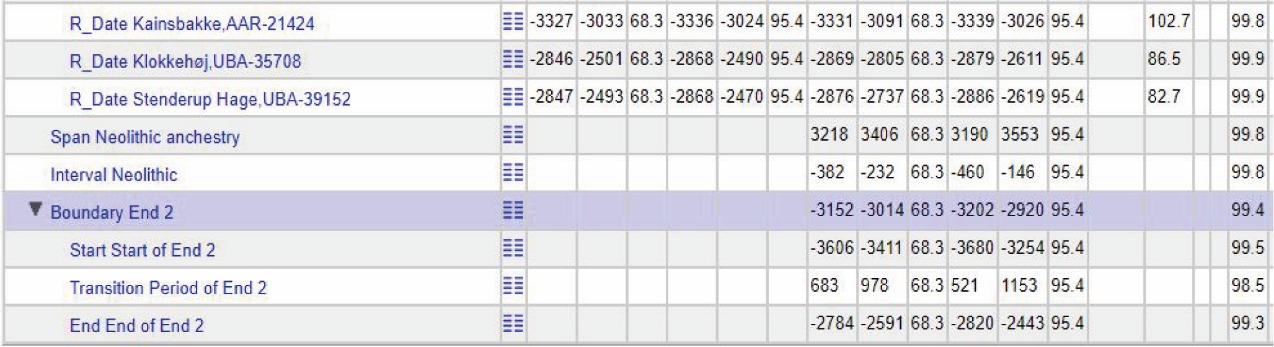
***

**Figure S3.2. Model outcomes for the trapezoidal model of Hunter-Gatherer West/European Farmer transition.** Green indicates modelled data, purple indicates phases.

**Figure S3.3. Interval period for the Hunter-Gatherer West/European Farmer transition.** This figure and figures S3.4-5 illustrate a period of constant rate of activity (blooming period); gradual increase (introductory period) and gradual decrease (period of decline) for the trapezoidal model transition Hunter-Gatherer West/European Farmer.
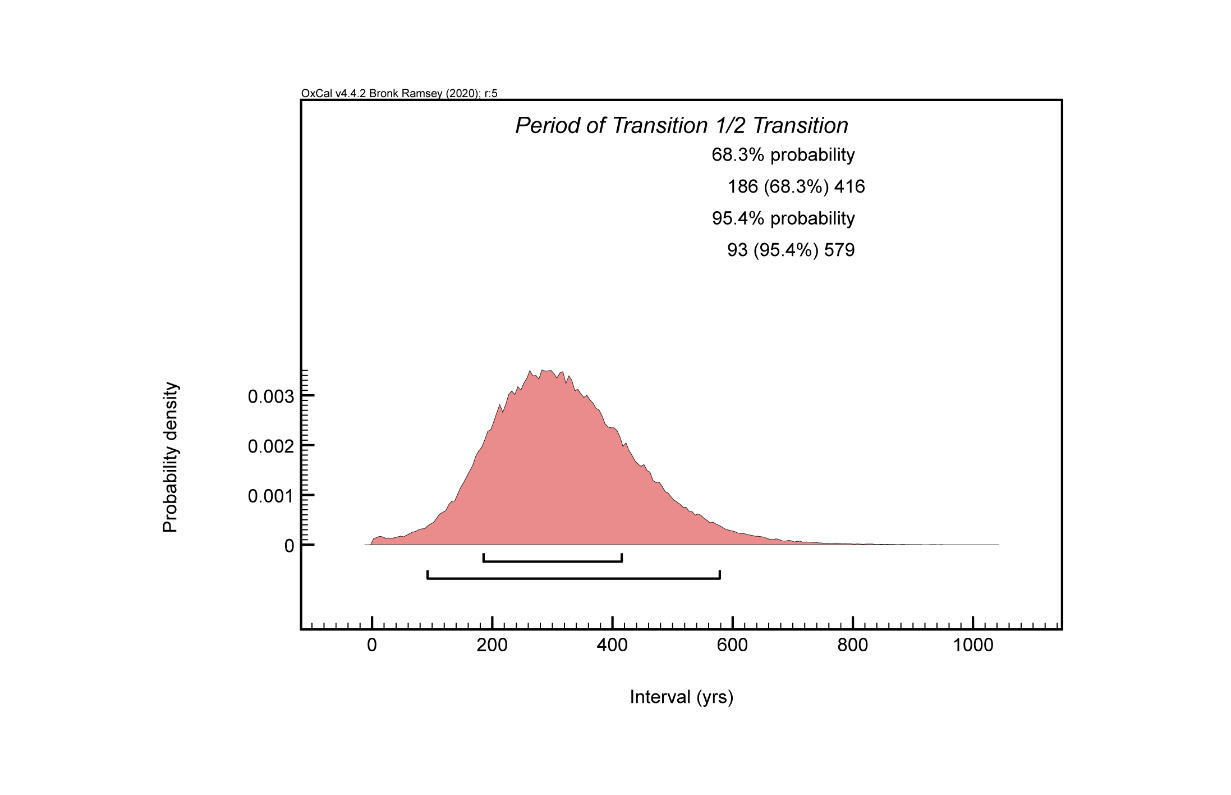


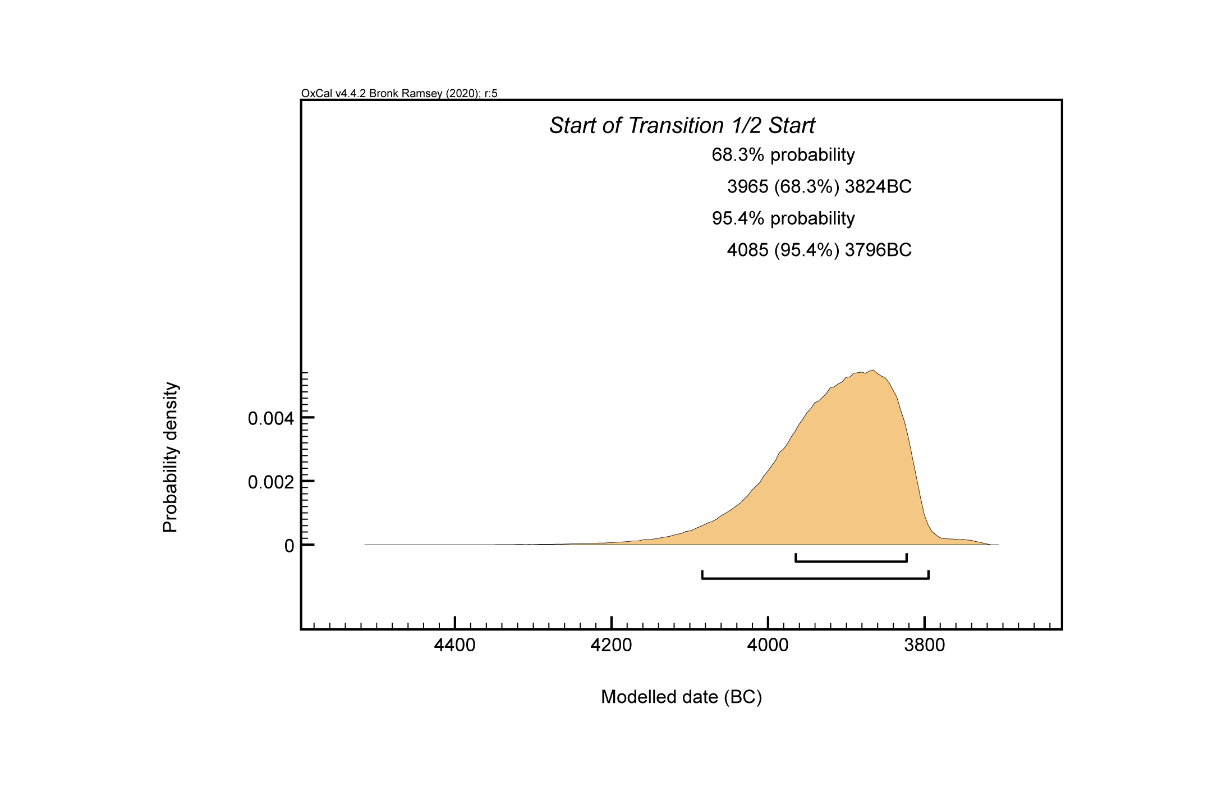


**Figure S3.4. Modelled start date for the Hunter-Gatherer West/European Farmer transition.**


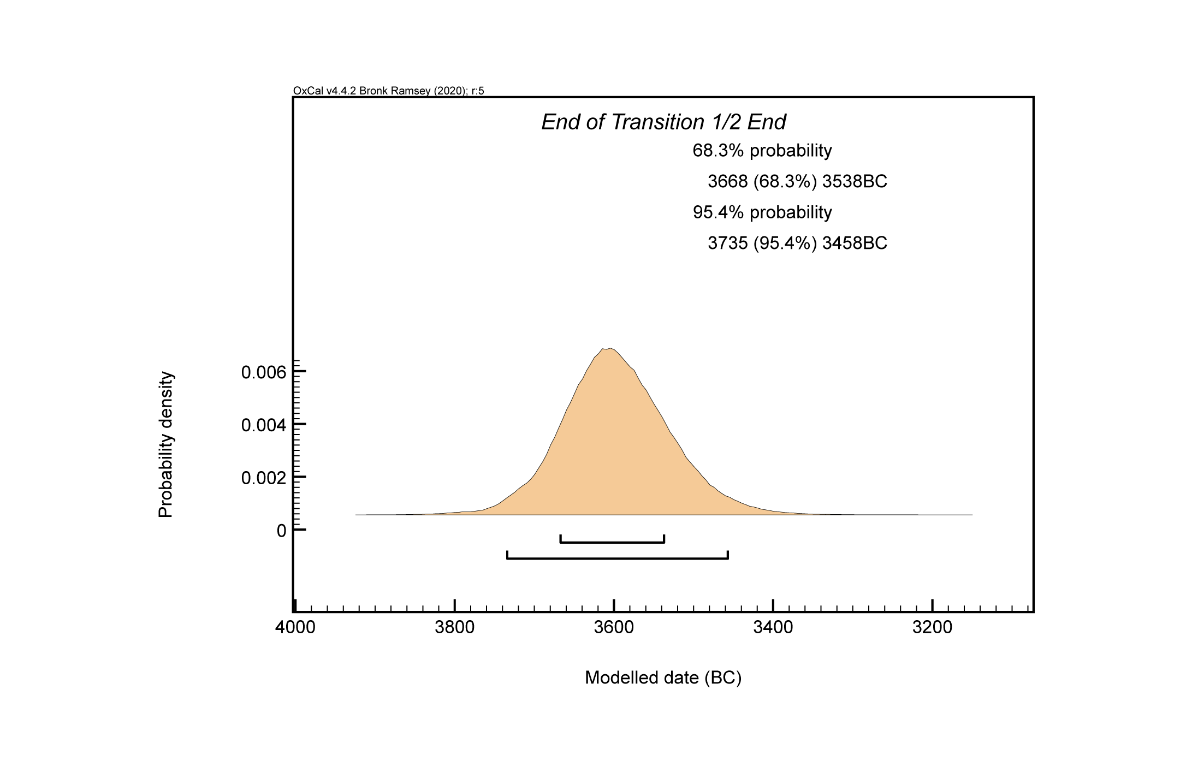


**Figure S3.5. Modelled end date for the Hunter-Gatherer West/European Farmer transition.**

**
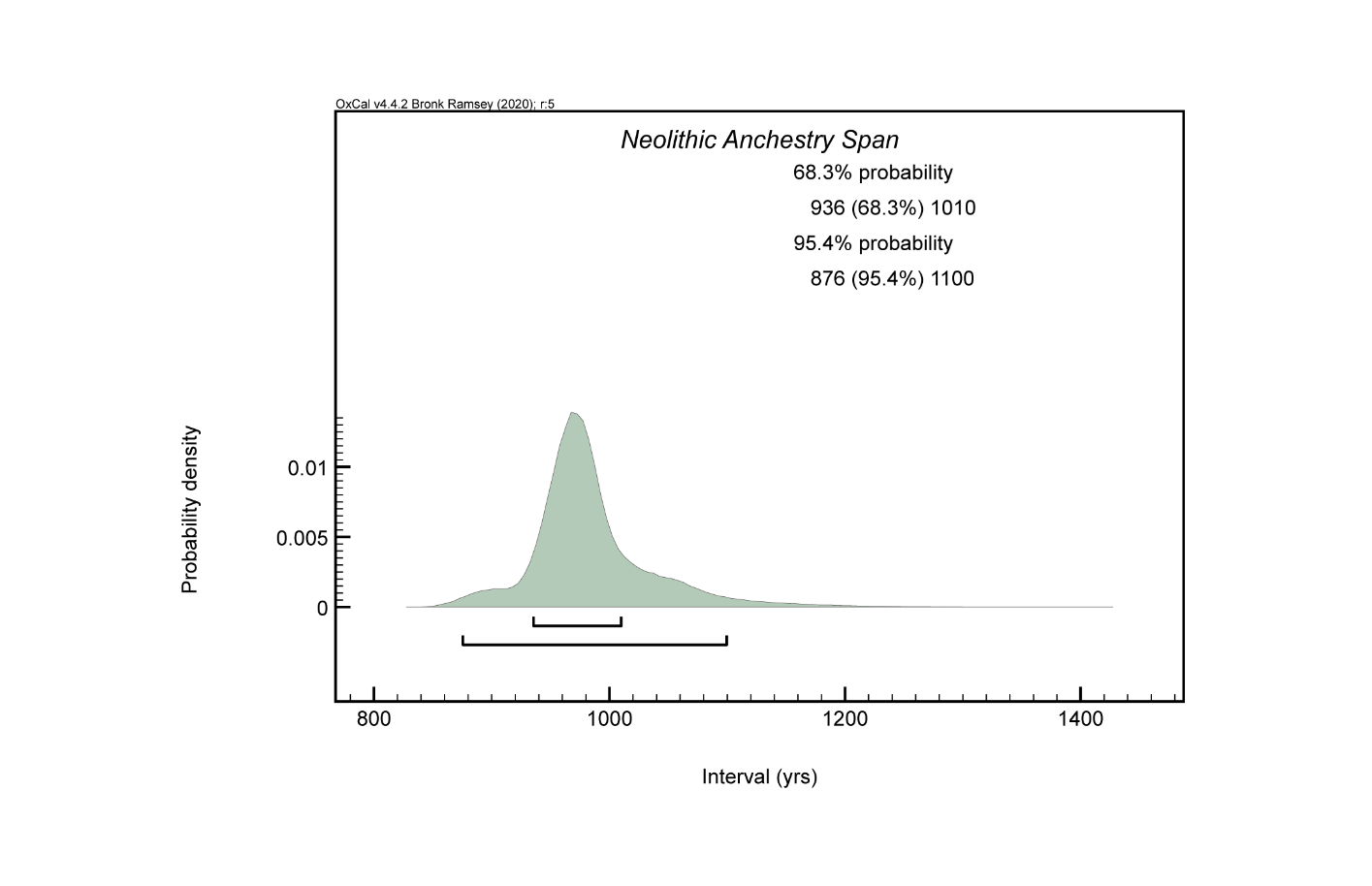
Figure S3.6. Duration of Neolithic ancestry in Denmark.**

References

1. [Buck, C. E., Kenworthy, J. B., Litton, C. D. & Smith, A. F. M. Combining archaeological and radiocarbon information: a Bayesian approach to calibration. *Antiquity* **65**, 808–821 (1991).](http://paperpile.com/b/jM78vL/zGyDr)

2. [Buck, C., Litton, C. D. & Smith, A. F. M. Calibration of Radiocarbon Results pertaining to related archaeological Events. *J. Archaeol. Sci.* **19**, 497–512 (1992).](http://paperpile.com/b/jM78vL/Un1TM)

3. [Ramsey, C. B. Radiocarbon Calibration and Analysis of Stratigraphy: The OxCal Program. *Radiocarbon* **37**, 425–430 (1995).](http://paperpile.com/b/jM78vL/uvanT)

4. [Bronk Ramsey, C. Development of the Radiocarbon Calibration Program OxCal. *Radiocarbon* **43**, 355–363 (2001).](http://paperpile.com/b/jM78vL/cQvCu)

5. [Bayliss, A. & Bronk Ramsey, C. Pragmatic Bayesians: a decade integrating Radiocarbon Dates into chronological Models. in *Tools for constructing Chronologies: Tools for crossing disciplinary Boundaries* (eds. Buck, C. E. & Millard, A. R.) (Springer, 2004).](http://paperpile.com/b/jM78vL/wnTRW)

6. [Bayliss, A., Ramsey, C. B., Plicht, J. van der & Whittle, A. Bradshaw and Bayes towards a Timetable for the Neolithic. *Cambridge Archeological Journal* **17, 1 supplement**, 1–28 (2007).](http://paperpile.com/b/jM78vL/cTuvh)

7. [Bronk Ramsey, C. Deposition Models for chronological Records. *Quat. Sci. Rev.* **27**, 42–60 (2008).](http://paperpile.com/b/jM78vL/EfZgy)

8. [Bayliss, A. Rolling out Revolution: using Radiocarbon dating in Archeology. *Radiocarbon* **51**, 123–147 (2009).](http://paperpile.com/b/jM78vL/JPLgQ)

9. [Bronk Ramsey, C. Dealing with Outliers and Offsets. *Radiocarbon* **51**, 1023–45 (2009).](http://paperpile.com/b/jM78vL/YMkJu)

10. [Bronk Ramsey, C. Bayesian analysis of radiocarbon dates. *Radiocarbon* **51**, 337–360 (2009).](http://paperpile.com/b/jM78vL/SYZmQ)

11. [Bronk Ramsey, C., Dee, M. & Lee, S. Developments in the calibration and modelling of radiocarbon dates. *Radiocarbon* **52**, 953–961 (2010).](http://paperpile.com/b/jM78vL/XCFYH)

12. [Ramsey, C. B. Methods for Summarizing Radiocarbon Datasets. *Radiocarbon* **59**, 1809–1833 (2017).](http://paperpile.com/b/jM78vL/7NG5I)

13. [Bayliss, A. & Whittle, A. What kind of history in prehistory. in *Time and History in Prehistory* (eds. Stella Souvatzi, S., Baysal, A., Baysal, L. & E.) (Routledge, 2018).](http://paperpile.com/b/jM78vL/zMU8w)

14. [Reimer, P., Austin, W. & Bard, E. The IntCal20 Northern Hemisphere Radiocarbon Age Calibration Curve (0–55 cal kBP. *Radiocarbon* **62**, 725–757 (2020).](http://paperpile.com/b/jM78vL/vmPj6)

15. [Karlsberg, A. J. Flexible Bayesian methods for archaeological dating. (University of Sheffield, 2006).](http://paperpile.com/b/jM78vL/FuREb)

16. [Lee, S. & Ramsey, C. Development and Application of the Trapezoidal Model for Archaeological Chronologies. *Radiocarbon* **54**, 107–122 (2012).](http://paperpile.com/b/jM78vL/abevx)

17. [Meadows, J. *et al.* Dietary freshwater reservoir effects and the radiocarbon ages of prehistoric human bones from Zvejnieki, Latvia. *Journal of Archaeological Science: Reports* **6**, 678–689 (2016).](http://paperpile.com/b/jM78vL/YeoGT)

18. [Rose, H. A., Meadows, J. & Bjerregaard, M. High-Resolution Dating of a Medieval Multiple Grave. *Radiocarbon* **60**, 1547–1559 (2018).](http://paperpile.com/b/jM78vL/tsQp3)

#

# 4) Dietary variation in Mesolithic, Neolithic and Bronze Age Denmark

Anders Fischer^1,2,3^ and Karl-Göran Sjögren^1^

^1^Department of Historical Studies, University of Gothenburg, 405 30 Gothenburg, Sweden

^2^Cluster of Excellence ROOTS, Kiel University, Leibnizstr. 3, 24118 Kiel

^3^Sealand Archaeology, Gl. Røsnæsvej 27, 4400 Kalundborg, Denmark

### Introduction

Stable isotope values for carbon (δ^13^C) and nitrogen (δ^15^N) in collagen in genetically characterised human bones and teeth from 100 individuals form the basis for the present overview on dietary variation in early prehistoric Denmark. All isotope values that we comment on in this chapter are released in Allentoft et al. (submitted) where they form part of the supplement chapters on radiocarbon dating and reservoir correction. However, a concise summary of the results are also provided in Supplementary Datas I and II in this study. We analyse data from skeletal material dated c. 10,500-3,000 cal. BP, which according to the local archaeological chronology spans the periods from the Early Mesolithic to the Middle Bronze Age. A tripartite chronological grouping stands out: 1) inland hunter-fisher-gatherers, 2) coastal fisher-hunter-gatherers, and 3) farmers. Integration of the dietary and genetic data for these individuals reveals co-relations that allow for hitherto unattainable insights into the prehistory of the region.

### Material and methods

The dietary stable isotope values discussed in this study were measured on collagen from 40 samples of dentine, 60 of bone, and 12 samples of tooth or bone (some skeletons sampled more than once). 20 of the bone samples derived from the outer parts of petrous bones - excluding the otic capsule, which was reserved for DNA and strontium analyses. The carbon and nitrogen isotope values represent food composition over an interval of time, which generally lasted several years prior to the death of the individual in question[^1,2^](https://paperpile.com/c/IDxvsi/VwUOa+92IUN). A deeper understanding of individual dietary life histories can be reached via isotopic analyses of tooth increments, as will be elucidated in ongoing detailed investigations of skeletons from Vittrup (NEO033), Rødhals Man (NEO645) and Dragsholm Man (NEO962)[^3–5^](https://paperpile.com/c/IDxvsi/2OuJ+HaXh+iz99).

The isotope measurements were mainly produced at the ^14^C Centre at the University of Belfast. They were conducted according to standard protocols[^6^](https://paperpile.com/c/IDxvsi/2cJVh), based on a modified Longin[^7^](https://paperpile.com/c/IDxvsi/jiOWE/?noauthor=1) method [^8^](https://paperpile.com/c/IDxvsi/ESd6N). Ultra-filtering was standard procedure, and measuring uncertainty was within the generally accepted range of ±0.2‰ (1 sd). All are within the acceptable atomic C:N range of 2.9-3.6, and therefore show a low likelihood of diagenesis[^9,10^](https://paperpile.com/c/IDxvsi/4lo56+pRldK). The full assemblage of isotopic measurements is available in Supplementary Data II.

Generally, δ^13^C values inform on the proportion of marine versus terrestrial protein, while δ^15^N values reflect the trophic level from which the proteins were acquired. Dietary interpretation demands reference measurements from potential major protein food sources, temporarily and geographically as closely as possible associated with the humans under study. A large and varied set of such data is available for Danish Mesolithic and Neolithic mammals and fish, but not to the same extent from plants (Allentoft et al. *submitted*)[^3,4,11–19^](https://paperpile.com/c/IDxvsi/zhAm+35FI+KNzo+p9XO+fKYf+piDr+wmWX+qNpF+FmXD+2OuJ+HaXh).

In addition, an understanding of the trophic-level enrichment factor for δ^13^C and δ^15^N values is mandatory for dietary interpretation of our assemblage of human isotope values[^20^](https://paperpile.com/c/IDxvsi/mHYlL). An array of ecological studies on animals indicates that each trophic level shift results in an increase in isotope values of around 1 and 3-5‰, respectively, with variation due to such factors as species, biological age, quantity, quality and composition of diet and physiological stress [^e.g. 21–27^](https://paperpile.com/c/IDxvsi/szIzW+NqmDn+JQdbX+LSTKY+bhfH1+0eFfo+ptdHs/?prefix=e.g.,,,,,,). A δ^15^N increase in the order of 3.5‰ is seen in bone collagen of females and infants from three Danish Mesolithic sites, including mother-and-child burials from Tybrind Vig and Henriksholm-Bøgebakken grave 19[^12,28,29^](https://paperpile.com/c/IDxvsi/35FI+IA7N8+zc57n/?noauthor=0,1,0). In addition, a study based on hair from modern Indian children measured before and after weaning gave the result 3.3‰ [^30^](https://paperpile.com/c/IDxvsi/Xy0RX). However, the actual diets of the studied individuals are not known. More direct measurements on human tissues suggest somewhat higher offsets. A compilation of studies based on present-day human hair samples suggests enrichment around 5.5‰ [^31^](https://paperpile.com/c/IDxvsi/9DrPu). Moreover, a controlled human diet study, based on red blood cell samples, suggests an enrichment factor as high as c. 6‰ [^32^](https://paperpile.com/c/IDxvsi/01MDg).

Application of these different enrichment values will lead to differing interpretations of the human diet. The use of a relatively high δ^15^N enrichment factor will suggest lower proportions of fish consumption and probably also higher intake of plant proteins. Further complications in dietary interpretation result from the raised nitrogen values observed in local early prehistoric cereals[^17,19,33,34^](https://paperpile.com/c/IDxvsi/Rxdep+nPtM0+wmWX+FmXD). These raised values are due to manuring that also affected the δ^15^N levels of domestic animals, which grazed on manured fields. It is beyond the scope of the present supplement to discuss in detail the consequences of these matters to human dietary isotope values, and we shall therefore satisfy noting that there is solid knowledge on widespread fishing activity (and consequently fish consumption) for most of the Mesolithic, Neolithic and Bronze ages [^e.g. 35–43^](https://paperpile.com/c/IDxvsi/T2Sfk+WynKC+tpUaL+Kkxcn+OCTMU+OA3wX+iNF7L+xk9Nd+qIhA2/?prefix=e.g.,,,,,,,,&locator=,,,124,,,,,).

### Dietary variation versus cultural and genetic history

An overview of carbon and nitrogen isotope variation over time among humans from Denmark is given in Extended Data Fig. 6 in the main text (and reproduced below for convenience). A very dominant division is observed c. 5,900 cal. BP, which is the time when domesticates, pottery of Neolithic manufacture and immigrants of Anatolian ancestry turned up in the region[^44,45^](https://paperpile.com/c/IDxvsi/kXTgV+HPIV7) (see discussion in main text).

*Rødhals Man* (NEO645) is coeval to this watershed episode. He is the latest dated individual in our assemblage of Danish humans who combines a dominantly marine dietary isotope signature with a western European hunter-gatherer ancestry[^4^](https://paperpile.com/c/IDxvsi/HaXh). Most noteworthy, *Dragsholm Man* (NEO962) of nearly the same date is also of local hunter-gatherer ancestry, even though his bone collagen dietary signature is that of a farmer (Figs. S4.1 and S4.2)[^5,46^](https://paperpile.com/c/IDxvsi/WmeF+iz99).

Figures S4.1 and S4.2 show a general development throughout the Mesolithic towards an increase in both δ^13^C and δ^15^N values. The latter trend results from a growing importance of protein from high trophic levels, common in aquatic food chains, whereas the former reflects an increase in consumption of marine protein. Both trends correlate with the rise in sea-level that changed the geographic character of the present-day Danish area from inland to archipelago ^cf. 47,67^.


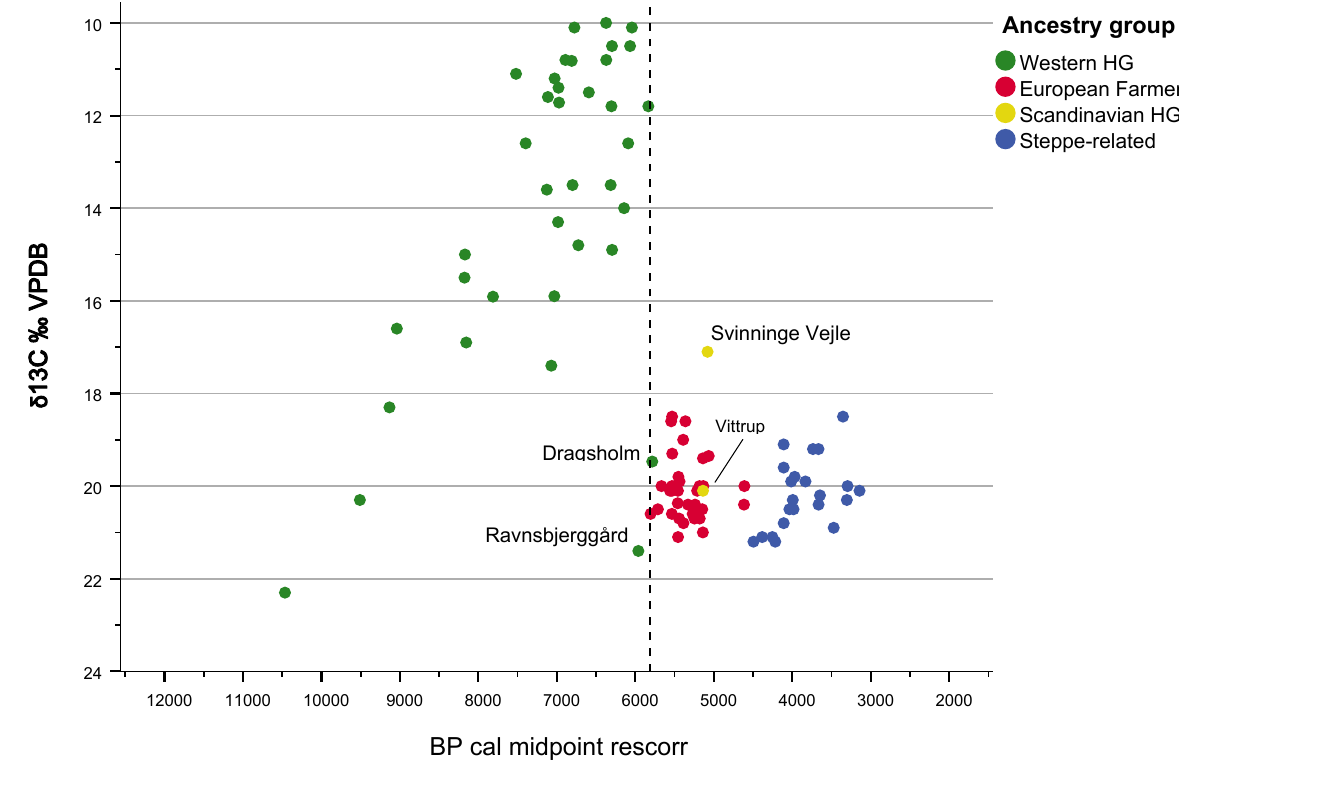


**Figure S4.1. δ^13^C values in Danish early prehistoric human bone and dentine, classed by genomic signature.** A fundamental dietary and genetic change is seen at the transition from the Mesolithic to the Neolithic c. 5900 cal PC (dashed line). Four anomalous individuals are marked. Data from SI Table II.

*
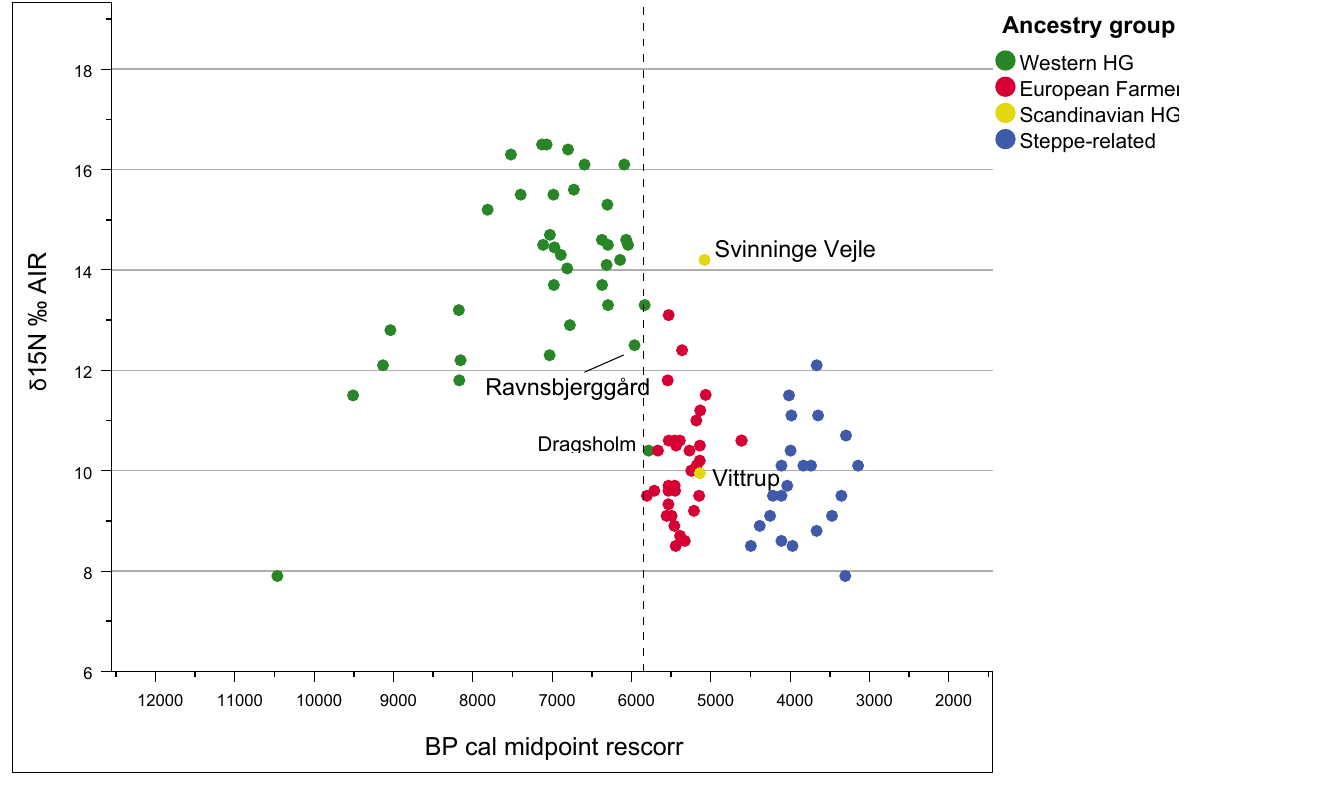
*

**Figure S4.2. δ^15^N values in Danish humans of Early Mesolithic to Middle Bronze Age date, classed by genomic signature**. Four dietary isotopically anomalous individuals are marked. Data from Supplementary Data II.

### Mesolithic food composition

The earliest Danish individual of the present study, *Koelbjerg* (c. 10,500 cal. BP, NEO254), has remarkably low isotopic values. These may be typical of the period, since similar values are known from the second earliest human skeletal remains available from Denmark (Tømmerupgård)[^12^](https://paperpile.com/c/IDxvsi/35FI). They can reflect a protein diet based on terrestrial plants and game in combination with freshwater sources. The terrestrial meat could, for instance, derive from elk, the bones of which are frequently found in local bogs and settlements of the period[^12,15,48,49^](https://paperpile.com/c/IDxvsi/eX18O+35FI+miant+fKYf). Evidence for freshwater food is seen in the period’s many barbed bone points from fish spears found in the lakes of the region[^50,51^](https://paperpile.com/c/IDxvsi/gZ1TJ+RGd9N). The uncertainty as to trophic level enrichment, outlined above, complicates estimation of the relative importance of freshwater sources in Early Mesolithic diet[^12^](https://paperpile.com/c/IDxvsi/35FI), which is therefore not included in this short supplementary.

The much higher δ^15^N values of all other Mesolithic individuals indicate a considerable dependence on higher-level aquatic protein sources. The low δ^13^C values among the individuals from the early and middle part of this period suggest that initially the nutrients of the wet element were of freshwater or highly brackish origin. From c. 9,000 to 5,900 cal. BP, the general trend was an increase in the proportion of proteins from genuinely marine environments.

One individual clearly stands out dietarily from all other Middle and Late Mesolithic members of our assemblage. It is from the site of *Ravnsbjerggård* in the Åmose Bog (NEO960). The protein food of this human was most likely derived from a mixture of freshwater food (fish, mollusks) and forest game. Similar dietary signatures are found in one other human (Tingbjerggård Vest) and several dogs of roughly the same date and deriving from the same bog ([^12^](https://paperpile.com/c/IDxvsi/35FI) and unpublished data). The lack of a marine signal in their isotopes indicates a radical turn away from a long-existing settlement pattern that included prolonged seasonal stays at fishing sites by the sea[^12,52–54^](https://paperpile.com/c/IDxvsi/uXXBZ+6KhC2+201sg+35FI/?noauthor=0,1,1,0). The precise absolute age of these humans and dogs cannot presently be established due to problems in estimating freshwater reservoir effect on their radiocarbon dates (Supplementary Note 3). According to the relatively conservative estimate of the effect applied in Figs. S4.1 and S4.2 (250 years), they belong to a time shortly before the arrival of farming and immigrant farmers to the region. A larger correction factor (still within realistic limits) will make them coeval with the first farming societies of the region. Genetically the Ravnsbjerggård individual is of local hunter-gatherer ancestry. Our preliminary interpretation is that this person, as well as the chronologically and isotopically closely related humans and dogs from the Aamose, represent a social group that gave up its coastal territory – possibly due to impact (violence/diseases) from immigrant farmers.

As to vegetable ingredients in diet, our collagen-based isotope analyses have little to contribute. We know such components were on the menu ^e.g.^ [^28^](https://paperpile.com/c/IDxvsi/IA7N8), but their generally low protein content prevents them from leaving a clear signature. The relative importance to energy supply of carbohydrates and vegetable fats can, however, to some extent be resolved by modelling[^31, cf. 55,56^](https://paperpile.com/c/IDxvsi/Holri+9DrPu+9Vvr8/?prefix=cf.,,&noauthor=0,1,0).

### Neolithic and Bronze Age food composition

The stable isotope values seen in Figs. S4.1 and S4.2 imply a primarily terrestrial diet for nearly all of our Neolithic and Bronze Age individuals. Further nuances to this may, however, be added. Thus, other sources tell that domestic herbivores contributed significantly to the diet of those days’ inhabitants of Denmark in terms of meat as well as milk [^e.g. 57–60^](https://paperpile.com/c/IDxvsi/uOlmQ+MLPOV+TtErf+qYLUd/?prefix=e.g.,,,).

The δ^13^C values centre around -20‰, with a variation from c. -21‰ to -18‰. This would suggest a moderate intake of protein from marine sources, ranging from 0% up to some 20% - in accordance with the frequent observations of large fish weirs of Neolithic date along the coeval coasts[^61–63^](https://paperpile.com/c/IDxvsi/6AjDQ+h1xrv+W6Vth). To what extent freshwater sources contributed to diet can be estimated by modelling, as is the case with the proportion of cereal versus terrestrial animal protein [^cf. 31,64^](https://paperpile.com/c/IDxvsi/9DrPu+LauEm/?prefix=cf.,).

Among the 61 Neolithic and Bronze Age individuals, only one deviates significantly from the others in terms of dietary isotope values (Figs. S4.1 and S4.2). This is the individual from Svinninge Vejle (c. 5,100 cal. BP; NEO898). A large proportion of the protein intake of this person certainly derived from the sea, and his combination of δ^13^C and δ^15^N values suggests a protein diet dominated by marine mammals such as seals. This human also has a deviating genetic profile that is closely related to individuals of Neolithic age from eastern Sweden^3^. The latter are associated with the archaeological complex termed Pitted Ware Culture[^65^](https://paperpile.com/c/IDxvsi/xWTPf), for which seals contributed importantly to diet [^e.g. 66^](https://paperpile.com/c/IDxvsi/1D9Yl/?prefix=e.g.). Our genetic and dietary analyses thus agree in pointing out this individual as a foreigner in Neolithic Denmark.

Genetically *Vittrup Man* (NEO33) was of the same foreign ancestry as the Svinninge Vejle individual. This had no bearing on the isotopic values of his bone collagen, however (Figs. S4.1 and S4.2). His external background is nonetheless supported via measurements of strontium isotopes (Supplementary Note 5).

### References

1. [Hedges, R. E. M., Clement, J. G., David, C., Thomas, L. & O’Connell, T. C. Collagen Turnover in the Adult Femoral Midshaft: Modelled from Anthropogenic Radiocarbon Tracer Measurements. *Am. J. Phys. Anthropol.* **133**, 808–816 (2007).](http://paperpile.com/b/IDxvsi/VwUOa)

2. [Jørkov, M. L. S., Heinemeier, J. & Lynnerup, N. The petrous bone – a new sampling site for identifying early dietary patterns in stable isotope studies. *Am. J. Phys. Anthropol.* **138**, 199–209 (2009).](http://paperpile.com/b/IDxvsi/92IUN)

3. .[Fischer, A. *et al.* Vittrup Man - A genetic foreigner in Neolithic Denmark.](http://paperpile.com/b/JT7E3Y/yPiMd) A combined biomolecular, archaeological and anthropological study (submitted to PlosOne).

4. [Fischer, A. *et al.* Rødhals Man at the end of the Mesolithic world](http://paperpile.com/b/IDxvsi/HaXh) (*in prep.*)[.](http://paperpile.com/b/IDxvsi/HaXh)

5. [Fischer, A. *et al.* Dragsholm Man – jumping the gap between the Mesolithic and the Neolithic worlds](http://paperpile.com/b/IDxvsi/iz99) (*in prep.*)[.](http://paperpile.com/b/IDxvsi/iz99)

6. [Reimer, P., Hoper, S., MacDonald, J., Reimer, R. & Thompson, M. *Laboratory Protocols used for AMS Radiocarbon Dating at the 14CHRONO Centre*. (2015).](http://paperpile.com/b/IDxvsi/2cJVh)

7. [Longin, R. New method of collagen extraction for radiocarbon dating. *Nature* **230**, 241–242 (1971).](http://paperpile.com/b/IDxvsi/jiOWE)

8. [Brown, T. A., Nelson, D. E., Vogel, J. S. & Southon, J. R. Improved collagen extraction by modified Longin method. *Radiocarbon* **30**, 171–177 (1988).](http://paperpile.com/b/IDxvsi/ESd6N)

9. [Ambrose, S. H. & DeNiro, M. J. The isotopic ecology of East African mammals. *Oecologia* **69**, 395–406 (1986).](http://paperpile.com/b/IDxvsi/4lo56)

10. [van Klinken, G. J. Bone Collagen Quality Indicators for Palaeodietary and Radiocarbon Measurements. *J. Archaeol. Sci.* **26**, 687–695 (1999).](http://paperpile.com/b/IDxvsi/pRldK)

11. [Noe-Nygaard, N., Price, T. D. & Hede, S. U. Diet of aurochs and early cattle in southern Scandinavia: evidence from 15N and 13C stable isotopes. *J. Archaeol. Sci.* **32**, 855–871 (2005).](http://paperpile.com/b/IDxvsi/zhAm)

12. [Fischer, A. *et al.* Coast–inland mobility and diet in the Danish Mesolithic and Neolithic: evidence from stable isotope values of humans and dogs. *J. Archaeol. Sci.* **34**, 2125–2150 (2007).](http://paperpile.com/b/IDxvsi/35FI)

13. [Fischer, A., Gotfredsen, A. B., Meadows, J., Pedersen, L. & Stafford, M. The Rødhals kitchen midden – marine adaptations at the end of the Mesolithic world. *Journal of Archaeological Science: Reports* **39**, 103102 (2021).](http://paperpile.com/b/IDxvsi/KNzo)

14. [Ritchie, K., Gron, K. J. & Price, T. D. Flexibility and diversity in subsistence during the late Mesolithic: faunal evidence from Asnæs Havnemark. *Danish Journal of Archaeology* (2013) doi:](http://paperpile.com/b/IDxvsi/p9XO)[10.1080/21662282.2013.821792](http://dx.doi.org/10.1080/21662282.2013.821792)[.](http://paperpile.com/b/IDxvsi/p9XO)

15. [Bocherens, H., Hofman-Kamińska, E., Drucker, D. G., Schmölcke, U. & Kowalczyk, R. European bison as a refugee species? Evidence from isotopic data on Early Holocene bison and other large herbivores in northern Europe. *PLoS One* **10**, e0115090 (2015).](http://paperpile.com/b/IDxvsi/fKYf)

16. [Robson, H. K. *et al.* Carbon and nitrogen stable isotope values in freshwater, brackish and marine fish bone collagen from Mesolithic and Neolithic sites in central and northern Europe. *Environ. Archaeol.* **21**, 105–118 (2016).](http://paperpile.com/b/IDxvsi/piDr)

17. [Gron, K. J. & Rowley-Conwy, P. Herbivore diets and the anthropogenic environment of early farming in southern Scandinavia. *Holocene* **27**, 98–109 (2017).](http://paperpile.com/b/IDxvsi/wmWX)

18. [Maring, R. & Riede, F. Possible Wild Boar Management during the Ertebølle Period. A Carbon and Nitrogen Isotope Analysis of Mesolithic Wild Boar from Fannerup F, Denmark. *Environ. Archaeol.* **24**, 15–27 (2019).](http://paperpile.com/b/IDxvsi/qNpF)

19. [Gron, K. J. *et al.* Archaeological cereals as an isotope record of long-term soil health and anthropogenic amendment in southern Scandinavia. *Quat. Sci. Rev.* **253**, 106762 (2021).](http://paperpile.com/b/IDxvsi/FmXD)

20. [Hedges, R. E. M. & Reynard, L. M. Nitrogen isotopes and the trophic level of humans in archaeology. *J. Archaeol. Sci.* **34**, 1240–1251 (2007).](http://paperpile.com/b/IDxvsi/mHYlL)

21. [Schoeninger, M. J. & DeNiro, M. J. Nitrogen and carbon isotopic composition of bone collagen from marine and terrestrial animals. *Geochim. Cosmochim. Acta* **48**, 625–639 (1984).](http://paperpile.com/b/IDxvsi/szIzW)

22. [Post, D. M. Using stable isotopes to estimate trophic position: models, methods, and assumptions. *Ecology* **83**, 703–718 (2002).](http://paperpile.com/b/IDxvsi/NqmDn)

23. [Bocherens, H. & Drucker, D. Trophic level isotopic enrichment of carbon and nitrogen in bone collagen: a case study from recent and ancient terrestrial ecosystems. *International Journal of Osteoarchaeology* **13**, 46–53 (2003).](http://paperpile.com/b/IDxvsi/JQdbX)

24. [Caut, S., Angulo, E. & Courchamps, F. Discriminating factors (Δ15N and Δ13C) in an omnivorous consumer: effect of diet isotopic ratio. *Funct. Ecol.* **22**, 255–263 (2008).](http://paperpile.com/b/IDxvsi/LSTKY)

25. [Beaumont, J., Montgomery, J., Buckberry, J. & Jay, M. Infant mortality and isotopic complexity: New approaches to stress, maternal health, and weaning. *Am. J. Phys. Anthropol.* **157**, 441–457 (2015).](http://paperpile.com/b/IDxvsi/bhfH1)

26. [Webb, E. C., Stewart, A., Miller, B., Tarlton, J. & Evershed, R. P. Age effects and the influence of varying proportions of terrestrial and marine dietary protein on the stable nitrogen-isotope composition of pig bone collagen and soft tissues from a controlled feeding experiment. *Science and technology of Archaeological Research* **2**, (2016).](http://paperpile.com/b/IDxvsi/0eFfo)

27. [Krajcarz, M. T., Krajcarz, M. & Bocherens, H. Collagen-to-collagen prey-predator isotopic enrichment (Δ13C, Δ15N) in terrestrial mammals - a case study of a subfossil red fox den. *Palaeogeogr. Palaeoclimatol. Palaeoecol.* **490**, 563–570 (2018).](http://paperpile.com/b/IDxvsi/ptdHs)

28. [Fischer, A. *et al.* The composition of Mesolithic food – evidence from a submerged settlement on the Argus Bank, Denmark. *Acta Archaeol.* **78**, 163–178 (2007).](http://paperpile.com/b/IDxvsi/IA7N8)

29. [Brinch Petersen, E. Diversity of Mesolithic Vedbaek. *Acta Archaeol.* **86**, 7–13 (2015).](http://paperpile.com/b/IDxvsi/zc57n)

30. [Dailey-Chwalibog, T. *et al.* Weaning and stunting affect nitrogen and carbon stable isotope natural abundances in the hair of young children. *Science Reports* **10**, 2522 (2020).](http://paperpile.com/b/IDxvsi/Xy0RX)

31. [Fernandes, R., Grootes, P., Nadeau, M. J. & Nehlich, O. Quantitative Reconstruction of a Neolithic Population using a Bayesian Mixing Model (FRUITS): The Case Study of Ostorf (Germany. *Am. J. Phys. Anthropol.* **158**, 325–340 (2015).](http://paperpile.com/b/IDxvsi/9DrPu)

32. [O’Connell, T. C., Kneale, C. J., Tasevska, N. & Kuhnle, G. G. C. The diet-body offset in human nitrogen isotopic values: a controlled dietary study. *Am. J. Phys. Anthropol.* **149**, 426–434 (2012).](http://paperpile.com/b/IDxvsi/01MDg)

33. [Bogaard, A., Heaton, T. H. E., Poulton, P. & Merbach, I. The impact of manuring on nitrogen isotope ratios in cereals: archaeological implications for reconstruction of diet and crop management practices. *J. Archaeol. Sci.* **34**, 335–343 (2007).](http://paperpile.com/b/IDxvsi/Rxdep)

34. [Kanstrup, M., Holst, M. K., Jensen, P. M., Thomsen, I. K. & Christensen, B. T. Searching for long-term trends in prehistoric manuring practice. δ15N analyses of charred cereal grains from the 4th to the 1st millennium BC. *J. Archaeol. Sci.* **51**, 115–125 (2014).](http://paperpile.com/b/IDxvsi/nPtM0)

35. [Richter, J. *Faunal remains from Ulkestrup Lyng Øst. A hunters dwellingplace*. vol. 7 141–177 (Det Kongelige Nordiske Oldskriftselskab, 1982).](http://paperpile.com/b/IDxvsi/T2Sfk)

36. [Andersen, S. H. Coastal adaptation and marine exploitation in Late Mesolithic Denmark – with special emphasis on the Limfjord region. in *Man and Sea in the Mesolithic. Coastal settlement above and below present sea level* (ed. Fischer, A.) 41–66 (1995).](http://paperpile.com/b/IDxvsi/WynKC)

37. [Pedersen, L. 7000 years of fishing: stationary fishing structures in the Mesolithic and afterwards. in *Man and Sea in the Mesolithic. Coastal settlement above and below present sea level* (ed. Fischer, A.) 75–86 (1995).](http://paperpile.com/b/IDxvsi/tpUaL)

38. [Jensen, J. Danmarks Oldtid. Bronzealder 2000-500 f.Kr. (2002).](http://paperpile.com/b/IDxvsi/Kkxcn)

39. [Berntsson, A. Två män i en båt – om människans relation till havet i bronsåldern. *Report series* (2005).](http://paperpile.com/b/IDxvsi/OCTMU)

40. [Fischer, A. Coastal fishing in Stone Age Denmark: evidence from below and above the present sea level and from human bones. *Shell Middens in Atlantic Europe* **30**, 54–69 (2007).](http://paperpile.com/b/IDxvsi/OA3wX)

41. [Enghoff, I. B. *Regionality and biotope exploitation in Danish Ertebølle and adjoining periods*. Scientia Danica. Serie B, Biologica **1**, (2011).](http://paperpile.com/b/IDxvsi/iNF7L)

42. [Enghoff, I. B. Herring and Cod in Denmark. in *Cod and Herring - the archaeology and history of medieval sea fishing* (eds. Barret, J. H. & Orton, D. C.) 133–155 (Oxbow Books, 2016).](http://paperpile.com/b/IDxvsi/xk9Nd)

43. [Pleuger, S. & Makarewicz, C. A. Exploitation of marine fish by Pitted Ware groups at Kainsbakke and Kirial Bro. in *The Pitted Ware Culture on Djursland. Supra-regional significance and contacts in the Middle Neolithic of southern Scandinavia* (ed. Klassen, L.) 341–370 (Aarhus University Press, 2020).](http://paperpile.com/b/IDxvsi/qIhA2)

44. [Fischer, A. Food for feasting. in *The Neolithisation of Denmark: 150 years of debate (eds Fischer, A. & Kristiansen, K.)* 343–393 (2002).](http://paperpile.com/b/IDxvsi/kXTgV)

45. [Sørensen, L. *From hunter to farmer in Northern Europe: Migration and adaptation during the Neolithic and Bronze Age*. Acta Archaeologica **85**, (2014).](http://paperpile.com/b/IDxvsi/HPIV7)

46. [Price, T. D. *et al.* New Information on the Stone Age Graves at Dragsholm, Denmark. *Acta Archaeol.* **78**:**2**, 193–219 (2007).](http://paperpile.com/b/IDxvsi/WmeF)

47. [Fischer, A. & Petersen, P. V. A sea of archaeological plenty. in *Oceans of Archaeology* (eds Fischer, A. & Pedersen, L.) 68–83 (Højbjerg, 2018).](http://paperpile.com/b/IDxvsi/J55td)

48. [Fischer, A. At the border of human habitat. The Late Palaeolithic and Early Mesolithic in Scandinavia. *Acta Archaeologica Lundensia* **8**, 157–176 (1996).](http://paperpile.com/b/IDxvsi/eX18O)

49. [Aaris-Sørensen, K. *Diversity and dynamics of the mammalian fauna in Denmark throughout the last glacial-interglacial cycle, 115-0 kyr BP*. Fossils and Strata 57 57, 1-59 ( 2009).](http://paperpile.com/b/IDxvsi/miant)

50. [Andersen, K. *Stenalderbebyggelsen i Den vestsjællandske Åmose*. (Fredningsstyrelsen, 1983).](http://paperpile.com/b/IDxvsi/gZ1TJ)

51. [Jensen, T. Z. T. *et al.* An integrated analysis of Maglemose bone points reframes the Early Mesolithic of Southern Scandinavia. *Sci. Rep.* **10**, 17244 (2020).](http://paperpile.com/b/IDxvsi/RGd9N)

52. [Fischer, A. People and the sea - settlement and fishing along the mesolithic coasts. in *The Danish Storebælt Since the Ice Age--Man, Sea and Forest* (eds Pedersen, L., Fischer, A. & Aaby, B.) 63-77. (*Copenhagen: A/S Storebaelt Fixed Link*,1997).](http://paperpile.com/b/IDxvsi/uXXBZ)

53. [Fischer, A. Trapping up the rivers, trading across the sea. in *Mesolithic on the Move. Papers presented at the Sixth International Conference on the Mesolithic in Europe, Stockholm 2000* (ed. Larsson, L. et al.) 393–401 (2003).](http://paperpile.com/b/IDxvsi/6KhC2)

54. [Fischer. *Tissø og Åmoserne som trafikforbindelse og kultsted i stenalderen, in Tissø og Åmoserne – kulturhistorie og natur* (ed. Pedersen, L.) 27–44 (Historisk samfund for Holbæk Amt, Kalundborg, 2004).](http://paperpile.com/b/IDxvsi/201sg)

55. [Fernandes, R., Millard, A. R., Brabec, M., Nadeau, M.-J. & Grootes, P. Food reconstruction using isotopic transferred signals (FRUITS): a Bayesian model for diet reconstruction. *PLoS One* **9**, e87436 (2014).](http://paperpile.com/b/IDxvsi/Holri)

56. [Sjögren, K.-G. Modeling Middle Neolithic Funnel Beaker diet on Falbygden, Sweden. *Journal of Archaeological Science: Reports* **12**, 295–306 (2017).](http://paperpile.com/b/IDxvsi/9Vvr8)

57. [Rowley-Conwy, P. Mellemneolitisk økonomi i Danmark og Sydengland. *Kuml* 77–111 (1984).](http://paperpile.com/b/IDxvsi/uOlmQ)

58. [Nyegaard, G. Faunalevn fra yngre stenalder på øerne syd for Fyn. in *Yngre stenalder på øerne syd for Fyn. Langelands Museum, Rudkøbing* (ed. Skaarup, J.) 426–466 (1985).](http://paperpile.com/b/IDxvsi/MLPOV)

59. [Makarewicz, C. & Pleuger, S. Herder-hunter-fishers and agricultural contacts. Zooarchaeological perspectives on pitted ware animal exploitation strategies from Djursland. in *The Pitted Ware Culture on Djursland. Supra-regional significance and contacts in the Middle Neolithic of southern Scandinavia* (ed. Klassen, L.) 279–339 (Aarhus University Press, Aarhus, 2020).](http://paperpile.com/b/IDxvsi/TtErf)

60. [Robson, H. K. *et al.* Organic residue analysis of Early Neolithic ‘bog pots’ from Denmark demonstrates the processing of wild and domestic foodstuffs. *Journal of Archaeological Science Reports* **36**, (2021).](http://paperpile.com/b/IDxvsi/qYLUd)

61. [Pedersen, L. Eelers in Danish waters – interaction between men and their environment over 8000 years. in *Ancient Maritime Communities and the Relationship between People and Environment along the European Atlantic Coasts. BAR International Series 2570* (ed. Daire, M.-Y. et al.) 163–173 (2013).](http://paperpile.com/b/IDxvsi/6AjDQ)

62. [Pedersen, L., Fischer, A. & Gregory, D. J. Fletværket ved Nekselø – skovdrift og storstilet fiskeri i bondestenalderen (English summary: The Wickerwork off Nekselø – forestry and large-scale fisheries in the Neolithic. *Nationalmuseets Arbejdsmark* 134–145 (2017).](http://paperpile.com/b/IDxvsi/h1xrv)

63. [Jensen, L. E. *et al.* Syltholmudgravningerne – jagten på stenalderens jægere, fiskere og bønder i et druknet landskab. *Aarbøger for Nordisk Oldkyndighed og Historie* **2016** 33-61 (2018).](http://paperpile.com/b/IDxvsi/W6Vth)

64. [Sjögren, K.-G. *et al.* Early Neolithic human bog finds from Falbygden, western Sweden: *Journal of Neolithic Archaeology* 97–126 (2017).](http://paperpile.com/b/IDxvsi/LauEm)

65. [Coutinho, A. *et al.* The Neolithic Pitted Ware culture foragers were culturally but not genetically influenced by the Battle Axe culture herders. *American Journal of* *Phys Anthropol* (2020).](http://paperpile.com/b/IDxvsi/xWTPf)

66. [Eriksson, G. Part-time farmers or hard-core sealers? Västerbjers studies by means of stable isotope analysis. *Journal of Anthropological Archaeology* **23**, 135–162 (2004).](http://paperpile.com/b/IDxvsi/1D9Yl)

67. Astrup, P. M. Sea-level change in Mesolithic southern Scandinavia. Jutland Archaeological Society, Højbjerg (2018).

# 5) Strontium analysis of Danish samples

T. Douglas Price^1^ and Karl-Göran Sjögren^2^

^1^ Laboratory for Archaeological Chemistry, University of Wisconsin-Madison, Madison, United States of America

^2^ Department of Historical Studies, University of Gothenburg, 405 30 Gothenburg, Sweden

### Introduction

Studies of human movement in the past have traditionally relied on exotic artefacts as a proxy for people. But artefacts can be copied, stolen, traded, or gifted and may not represent the actual movement of people. In the last 25 years or so, isotopic provenancing of human remains has become common practice in the investigation of burial populations and provides a direct way to look at human mobility[^1^](https://paperpile.com/c/Px0md5/k4RPt). Isotopes of oxygen, strontium, and lead have been used in such studies. A number of studies have been completed in Denmark[^2–8^](https://paperpile.com/c/Px0md5/zxHW3+4wKmN+JXzJK+rwMJS+m9B9j+ne8Nl+5wbW7/?noauthor=0,0,0,0,0,1,1).

Strontium isotope analysis provides a robust means for examining past mobility. Strontium moves into humans from rocks and sediments through the food chain[^1,9,10^](https://paperpile.com/c/Px0md5/ucNUd+JfGoo+k4RPt/?noauthor=0,0,0) and is found primarily in the skeleton. Enamel forms during early childhood and has the strontium isotope ratio of the food consumed by the mother and during the first years of life. Enamel is largely inert. The strontium isotope ratio in the enamel usually remains unchanged during life and after death. The enamel thus can provide a chemical signal of place of birth. If an individual moved to a new location or was buried in a new place, the enamel isotope ratio may differ from the new location, allowing the designation of that individual as non-locally born. Because the same strontium isotope ratio can characterise several locations, it is difficult to determine the precise place of origin.

The strontium isotope ratio (^87^Sr/^86^Sr) varies among different kinds of rocks, based on their age and composition. The heavier isotope (^87^Sr) is formed by the radioactive decay of rubidium-87. Thus, older rocks and sediments with more rubidium have higher ^87^Sr/^86^Sr values, while younger materials with less rubidium are at the opposite end of the range with lower ratios [^e.g. 11^](https://paperpile.com/c/Px0md5/4RNC0/?prefix=e.g.). The proportion of ^87^Sr varies in the terrestrial ecosystem, but averages around 7% of total strontium; ^86^Sr is about 10%. Their ratio normally varies from about 0.700 in rocks with low Rb to 0.730 and much higher in high-Rb rocks that are billions of years old. Most measurements of human enamel fall in the range of 0.705 to 0.735.

Strontium isotope analysis for information on prehistoric residential mobility requires samples of uncremated dental enamel. Powder for isotopic analysis is collected from the teeth by first burying the area to be sampled to remove possible surface contamination and then extracting a cusp or fragment. The powder is weighed and submitted for measurement. For this study, measurement of ^87^Sr/^86^Sr in the powder was done in the Geochronology and Isotope Geochemistry Laboratory (Dept. of Geological Sciences, University of North Carolina- Chapel Hill). Samples are dissolved in nitric acid and the strontium fraction purified by ion selective chromatography (Eichrom Sr resin) prior to analysis by TIMS on a VG Sector 54 mass spectrometer run in dynamic mode. Internal precision in the laboratory is consistently around 0.0007% standard error (or 1σ = 0.00006 in the ratio of a particular sample). Long-term, repeated measurements of SRM-987 are around 0.710260—an acceptable difference from the recognized value of 0.710250—and raw sample values from individual runs are standardised to the recognized value of SRM-987.

Levels of strontium isotopes in human enamel and bone may vary from geological background for a number of reasons that include differential weathering of minerals in rock, atmospheric dust, and the deposition of eolian, alluvial, or glacial sediments on top of bedrock geology. Complex geological areas may have several different sources of ^87^Sr/^86^Sr contributing to human diets. Coastal populations are impacted by other phenomena. Marine foods, for example, have a constant strontium isotope ratio on average of 0.7092. The same ratio, 0.7092, may also be introduced by salt spray and rainfall in coastal areas. For these and other reasons, it is important to measure bioavailable levels of ^87^Sr/^86^Sr to ascertain local strontium isotope ratios[^12^](https://paperpile.com/c/Px0md5/l2suZ).

Bioavailable strontium isotope ratios are those ratios actually available in the food chain. The bioavailable strontium isotopic signal (or baseline) of the place of burial can be determined in several ways: in human bone from the individuals whose teeth were analysed, from the bones of other humans or archaeological fauna at the site, or from modern fauna, water, soil extracts or vegetation in the vicinity[^13^](https://paperpile.com/c/Px0md5/rc8lP). This baseline information on isotope values across an area needs to be obtained in order to make useful and reliable statements about the origins of the human remains under study. Some baseline information is available for Denmark[^14,15^](https://paperpile.com/c/Px0md5/T4tao+ZE4LP) with a mean of 0.7092 and a range largely between 0.7078 and 0.7108. Samples for these baselines were taken at a large scale and, because of variation in the surface deposits across Denmark, measurement of baseline samples at the local level is essential. Local variation has indeed been discovered in some areas with denser sampling[^5,16^](https://paperpile.com/c/Px0md5/rwMJS+sXP1E), and there is an ongoing discussion on the Danish Sr baseline, which may possibly have to be revised[^16^](https://paperpile.com/c/Px0md5/sXP1E). In this study we do not rely on baseline values for our interpretations.

### Results

One of the strengths of our project has been the availability of radiocarbon dates for all samples. Fig. S5.1 is a scatterplot of reservoir corrected age vs. ^87^Sr/^86^Sr value for 81 teeth out of the 100 analysed samples from Denmark (see also Fig 4 in main text). There are few burials from the end of the Mesolithic, and (due to sampling limitations) in our dataset there are also relatively few individuals from the end of the Middle Neolithic from 4500 to 4000 uncal. BP. The plot has several interesting features. A dramatic change can be seen with the start of the Neolithic, as these data include both higher and lower values than before, and are generally somewhat higher. This would indicate a different mobility pattern, with many of the Neolithic individuals originating from more distant areas, or possibly from areas that were not utilised or settled in the Mesolithic. The variability in ^87^Sr/^86^Sr values continues in the later Neolithic as well after 2500 BC.

It has been argued[^17^](https://paperpile.com/c/Px0md5/MG8aL) that the last hunters were more sedentary and the first farmers in prehistoric southern Scandinavia were more mobile and this graph may support such an interpretation. In addition, this pattern is very similar to what has been documented in the British Isles[^18^](https://paperpile.com/c/Px0md5/mGjO8).

Two individuals show values above 0.714, one from the Mesolithic and one from the Neolithic period. These individuals may have originated from the Scandinavian Peninsula. There are three individuals between 0.712 and 0.714, all Neolithic, place of origin difficult to determine. There are also some individuals below 0.709 that may be non-local, but could also come from areas within Denmark, such as Limfjorden.

Carbon isotopes indicate that later Mesolithic diets contained substantial amounts of marine food which would have had an ^87^Sr/^86^Sr value of 0.7092 and kept ^87^Sr/^86^Sr in Mesolithic humans low. Neolithic diets on the other hand did not include large quantities of marine foods (Supplementary Note 4) and imply little or no suppression of ^87^Sr/^86^Sr values. It is therefore surprising that the average ^87^Sr/^86^Sr values for the Neolithic is only slightly higher (Table S5.1).


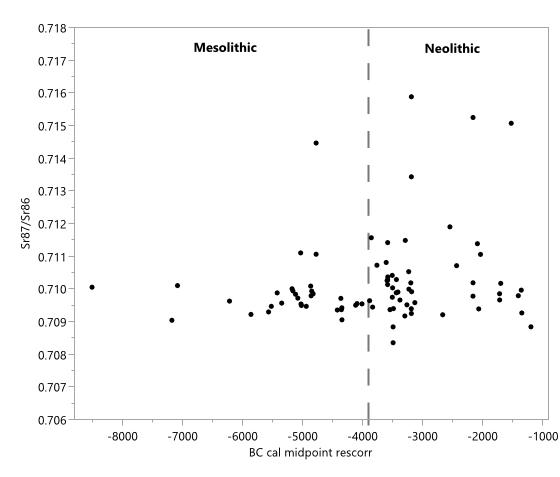


**Figure S5.1. Scatterplot of ^14^C age vs. ^87^Sr/^86^Sr value for 81 analysed samples from Mesolithic and Neolithic Denmark.** The grey vertical line indicates the advent of the Neolithic c. 3900 cal. BC (c. 5,900 cal. BP). Data from Supplementary Table II (See also Fig 4 in the main text).

| **Statistic** | **All Samples** | **Mesolithic** | **Neolithic** |
| --- | --- | --- | --- |
| Mean | 0.710199 | 0.70987 | 0.71040 |
| Standard Deviation | 0.001 | 0.0009 | 0.001 |
| Minimum | 0.70835 | 0.70937 | 0.70835 |
| Maximum | 0.71588 | 0.71446 | 0.71588 |
| Count | 81 | 31 | 50 |

**Table S5.1. Descriptive statistics for ^87^Sr/^86^Sr for all samples (both periods).** Additionally, these are provided for the Mesolithic and Neolithic components of the sample.

Table S5.1 provides descriptive statistics for all Mesolithic and Neolithic samples and for the two components of the sample combined. The very high ^87^Sr/^86^Sr value in one of the Mesolithic samples (0.714) affects the mean and s.d. for the entire Mesolithic group. These values for the Mesolithic recalculated without the high value are 0.70959 ± 0.0004.


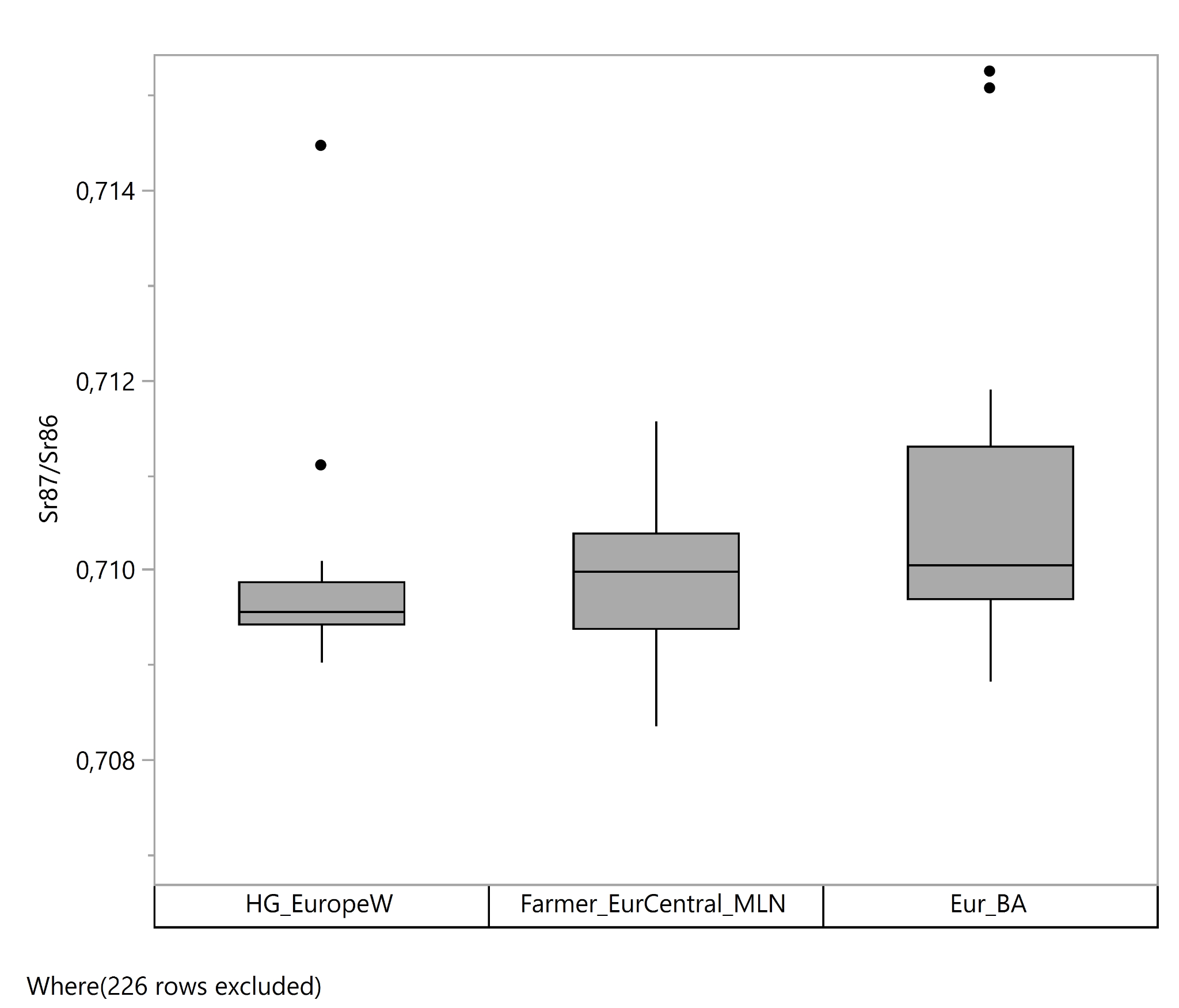


**Figure S5.2. Boxplot of Sr isotope ratios for the main ancestry groups in our Danish dataset. See also figure 4 in the main text.**

Fig. S5.2 shows the distribution of Sr isotope ratios for the Danish samples that could be genetically characterised (Vittrup (NEO33) excluded since it does not belong to one of the major groups). Higher variation as well as slightly higher medians for individuals with Neolithic farmer-related and steppe-related ancestry are suggested by the boxplot. This is supported by nonparametric significance tests. Both Median and Kruskal-Wallis tests suggest overall significant differences between ancestry groups, p=0.0378 resp p= 0.0304.

Pairwise testing between individuals with Mesolithic HG-ancestry and Neolithic farmer-related ancestry gave the following: Median test p=0.0211, Kolmogorov-Smirnov test p=0.0187, Kruskal-Wallis test p=0.0536. Only the K-W test was marginally insignificant. We are confident in concluding that the distribution of Sr isotope values are significantly different from those in the Mesolithic. Pairwise testing between the Eur_BA (Steppe-related ancestry) against the other two ancestry groups also showed significant differences in the distribution of values. Fig. S5.3 shows the spatial distribution of the three ancestry groups, all of which are concentrated in the more easterly parts of Denmark where bone preservation is better. The spatial distribution plots also allows us to evaluate the Sr values in direct comparison with the surface geology. In Denmark, the surficial geology comprises a mosaic of different glacial and post glacial sediments that is the result of sediment dispersal of multiple ice transgression reflecting ice movements from Norway, central Sweden, and the Baltic Sea area. Thus, together with local ice-bedrock interaction potential provides a variable source of different Sr values. While the samples representing each of the three ancestry groups are indeed clustered in the same eastern region, we cannot definitively conclude that the higher Sr-variation we observe in the Neolithic onwards is not a result of those groups simply occupying a wider spectrum of local surface geologies rather than being a signal of higher mobility. This is why we cautiously write in the main text that “*This could suggest that the Neolithic farmers in Denmark occupied and/or consumed food from more diverse landscapes, or were more mobile than the preceding hunter-gatherers*”.


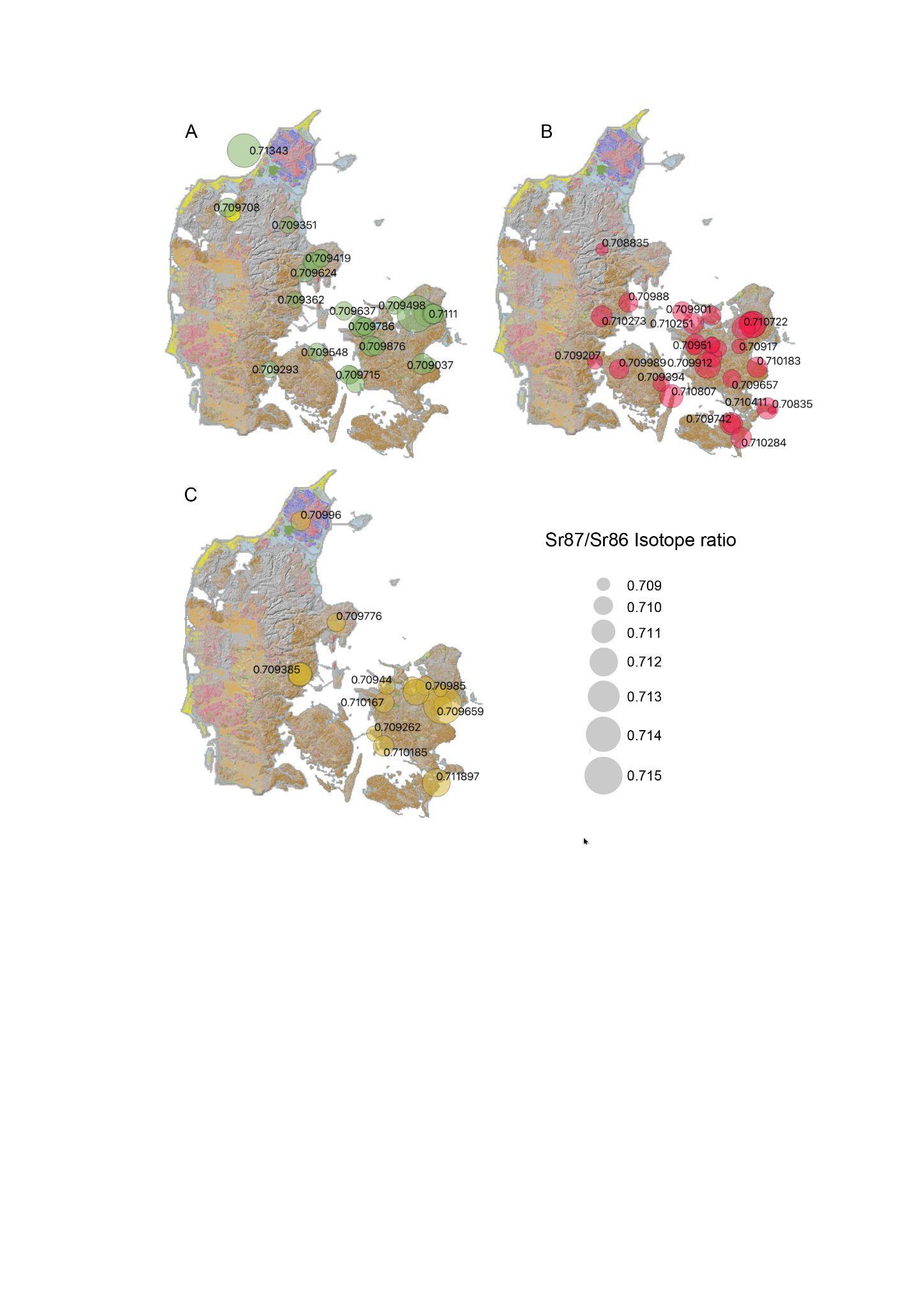


**Figure S5.3. Spatial Sr isotope ratios for the main ancestry groups in our Danish dataset.** A) Mesolithic (HG_Europe_W); B) Early Neolithic (Farmer_EurCentral_MLN); C) Late Neolithic and Early Bronze Age (Eur BA). Modified soil classification as background map from GEUS. Predominate sediments: Brown colours refer to glacial moraine deposits. In eastern Denmark and east coast of Jylland these are rich in carbonates related to the local chalk bedrock. Red and orange colours refer to glacial outwash sediments. Violet colours refer to raised marine deposits and green colours to postglacial organic rich deposits often overlaying moraine deposits. The complex distribution of carbonate rich deposits with high Sr concentrations might influence the presence of bioavailable Sr. See <https://data.geus.dk/pure-pdf/GEUS-R_2021_68_web.pdf> for full legend.

### References

1. [Price, T. D. An Introduction to Isotopic Studies of Ancient Human Remains. *Journal of the North Atlantic* **7**, 71–87 (2015).](http://paperpile.com/b/Px0md5/k4RPt)

2. [Dobat, A. S., Price, T. D., Kveiborg, J., Ilkjær, J. & Rowley-Conwy, P. The four horses of an Iron Age apocalypse - Warhorses from the 3d century weapon sacrifice at Illerup Aadal (Denmark. *Antiquity* **88**, 191–204 (2014).](http://paperpile.com/b/Px0md5/zxHW3)

3. [Frei, K. M., Ulla Mannering, T. D. P. & Iversen, R. B. Strontium isotope investigations of the Haraldskær Woman– a complex record of various tissues. *Archaeosciences, revue d’archéométrie* **39**, 93–101 (2015).](http://paperpile.com/b/Px0md5/4wKmN)

4. [Frei, K. M. *et al.* Mapping human mobility during the third and second millennia BC in present-day Denmark. *PLoS One* **14**, e0219850 (2019).](http://paperpile.com/b/Px0md5/JXzJK)

5. [Klassen, L., Price, T. D., Karl-Göran Sjögren, L. W. & Phillipsen, B. Strontium and lead isotope studies of faunal and human remains from Kainsbakke and Kirial Bro. in *The Pitted Ware Culture on Djursland: Supra-regional significance and contacts in the Middle Neolithic of southern Scandinavia* (ed. Klassen, L.) 407–446 (Museum of Østjylland, 2020).](http://paperpile.com/b/Px0md5/rwMJS)

6. [Price, T. D., Nielsen, J. N., Frei, K. M. & Lynnerup, N. Sebbersund: isotopes and mobility in an 11th - 12th c. *AD Danish churchyard. Journal of Archaeological Science* **39**, 3714–3720 (2012).](http://paperpile.com/b/Px0md5/m9B9j)

7. [Price, T. D. *et al.* Investigation of Human Provenience at the Early Medieval Cemetery of Ndr. Grødbygård, Bornholm, Denmark. *Danish Journal of Archaeology* **1**, 93–112 (2013).](http://paperpile.com/b/Px0md5/ne8Nl)

8. [Price, T. D., Prangsgaard, K., Kanstrup, M., Bennike, P. & Frei, K. M. Galgedil. Isotopic studies of a Viking cemetery on the island of Funen, Denmark, AD 700–1100. *Danish Journal of Archaeology* (2015) doi:](http://paperpile.com/b/Px0md5/5wbW7)[10.1080/21662282.2015.1056634](http://dx.doi.org/10.1080/21662282.2015.1056634)[.](http://paperpile.com/b/Px0md5/5wbW7)

9. Price, T.D. (ed.) [*The Chemistry of Prehistoric Human Bone*. (Cambridge University Press, 1989).](http://paperpile.com/b/Px0md5/ucNUd)

10. [Sillen, A. & Kavanagh, M. Strontium and paleodietary research: A review. *Am. J. Phys. Anthropol.* **25**, 67–90 (1982).](http://paperpile.com/b/Px0md5/JfGoo)

11. [Faure, G. & Mensing, T. M. *Isotopes: Principles and Applications*. (John Wiley & Sons, 2005).](http://paperpile.com/b/Px0md5/4RNC0)

12. [Price, T. D., Burton, J. H. & Bentley, R. A. Characterization of Biologically Available Strontium Isotope Ratios for the Study of Prehistoric Migration. *Archaeometry* **44**, 117–135 (2002).](http://paperpile.com/b/Px0md5/l2suZ)

13. [Maurer, A.-F. *et al.* Preservation vs. anthropogenic contamination of natural bioavailable strontium in Saxony-Anhalt, Germany, with implications for isoscapes in past migration studies. *Sci. Total Environ.* **433**, 216–229 (2012).](http://paperpile.com/b/Px0md5/rc8lP)

14. [Frei, K. M. & Frei, R. The geographic distribution of strontium isotopes in Danish surface waters – a base for provenance studies in archaeology, hydrology and agriculture. *Appl. Geochem.* **26**, 326–340 (2011).](http://paperpile.com/b/Px0md5/T4tao)

15. [Frei, K. M. & Price, T. D. Strontium isotopes and human mobility in prehistoric Denmark. *Archaeol. Anthropol. Sci.* **4**, 103–114 (2012).](http://paperpile.com/b/Px0md5/ZE4LP)

16. [Thomsen, E., Andreasen, R. & Rasmussen, T. L. Homogeneous Glacial Landscapes Can Have High Local Variability of Strontium Isotope Signatures: Implications for Prehistoric Migration Studies. *Frontiers in Ecology and Evolution* **8**, 588318 (2021).](http://paperpile.com/b/Px0md5/sXP1E)

17. [Price, T. D., Larsson, L., Magnell, O. & Boriç, D. Mobility and sedentary behavior among Europe’s last hunters and first farmers. in *Foraging Assemblages: Papers Presented at the Ninth International Conference on the Mesolithic in Europe, Belgrade 2015* (ed. Boriç, D.) (2020).](http://paperpile.com/b/Px0md5/MG8aL)

18. [Neil, S., Evans, J., Montgomery, J. & Scarre, C. Isotopic evidence for residential mobility of farming communities during the transition to agriculture in Britain. *Royal Society Open Science* **3**, 150522 (2016).](http://paperpile.com/b/Px0md5/mGjO8)

# 6) Vegetation and landscape in Post-Glacial Denmark – illustrated using a high-resolution land cover reconstruction (LOVE) from Lake Højby, Northwest Zealand

Morten Fischer Mortensen^1^, Anne Birgitte Nielsen^2^, Mikkel Ulfeldt Hede^3^, Anthony Ruter^4^, Kristian Kristiansen^5^ and Peter Rasmussen^6^

^1^ National Museum of Denmark, Environmental Archaeology and Materials Science, I.C. Modewegsvej, Brede, DK-2800 Kgs. Lyngby, Denmark

^2^ Department of Physical Geography and Ecosystem Science, Lund University, Sölvegatan, Lund, Sweden

^3^ Tårnby Gymnasium og HF, Tejn Alle 5, 2770 Kastrup, Denmark.

^4^ GLOBE Institute, University of Copenhagen, 1350, Copenhagen K, Denmark

^5^ Department of Historical Studies, University of Gothenburg, 405 30 Gothenburg, Sweden

^6^ Section for Environmental Archaeology and Materials Science, The National Museum of Denmark, I.C. Modewegs Vej, Brede, 2800 Copenhagen, Denmark

### The Mesolithic-Neolithic transition: palynological evidence

Vegetation and landscape development in Denmark over the last 15000 years is well documented through a large number of pollen stratigraphic records from lakes, bogs and kettle holes[^1–5^](https://paperpile.com/c/YAZ9jC/usN1l+wsxTk+wh8CP+Eo3cb+tzwlY). The main drivers for vegetation changes through the Late Palaeolithic and Mesolithic periods were climate, soil conditions and the succession of various plant species[^6,7^](https://paperpile.com/c/YAZ9jC/fw3iK+3ckLU). So far, there is no unequivocal evidence of anthropogenic influence on the vegetation on a regional scale before the Neolithic period.

With the introduction of agriculture in Denmark *c. 3900 cal. BC* (5900 cal. BP) humans became a crucial driving force behind vegetation changes. Johannes Iversen was the first to demonstrate this in his groundbreaking publication on the expansion of Neolithic settlement (`landnam´: land-taking) in Denmark[^8^](https://paperpile.com/c/YAZ9jC/PGVXz). With the subsequent studies of Iversen[^9,10^](https://paperpile.com/c/YAZ9jC/Svdhd+OOQkV/?noauthor=1,1) and Troels-Smith[^11,12^](https://paperpile.com/c/YAZ9jC/yrkY5+nuLCz/?noauthor=1,1), the most important features of the Neolithic vegetation development on the fertile soils in eastern Denmark were clarified. First is the classic ‟elm declineʺ, which marks the beginning of the Neolithic in historic vegetation studies. The decline of elm is followed by a brief increase in the frequency of grass (*Poaceae*) and herb pollen, which are then followed by a longer-lasting maximum of birch (*Betula*), which coincides with the first occurrence of the grazing indicator ribwort plantain (*Plantago lanceolata*) and sporadic evidence of grain cultivation (occurrence of cereal pollen grains). Birch then declines, while hazel (*Corylus*) achieves high frequencies in the pollen records until the primary forest taxa reemerge*.*

Iversen originally interpreted the development as a relatively short sequence: 1) first the forest was cleared by burning and the land was cultivated, 2) after a few growing seasons, the soil was nutrient deficient and the fields were abandoned, after which a natural plant succession began. First with birch forest, where the farmers´ cattle grazed 3), but over time the birch was outcompeted by hazel in a natural order towards the re-establishment of the primary forest. However, as pollen records have been ^14^C dated and chronology has improved, we now know that this development extends over nearly a thousand years. Thus, it is not a single cycle of natural succession, but different stages of landscape utilisation and agricultural strategies that are expressed[^13,14^](https://paperpile.com/c/YAZ9jC/5ffiD+1JKpK). The phase division of the landnam period described by Iversen[^15^](https://paperpile.com/c/YAZ9jC/vbjl1/?noauthor=1) is of a general nature and there are significant variations from locality to locality.

To illustrate the vegetation development at the transition from the Mesolithic to the Neolithic in eastern Denmark, we use a new high-resolution and well-dated pollen diagram from Lake Højby, Northwest Zealand, covering the period *5000-2400 cal. BC*[^16^](https://paperpile.com/c/YAZ9jC/JXPV6). In this study, a “Landscape-Reconstruction Algorithm” (LRA[^17,18^](https://paperpile.com/c/YAZ9jC/jdXyn+ICaYO)) is used to reconstruct the vegetation changes in the catchment area of Lake Højby over a period of c. 2500 years. In the LRA, vegetation cover composition of the most common plants with wind-dispersed pollen types within the local area is estimated with the model LOVE (Local Vegetation Estimates[^17^](https://paperpile.com/c/YAZ9jC/jdXyn)). This corrects for differences in pollen productivity and dispersal, as well as for changes in the regional background pollen rain, estimated from the regional vegetation composition, which has previously been estimated with the model REVEALS[^17^](https://paperpile.com/c/YAZ9jC/jdXyn) based on pollen data from six lakes on Zealand (see Fig. S6.1).


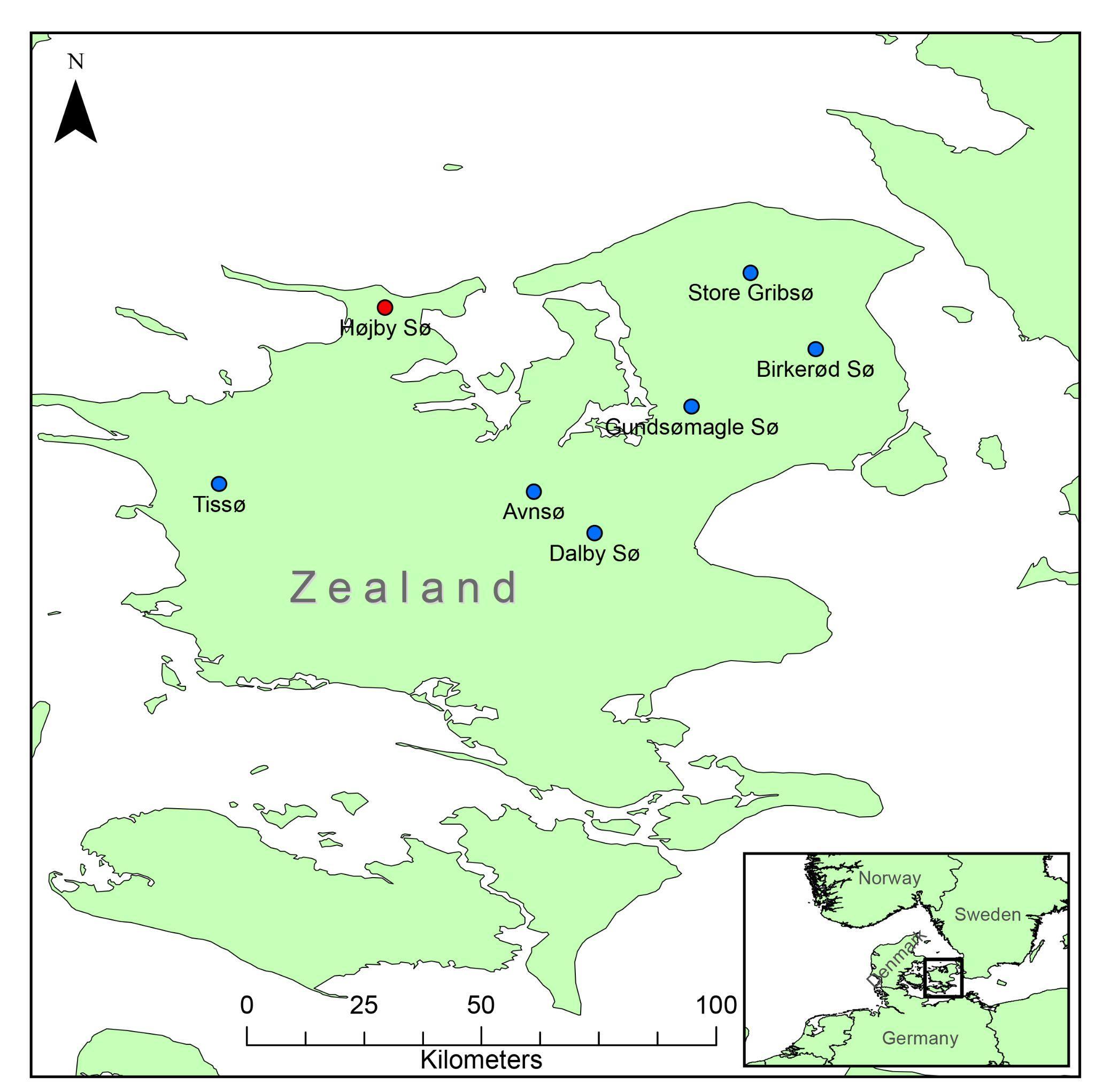


**Figure S6.1. Map showing the location of Lake Højby in Northwest Zealand (red), and the other six lakes (blue).** These were used to estimate the regional vegetation composition with the LRA-REVEALS model.

The development in eastern Denmark will be compared with the development on the sandier and less nutrient-rich soils in western and northern Jutland, where the vegetation development deviates to some extent from the development in eastern Denmark.

#### Mesolithic

A succession of temperate deciduous forests started in Denmark around *7000cal. BC*. This primary forest, often named the Atlantic primeval forest, consisted of a variety of trees, dominated by lime (*Tilia*), elm (*Ulmus*), oak (*Quercus*), alder (*Alnus*) and ash (*Fraxinus*). These shade-tolerant species gradually outcompeted the hazel and pine (*Pinus*) which dominated the older Boreal forests. On the nutrient-rich soils prevalent in eastern Jutland, Funen and Zealand, relatively low pollen proportions of light-demanding herb species have been registered. This indicates that the primary forest was relatively dense, with few and small open areas[^19^](https://paperpile.com/c/YAZ9jC/EkB7z). Modelling of the area distribution from Lake Højby shows that between 85 % and 90 % of the area was covered by primary forest (Fig. S6.2), which is in accordance with previous studies from eastern Denmark on a regional scale[^20^](https://paperpile.com/c/YAZ9jC/OUu9t). The open areas have primarily been concentrated around wetlands and along the coasts.


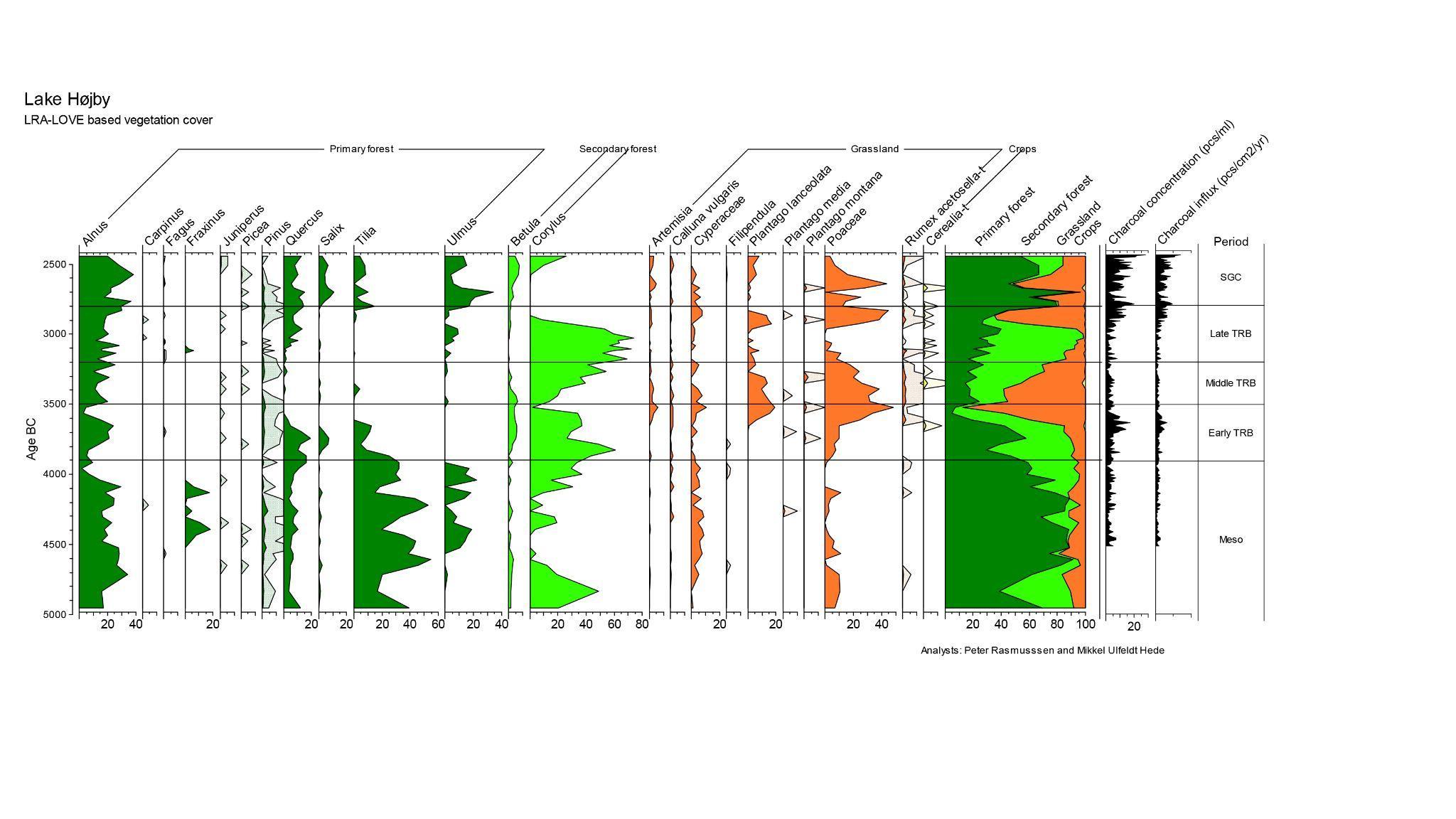


**Figure S6.2. Local vegetation cover percentages around Lake Højby, estimated from pollen assemblages using the LRA-LOVE model.** Charcoal concentration and influx is also shown. Archaeological periods are indicated on the diagram: Meso, Mesolithic; Early TRB, Early Funnelbeaker Culture; Middle TRB, Middle Funnelbeaker Culture; Late TRB, Late Funnelbeaker Culture; SGC, Single Grave Culture.

On the sandier and less fertile soils in western and northern Jutland, the Boreal forest was similarly replaced by temperate deciduous forest of the same tree species as in the east, but the forest maintains a more open character with a field layer of grasses and heather. Modelling of pollen data from Lake Solsø (western Jutland), located on the leached meltwater plain, indicates a forest dominated by hazel and birch^33.^. The grassy openings covered between 20% and 30% of the landscape on a regional scale and up to 40% furthest to the west[^20^](https://paperpile.com/c/YAZ9jC/OUu9t). The openness was partly maintained by forest fires that occurred regularly in this region[^2^](https://paperpile.com/c/YAZ9jC/wsxTk). On the moraine areas in northern Jutland, the forest composition was similar to the primary forest in eastern Denmark, yet, with a more open character[^21,22^](https://paperpile.com/c/YAZ9jC/gPTMk+rAJo1).

These different forest development sequences highlight the marked geographical variation in the forest composition in Denmark during the Atlantic period (*7000-3900 cal. BC*). A variation that can mainly be attributed to different soil conditions. A common feature of all the areas is a marked decrease in elm pollen around *4000 cal. BC*. The decline of elm roughly coincides with traces of the first farmers detected in the archaeological record. Human activity has therefore been connected to the elm decline and especially leaf-feeding of cattle as the primary cause[^12^](https://paperpile.com/c/YAZ9jC/nuLCz). Today, however, elm disease is recognized to be the cause of the elm decline, which has now been detected over large parts of Europe and appears to have occurred over a very short number of years[^23–25^](https://paperpile.com/c/YAZ9jC/tMdqV+dMHlm+cAxch).

#### Early Funnel Beaker Culture (FBC) *3900-3500 cal. BC*

With the occurrence of the first farmers, significant changes in forest composition were initiated. In Lake Højby, there is a marked decrease in lime, probably because the first farmers preferred the high-lying and drier soils also preferred by lime. With the clearing of the lime dominated forest, grasses, oak, alder, willow (*Salix*) and hazel temporarily increased, as a secondary succession in the cleared areas. Despite these major changes in the composition of the forest, only an insignificant change in the ratio between forest and open land is observed. The absence of species indicating grazing, such as ribwort plantain and sheep’s sorrel (*Rumex acetosella*) and other herbs associated with open and grazed landscapes indicates that large herds of free-ranging livestock were not common, despite the fact that the archaeological record contains bones of cows, pigs and sheep/goats[^26,27^](https://paperpile.com/c/YAZ9jC/9BPki+1ELkB). Therefore, these animals were part of the earliest farming economies, but this did not significantly affect the vegetation composition in terms of openness. One possible explanation is that pigs, as well as sheep/goats, were the primary animals in the first century of the Neolithic, while cattle farming was less significant [^e.g. 13^](https://paperpile.com/c/YAZ9jC/5ffiD/?prefix=e.g.). From around *3800 cal. BC*, the distinct increase in birch is caused by the farmers' clearing the forest with fire (Fig. S6.2). Birch has a competitive advantage on the newly burned areas, and the increase in birch cover follows an increase in the charcoal concentration in the lake sediments, suggesting that regular burning was used as an integrated agricultural strategy. This phase covers the period from *3800 to 3400 cal. BC*. Repeated burning of the forest is also supported by pollen preserved under the oldest long barrows. Here, birch pollen dominates, many of which show signs of having been exposed to strong heat, demonstrating that the successional birch groves were burned in subsequent swidden episodes[^14^](https://paperpile.com/c/YAZ9jC/1JKpK).

Between *3650 cal. BC* and the onset of the Middle FBC at *3500 cal. BC*, deforestation and burning intensified and a very open landscape formed, with less than 15% tree cover locally around Lake Højby. At the same time, the first cereal pollen and grazing indicators such as ribwort plantain and sheep’s sorrel occur in the diagram. Studies of plant macrofossils from a number of Danish FBC archaeological sites show that a variety of cereals were grown such as einkorn, emmer, bread / dwarf wheat, as well as naked and hulled barley[^28,29^](https://paperpile.com/c/YAZ9jC/IV3eh+unyac). Macrofossil studies indicate that these cultivars were present from the beginning of the Neolithic. The extensive deforestation and burning observed at Lake Højby are seen in large parts of eastern Denmark, and resulted in a very open landscape, while this development is much less pronounced in western and northern Denmark[^13^](https://paperpile.com/c/YAZ9jC/5ffiD).

#### Middle FBC *3500-3200 cal. BC*

Around Lake Højby the opening of the landscape only existed for a short time period before the forest increased. Especially hazel increased, whilst the open-ground taxa like grasses and ribwort plantain decreased (Fig. S6.2). Around *3400 cal. BC*, birch declines markedly, which may indicate that swidden cultivation as an agricultural practice declined. The continued presence of grazing indicators (ribwort plantain and grasses) indicate that cattle had become a much more important part of the agricultural package. However, it also indicates that grazing pressure was not intensive enough to prevent regrowth of the forest on areas that had been cleared at the end of the previous period. In contrast, grazing pressure had been high enough to form a hazel-dominated forest as hazel, unlike birch, produces new shoots when browsed. It must be concluded that extensive ‟forest grazingʺ was common over a fairly large area. This strategy dominated for about 400 years and a similar vegetation development has been demonstrated in other studies in eastern Denmark. This development is also supported by pollen records from contemporary long barrows and dolmens[^14^](https://paperpile.com/c/YAZ9jC/1JKpK). Despite increasing forestation, the presence of cereal pollen shows local cultivation.

#### Late FBC *3200-2800 cal. BC*

The development that started in the Middle FBC culminated around *3000 cal. BC*, where hazel reached a coverage of approximately 60% and the primary forest almost 40%. Thereafter, hazel is drastically reduced, and within only a hundred years hazel became an insignificant component of the forest composition. The former hazel-dominated landscape is transformed into open grazing land, dominated by grasses, ribwort plantain and sheep’s sorrel. In addition to this development, cereal cultivation seems to be intensified, a development that is supported by pollen studies of soil profiles below passage graves[^14^](https://paperpile.com/c/YAZ9jC/1JKpK).

A significant part of the grazing land was reforested with climax forest during the transition to the Single Grave Culture (SGC). A likely interpretation is that fields and grazing areas became more permanent, in contrast to the previous periods when agriculture was based on regular claiming of new land.

#### SGC *2800-2400 cal. BC*

The development of permanent open grazing areas is observed at the transition to SGC in many regions. However, it is particularly pronounced in Northern and Western Jutland[^13^](https://paperpile.com/c/YAZ9jC/5ffiD). Here, the forest is cleared, creating the open and forest-poor landscape that has dominated the region until the 19th century AD. In eastern Denmark, there are several examples of deforestations in the SGC [^e.g. 30^](https://paperpile.com/c/YAZ9jC/F4Ylb/?prefix=e.g.), but in many areas the forest dominated during the SGC, as we see at Lake Højby, and remained dominant until the Late Bronze Age[^31^](https://paperpile.com/c/YAZ9jC/bUAb0). During the SGC, barley became the most common cereal variety and the diversity of the crops grown seems to decline compared to previous periods[^32^](https://paperpile.com/c/YAZ9jC/uYHzk).

References

1. [Iversen, J. *The Development of Denmark’s Nature since the Last Glacial*. Geological Survey of Denmark Række 5, Vol. **7** (1973).](http://paperpile.com/b/YAZ9jC/usN1l)

2. [Odgaard, B. V. The Holocene vegetation history of northern West Jutland. (1994).](http://paperpile.com/b/YAZ9jC/wsxTk)

3. [Rasmussen, P. Mid- to late-Holocene land-use change and lake development at Dallund Sø, Denmark: vegetation and land-use history inferred from pollen data. *Holocene* **15**, 1116–1129 (2005).](http://paperpile.com/b/YAZ9jC/wh8CP)

4. [Odgaard, B. & Nielsen, A. B. Udvikling i arealdækningen i perioden 0-1850. Pollen og landskabshistorie. in *41-58. Danske landbrugslandskaber gennem 2000 år – fra digevoldinger til støtteordninger* (eds. Odgaard, B. og R. & J.r.) (Aarhus Universitetsforlag, 2009).](http://paperpile.com/b/YAZ9jC/Eo3cb)

5. [Mortensen, M. F., Henriksen, P. S. & Bennike, O. Living on the good soil: relationships between soils, vegetation and human settlement during the late Allerød time period in Denmark. *Veg. Hist. Archaeobot.* **23**, 195–205 (2014).](http://paperpile.com/b/YAZ9jC/tzwlY)

6. [Andersen, S. T. History of the terrestrial environment in the Quaternary of Denmark. *Bull. Geol. Soc. Den.* **41**, 219–228 (1994).](http://paperpile.com/b/YAZ9jC/fw3iK)

7. [Mortensen, M. F. *et al.* Late-glacial vegetation development in Denmark – new evidence based on macrofossils and pollen from Slotseng, a small-scale site in southern Jutland. *Quaternary Sciences Reviews* **30**, 2534–3550 (2011).](http://paperpile.com/b/YAZ9jC/3ckLU)

8. [Iversen, J. Land occupation in Denmark’s Store Age. *Danmarks Geologiske Undersøgelse, Series* **II**, 68 (1941).](http://paperpile.com/b/YAZ9jC/PGVXz)

9. [Iversen, J. The influence of Prehistoric man on vegetation. *Danmarks Geologiske Undersøgelse, Series* **IV**, 25 (1949).](http://paperpile.com/b/YAZ9jC/Svdhd)

10. [Iversen, J. Problems of the early post-glacial forest development in Denmark. *Danmarks Geologiske Undersøgelse, Series* **4**, 32 (1960).](http://paperpile.com/b/YAZ9jC/OOQkV)

11. [Troels-Smith, J. *Ertebøllekultur - Bondekultur. Resultater af de sidste 10 års Undersøgelser i Aamosen*. 5–62 (Aarbøger for Nordisk Oldkyndighed og Historie, 1953).](http://paperpile.com/b/YAZ9jC/yrkY5)

12. [Troels-Smith, J. Ivy, mistletoe and elm. Climate indicators – fodder Plants: a contribution to the interpretation of the pollen zone border VII-VIII. *Danmarks Geologiske Undersøgelse* **4**, 32 (1960).](http://paperpile.com/b/YAZ9jC/nuLCz)

13. [Andersen, S. *Early agriculture*. 88–91 (The Royal Society of Northern Antiquaries and the Jutland Archaeological Society. Aarhus Universitetsforlag, 1993).](http://paperpile.com/b/YAZ9jC/5ffiD)

14. [Andersen, S. T. The cultural landscape of megalithic tombs in Denmark, reconstructed by soil pollen analysis. *Danish Journal of Archaeology* **1**, 39–44 (2012).](http://paperpile.com/b/YAZ9jC/1JKpK)

15. [Iversen, J. Naturens udvikling siden sidste istid. in *Danmarks Natur I, Landskabernes opståen* (eds. Nørrevang, A. og M. & T.j.) 345–445 (1967).](http://paperpile.com/b/YAZ9jC/vbjl1)

16. [Hede, M. U. Holocene climate and environmental changes recorded in high-resolution lake sediments from Højby Sø, Denmark. (University of Copenhagen, 2008).](http://paperpile.com/b/YAZ9jC/JXPV6)

17. [Sugita, S. a. Theory of quantitative reconstruction of vegetation II: all you need is LOVE. *Holocene* **17**, 243–357 (2007).](http://paperpile.com/b/YAZ9jC/jdXyn)

18. [Sugita, S. b. Theory of quantitative reconstruction of vegetation I: pollen from large sites REVEALS regional vegetation composition. *Holocene* **17**, 229–241 (2007).](http://paperpile.com/b/YAZ9jC/ICaYO)

19. [Nielsen, A. B. Using Pollen Data and Models to Assess Landscape Structure and the Role of Grazers in Pre-agricultural Denmark. in *Ancient Woodlands and Trees: A Guide for Landscape Planners and Forest Managers. International Union of Forest Research Organizations (IUFRO* (eds. Çolak, A. H., Kırca, S. & Rotherham, I. D.) 242–248 (2018).](http://paperpile.com/b/YAZ9jC/EkB7z)

20. [Nielsen, A. B. *et al.* Quantitative reconstructions of changes in regional openness in north-central Europe reveal new insights into old questions. *Quat. Sci. Rev.* **47**, 131–147 (2012).](http://paperpile.com/b/YAZ9jC/OUu9t)

21. [Kristiansen, K., Melheim, L., Bech, J.-H., Mortensen, M. F. & Frei, K. M. Thy at the Crossroads: A Local Bronze Age Community’s Role in a Macro-Economic System. in *Contrasts of the Nordic Bronze Age* 269–282 (Brepols Publishers, 2020).](http://paperpile.com/b/YAZ9jC/gPTMk)

22. [Haak, W. *et al.* The Corded Ware Complex in Europe in light of current archeogenetic and environmental evidence.](http://paperpile.com/b/YAZ9jC/rAJo1)

23. [Rasmussen, L. W. Kainsbakke, en kystboplads i yngre stenalder. 9–69 (1991).](http://paperpile.com/b/YAZ9jC/tMdqV)

24. [Peglar, S. M. The mid-Holocene Ulmus decline at Diss Mere, Norfolk, UK: a year-by-year pollen stratigraphy from annual laminations. *Holocene* **3**, 1–13 (1993).](http://paperpile.com/b/YAZ9jC/dMHlm)

25. [Peglar, S. M. & Birks, H. J. B. The mid-Holocene Ulmus fall at Diss Mere, South-East England – disease and human impact? *Veg. Hist. Archaeobot.* **2**, 61–68 (1993).](http://paperpile.com/b/YAZ9jC/cAxch)

26. [Noe-Nygaard, N., Price, T. D. & Hede, S. U. Diet of aurochs and early cattle in southern Scandinavia: evidence from 15N and 13C stable isotopes. *J. Archaeol. Sci.* **32**, 855–871 (2005).](http://paperpile.com/b/YAZ9jC/9BPki)

27. [Fischer, A., Gotfredsen, A. B., Meadows, J., Pedersen, L. & Stafford, M. The Rødhals kitchen midden – marine adaptations at the end of the Mesolithic world. *Journal of Archaeological Science: Reports* **39**, 103102 (2021).](http://paperpile.com/b/YAZ9jC/1ELkB)

28. [Robinson, D. E. Dyrkede planter fra Danmarks forhistorie. Arkæologiske udgravninger i Danmark 1993, Det Arkæologiske Nævn. 20–39 (1994).](http://paperpile.com/b/YAZ9jC/IV3eh)

29. [Nielsen, P. O. & Nielsen, F. O. S. *First Farmers on the Island of Bornholm*. (The Royal Society of Northern Antiquaries and University Press of Southern Denmark, 2020).](http://paperpile.com/b/YAZ9jC/unyac)

30. [Rasmussen, P. & Olsen, J. Soil erosion and land-use change during the last six millennia recorded in lake sediments of Gudme Sø, Fyn. *Denmark. Review of Survey activities* 37–40 (2009).](http://paperpile.com/b/YAZ9jC/F4Ylb)

31. [Odgaard, B. V. & Rasmussen, P. Origin and temporal development of macro-scale vegetation patterns in the cultural landscape of Denmark. *J. Ecol.* **88**, 733–748 (2000).](http://paperpile.com/b/YAZ9jC/bUAb0)

32. [Robinson, D. E. Neolithic and Bronze Age Agriculture in Southern Scandinavia - Recent Archaeolobotanical Evidence from Denmark. *Environ. Archaeol.* **8**, 145–165 (2003).](http://paperpile.com/b/YAZ9jC/uYHzk)LP

33. [Haak, W. *et al.* 5: The corded ware complex in Europe in light of current archaeogenetic and environmental evidence. *The Indo-European Puzzle Revisited: Integrating Archaeology, Genetics, and Linguistics* 63 (2023).](http://paperpile.com/b/U7w4HD/CEVMT)
