# Supplementary material for: 100 ancient genomes show repeated population turnovers in Neolithic Denmark
Source: Nature. 2024 Jan 10;625(7994):329–37. doi: 10.1038/s41586-023-06862-3 (PMC10781617; doi:10.1038/s41586-023-06862-3)
Supplement: Supplementary file 2 — Reporting Summary [file 41586_2023_6862_MOESM2_ESM.pdf]

nature portfolio

Corresponding author(s): Morten Allentoft, Martin Sikora, Eske Willerslev

Last updated by author(s): Oct 18, 2023

Reporting Summary

Nature Portfolio wishes to improve the reproducibility of the work that we publish. This form provides structure for consistency and transparency in reporting. For further information on Nature Portfolio policies, see our Editorial Policies and the Editorial Policy Checklist.

Statistics

For all statistical analyses, confirm that the following items are present in the figure legend, table legend, main text, or Methods section.

n/a	Confirmed	
☐	☒	The exact sample size (*n*) for each experimental group/condition, given as a discrete number and unit of measurement
☐	☒	A statement on whether measurements were taken from distinct samples or whether the same sample was measured repeatedly
☐	☒	The statistical test(s) used AND whether they are one- or two-sided<br>*Only common tests should be described solely by name; describe more complex techniques in the Methods section.*
☐	☒	A description of all covariates tested
☐	☒	A description of any assumptions or corrections, such as tests of normality and adjustment for multiple comparisons
☐	☒	A full description of the statistical parameters including central tendency (e.g. means) or other basic estimates (e.g. regression coefficient) AND variation (e.g. standard deviation) or associated estimates of uncertainty (e.g. confidence intervals)
☐	☒	For null hypothesis testing, the test statistic (e.g. *F*, *t*, *r*) with confidence intervals, effect sizes, degrees of freedom and *P* value noted<br>*Give P values as exact values whenever suitable.*
☐	☒	For Bayesian analysis, information on the choice of priors and Markov chain Monte Carlo settings
☐	☒	For hierarchical and complex designs, identification of the appropriate level for tests and full reporting of outcomes
☐	☒	Estimates of effect sizes (e.g. Cohen's *d*, Pearson's *r*), indicating how they were calculated

Our web collection on statistics for biologists contains articles on many of the points above.

Software and code

Policy information about availability of computer code

Data collection	Sequencing data and metadata pertaining to the sequencing of ancient genomes is managed on secure servers at the Globe Institute, University of Copenhagen, and via the Illumina Inc. BaseSpace platform. Accelerator Mass Spectrometry data and associated metadata is managed at the Department of Historical Studies, University of Gothenberg.
Data analysis	Custom scripts used to apply chromopainter from large-scale phased data are available at https://github.com/will-camb/Nero/tree/master/scripts/cp_panel_scripts. All other analyses relied upon available software which has been fully referenced in the manuscript and detailed in the relevant supplementary notes. These comprise:<br>CASAVA (v.1.8.2)<br>AdapterRemoval (v.2.1.3)<br>Picard (v.1.127)<br>GATK (v.3.3.0)<br>Samtools (1.9)<br>Samtools calmd (v.1.10)<br>pysam (https://github.com/pysam-developers/pysam)<br>BEDtools (v.2.23.0)<br>mapDamage2.0 (v2.2.1)<br>BWA (0.7-17)<br>Schmutzi (VERSION?)<br>ContamMix (VERSION?)<br>ANGSD (0.938)<br>GLIMPSE (v1.0.1)

Beagle (v4.1)
BCFtools 1.10
MAFFT (v7.490)
RAxML-ng (v1.1.0)
ry_compute (v0.4)
EPA-ng (0.3.8)
NgsRelate (v2)
ADMIXTURE (1.3.0)
smartpca
GCTA
R version 4.0
ADMIXTOOLS2
gstat (2.0-9)
IBDseq (version r1206)
GenomicRanges (3.15)
leidenAlg (v1.01, https://github.com/kharchenkolab/leidenAlg)
igraph (0.9.9)
limSolve (1.5.6)
Scikit-learn (0.21.2)
Chromopainter (0.0.4)
fineSTRUCTURE (0.0.5)
QCTOOL v2 (https://www.well.ox.ac.uk/~gav/qctool_v2/)
ArcGIS Online (www.arcgis.com)
OxCal v4.4
LRA v0.1.0 (https://github.com/petrkunes/LRA)

For manuscripts utilizing custom algorithms or software that are central to the research but not yet described in published literature, software must be made available to editors and reviewers. We strongly encourage code deposition in a community repository (e.g. GitHub). See the Nature Portfolio guidelines for submitting code & software for further information.

Data

Policy information about availability of data

All manuscripts must include a data availability statement. This statement should provide the following information, where applicable:

- Accession codes, unique identifiers, or web links for publicly available datasets
- A description of any restrictions on data availability
- For clinical datasets or third party data, please ensure that the statement adheres to our policy

Data in this study is released in the accompanyinh publication 'Population Genomics of Postglacial Western Eurasia', where raw sequencing data and alignments will be made available on prior or simultaneous publication.

Human research participants

Policy information about studies involving human research participants and Sex and Gender in Research.

Reporting on sex and gender	No living or recently deceased human research participants were affected by this study
Population characteristics	*Describe the covariate-relevant population characteristics of the human research participants (e.g. age, genotypic information, past and current diagnosis and treatment categories). If you filled out the behavioural & social sciences study design questions and have nothing to add here, write "See above."*
Recruitment	*Describe how participants were recruited. Outline any potential self-selection bias or other biases that may be present and how these are likely to impact results.*
Ethics oversight	*Identify the organization(s) that approved the study protocol.*

Note that full information on the approval of the study protocol must also be provided in the manuscript.

Field-specific reporting

Please select the one below that is the best fit for your research. If you are not sure, read the appropriate sections before making your selection.

☐ Life sciences ☐ Behavioural & social sciences ☒ Ecological, evolutionary & environmental sciences

For a reference copy of the document with all sections, see nature.com/documents/nr-reporting-summary-flat.pdf

Ecological, evolutionary & environmental sciences study design

All studies must disclose on these points even when the disclosure is negative.

Study description	The study undertook population genomics analysis of ancient individuals from Denmark, on data from the accompanying publication 'Population Genomics of Stone Age Eurasia', in addition to stable isotope (13C, 15N, 87Sr) and radiocarbon data analysis.
Research sample	100 human (Homo_sapiens) individuals from archaeological sites across Denmark, all >1000 years old.
Sampling strategy	We sampled primarily individuals from the Mesolithic and Neolithic periods from Danish archaeological sites; sampling was restricted to samples where sufficient DNA preservation was available for genome-scale analysis.
Data collection	Ancient genomic data was collected following established laboratory protocols, designed to minimise sample destruction and avoid contamination. These are more appropriately detailed in the accompanying publication describing the generation of this dataset.
Timing and spatial scale	The oldest sample has a corrected radiocarbon age of 10,465years; the most recent has a corrected radiocarbon age of 3297years. All samples originate from the present-day country of Denmark.
Data exclusions	Data from the accompanying publication 'Population Genomics of Stone Age Eurasia' was subset to only include samples from Denmark.
Reproducibility	Preserved archaeological remains are unique and rare, therefore replication was generally not undertaken. Data quality and uncertainty (e.g. contamination) was accounted for in computational analyses to assess robustness of inferences, and all methods and data are made available for future replication.
Randomization	Randomization of sampling of archaeological remains is not applicable in this case.
Blinding	Blinding was not applicable to this study.

Did the study involve field work? ☐ Yes ☒ No

Reporting for specific materials, systems and methods

We require information from authors about some types of materials, experimental systems and methods used in many studies. Here, indicate whether each material, system or method listed is relevant to your study. If you are not sure if a list item applies to your research, read the appropriate section before selecting a response.

Materials & experimental systems

n/a	Involved in the study
☒	☐ Antibodies
☒	☐ Eukaryotic cell lines
☐	☒ Palaeontology and archaeology
☒	☐ Animals and other organisms
☒	☐ Clinical data
☒	☐ Dual use research of concern

Methods

n/a	Involved in the study
☒	☐ ChIP-seq
☒	☐ Flow cytometry
☒	☐ MRI-based neuroimaging

Palaeontology and Archaeology

Specimen provenance	Details of specimen provenance are provided in the accompanying publication 'Population Genomics of Stone Age Eurasia', where the full sample dataset is presented.
Specimen deposition	See above; all specimens studied are available upon direct contact/request to the archaeologists, curators or officials responsible for their curation at the organisation where they are held.

Dating methods	272 novel radiocarbon dates were generated at the 14CHRONO laboratory, Queen's University Belfast (242 samples), at the Oxford Radiocarbon Accelerator Unit (ORAU) laboratory (24 samples) and at the Keck-CCAMS Group, Irvine, California, USA (6 samples).

☒ Tick this box to confirm that the raw and calibrated dates are available in the paper or in Supplementary Information.

Ethics oversight	Sampling was undertaken with the ethical approval of museums and institutions providing samples or facilities.

Note that full information on the approval of the study protocol must also be provided in the manuscript.
